# Supplementary material for: Retrospective secondary data analysis to identify high-cost users in inpatient department of hospitals in Thailand, a middle-income country with universal healthcare coverage
Source: BMJ Open. 2021 Jul 27;11(7):e047330. doi: 10.1136/bmjopen-2020-047330 (PMC8319992; doi:10.1136/bmjopen-2020-047330)
Supplement: Supplementary data [file bmjopen-2020-047330supp001.pdf]

Supplementary figure 1 Different health regions in Thailand

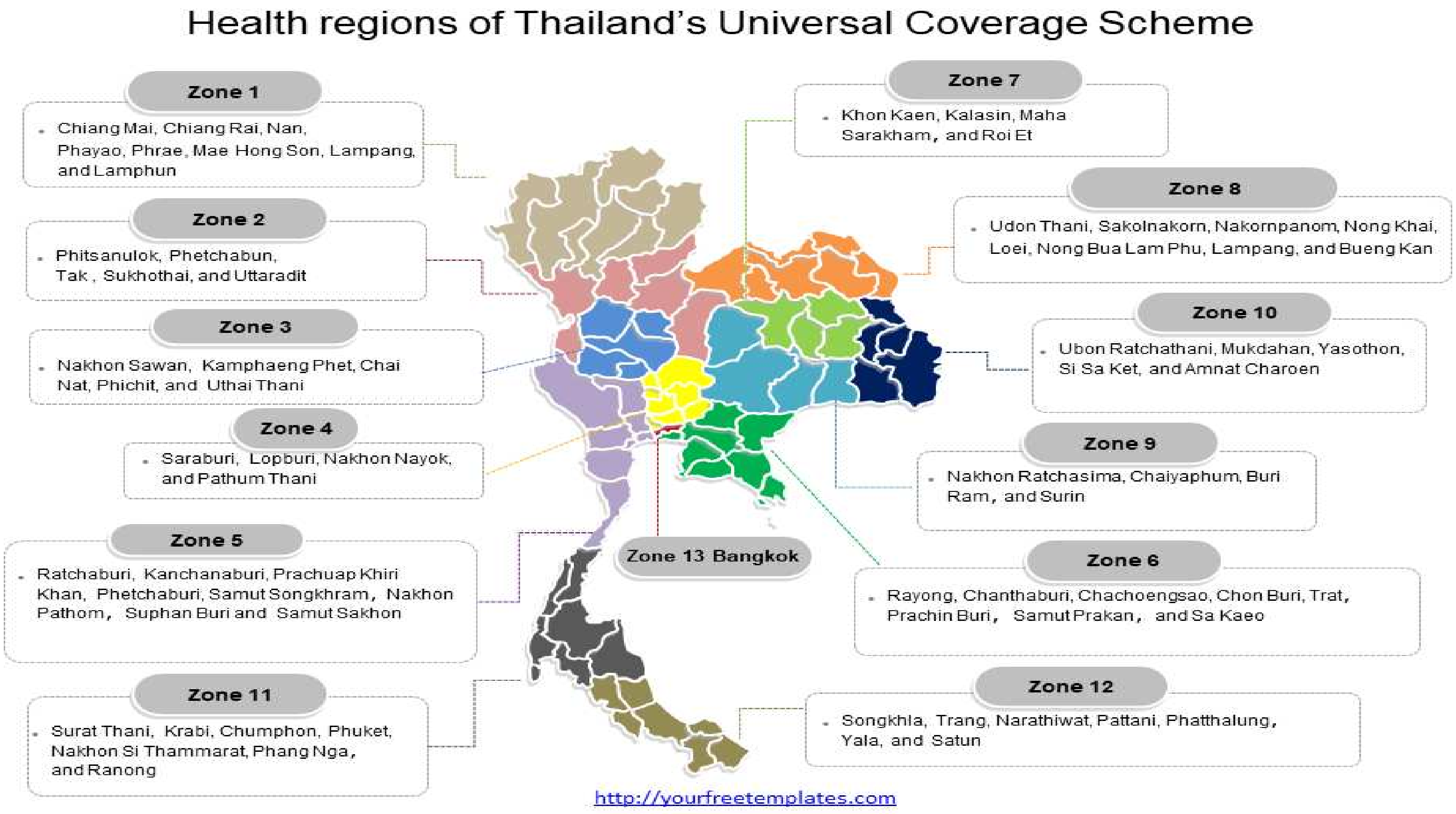

Supplementary figure 2 Flowchart of the data management process

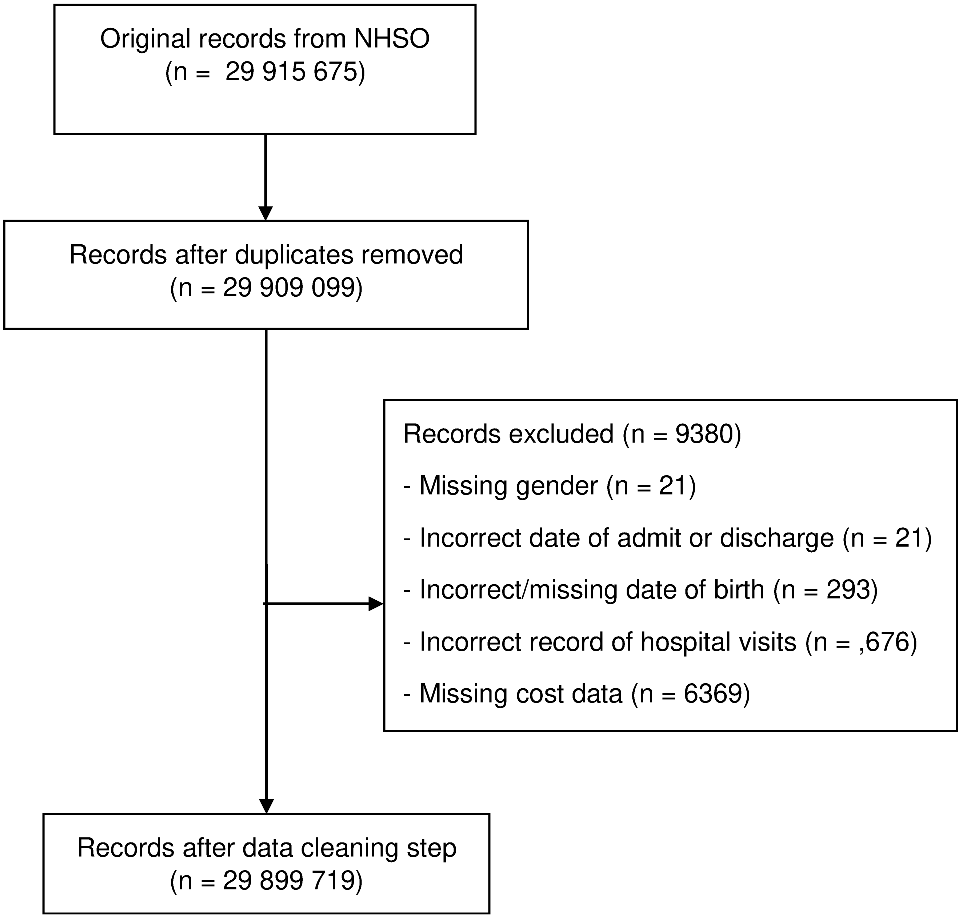

Supplementary table 1 Marginal effect of Predictors associated with high-cost users in UCS

	2014	2015	2016	2017	2018
	**Prob (95%CI)**	**Prob (95%CI)**	**Prob (95%CI)**	**Prob (95%CI)**	**Prob (95%CI)**
Age	0.00036 (0.00034, 0.00037)	0.00033 (0.00032, 0.00034)	0.00038 (0.00037, 0.00039)	0.00036 (0.00035, 0.00037)	0.00037 (0.00036, 0.00038)
Gender (ref: female)	-0.0114 (-0.0118, -0.0110)	-0.0110 (-0.0114, -0.0106)	-0.006 (-0.011, -0.010)	-0.0108 (-0.0111, -0.0104)	-0.0102 (-0.0106, -0.0098)
Regional hospitals	0.048 (0.047, 0.049)	0.048 (0.047, 0.049)	0.050 (0.049, 0.050)	0.046 (0.045, 0.047)	0.047 (0.046, 0.047)
Hospital type (ref: other clinics)					
General hospitals, community hospitals, and other MOPH agencies	0.0036 (0.0030, 0.0042)	0.0053 (0.0047, 0.0058)	0.0035 (0.0029, 0.0041)	0.0037 (0.0031, 0.0043)	0.0036 (0.0031, 0.0042)
Non MOPH agencies such as university hospitals	0.106 (0.104, 0.107)	0.134 (0.132, 0.136)	0.119 (0.117, 0.121)	0.114 (0.113, 0.117)	0.108 (0.106, 0.110)
Private hospitals	0.030 (0.028, 0.031)	0.067 (0.065, 0.069)	0.058 (0.056, 0.060)	0.054 (0.052, 0.057)	0.053 (0.051, 0.055)
Hospital zone: (ref: hospitals outside Bangkok)					
Bangkok	0.060 (0.058, 0.062)	0.089 (0.087, 0.092)	0.074 (0.072, 0.076)	0.073 (0.071, 0.076)	0.065 (0.063, 0.08)
Primary diagnosis					
Neoplasm	0.083 (0.081, 0.085)	0.078 (0.077, 0.080)	0.073 (0.071, 0.075)	0.074 (0.072, 0.076)	0.075 (0.074, 0.077)
Diseases of circulatory system	0.071 (0.069, 0.072)	0.075 (0.073, 0.076)	0.079 (0.077, 0.081)	0.082 (0.080, 0.084)	0.082 (0.080, 0.083)
Diseases of respiratory system	0.0181 (0.0174, 0.0187)	0.0104 (0.0098, 0.0111)	0.0134 (0.0128, 0.0140)	0.0072 (0.0066, 0.0078)	0.0075 (0.0069, 0.0081)
Diseases of the digestive system	-0.0043 (-0.0050, -0.0037)	-0.0061 (-0.0067, -0.0056)	-0.0050 (-0.0056, -0.0044)	-0.0067 (-0.073, -0.0061)	-0.0074 (-0.0080, -0.0068)
Injury, or poisoning and certain other consequences of external causes	0.041 (0.040, 0.042)	0.046 (0.045, 0.047)	0.044 (0.043, 0.045)	0.040 (0.039, 0.040)	0.041 (0.041, 0.042)
Death	0.104 (0.103, 0.106)	0.085 (0.083, 0.086)	0.086 (0.084, 0.087)	0.074 (0.072, 0.075)	0.066 (0.065, 0.068)
Number of primary diagnoses	0.0067 (0.0064, 0.0070)	0.0082 (0.0079, 0.0085)	0.0080 (0.0077, 0.0083)	0.0088 (0.0085, 0.0091)	0.0093 (0.0091, 0.0096)
Number of visits in a year	0.0182 (0.0181, 0.0184)	0.0166 (0.0165, 0.0168)	0.0164 (0.0162, 0.0165)	0.0167 (0.0165, 0.0168)	0.0164 (0.0162, 0.0165)

Prob, Probability; 95% CI, 95% confidence interval

Supplementary table 2 Relative risk ratio of factors predicting being high-cost users and persistent high-cost users

**Factors**	**Factors predicting being HCUs RRR (95% CI)**	**Factors predicting being persistent HCUs RRR (95% CI)**
Age	1.044 (1.043, 1.045)	1.083 (1.081, 1.086)
Age*Age	0.99977 (0.99976, 0.99978)	0.99953 (0.99951, 0.99955)
Gender (ref: female)	0.691 (0.680, 0.702)	0.803 (0.774, 0.834)
Hospital type (ref: other clinics)		
Regional hospitals	2.834 (2.803, 2.864)	3.190 (3.118, 3.263)
General hospitals, community hospitals, and other MOPH agencies	1.089 (1.075, 1.103)	0.855 (0.831, 0.880)
Non MOPH agencies such as university hospitals	5.560 (5.463, 5.660)	10.235 (9.933, 10.548)
Private hospitals	2.290 (2.235, 2.346)	3.178 (3.041, 3.322)
Hospital zone: (ref: hospitals located outside Bangkok)		
Bangkok	4.401 (4.239, 4.569)	8.153 (7.635, 8.705)
Primary diagnosis		
Neoplasm	7.914 (7.535, 8.312)	43.551 (40.338, 47.020)
Diseases of circulatory system	7.240 (6.961, 7.528)	12.430 (11.521, 13.411)
Diseases of respiratory system	1.301 (1.268, 1.334)	2.214 (2.083, 2.352)
Diseases of the digestive system	0.727 (0.703, 0.750)	0.951 (0.890, 1.018)
Injury, or poisoning and certain other consequences of external causes	1.988 (1.934, 2.043)	2.783 (2.604, 2.974)
Comorbidity (Charlson Comorbidity Index)	1.124 (1.119, 1.130)	1.211 (1.202, 1.219)
Death	3.032 (2.927, 3.141)	3.633 (3.427, 3.850)
Number of primary diagnoses	1.706 (1.697, 1.714)	1.754 (1.741, 1.767)
Number of visits in a year	1.061 (1.059, 1.062)	1.165 (1.163, 1.166)
Neoplasm*Death	0.563 (0.538, 0.589)	0.601 (0.565, 0.640)
Diseases of circulatory system*Death	0.506 (0.489, 0.524)	0.555 (0.526, 0.585)
Diseases of respiratory system*Death	1.391 (1.344, 1.440)	1.787 (1.696, 1.883)
Diseases of the digestive system*Death	1.122 (1.082, 1.164)	1.224 (1.160, 1.292)
Injury, or poisoning and certain other consequences of external causes*Death	0.677 (0.649, 0.706)	0.768 (0.723, 0.816)
Neoplasm*Age	0.984 (0.983, 0.985)	0.964 (0.963, 0.966)
Diseases of circulatory system*Age	0.9807 (0.9801, 0.9813)	0.976 (0.974, 0.977)
Diseases of respiratory system*Age	0.9981 (0.9977, 0.9985)	0.990 (0.989, 0.991)
Diseases of the digestive system*Age	1.0009 (1.0003, 1.0014)	0.9966 (0.9955, 0.9977)
Injury, or poisoning and certain other consequences of external causes*Age	0.9967 (0.9962, 0.9972)	0.9937 (0.9926, 0.9948)
Neoplasm*Gender	0.844 (0.822, 0.875)	0.690 (0.659, 0.722)

**Factors**	**Factors predicting being HCUs RRR (95% CI)**	**Factors predicting being persistent HCUs RRR (95% CI)**
Diseases of circulatory system*Gender	1.010 (0.990, 1.030)	0.832 (0.800, 0.865)
Diseases of respiratory system*Gender	0.952 (0.933, 0.972)	0.824 (0.793, 0.857)
Diseases of the digestive system*Gender	1.064 (1.041, 1.088)	0.965 (0.927, 1.005)
Injury, or poisoning and certain other consequences of external causes*Gender	1.190 (1.163, 1.216)	1.229 (1.178, 1.283)
Hospital zone: Bangkok* Regional hospitals	0.545 (0.526, 0.566)	0.519 (0.492, 0.549)
Hospital zone: Bangkok* General hospitals, community hospitals, and other MOPH agencies	1.312 (1.272, 1.354)	1.561 (1.480, 1.647)
Hospital zone: Bangkok* Non MOPH agencies	0.276 (0.267, 0.286)	0.189 (0.180, 0.200)
Hospital zone: Bangkok* Private hospitals	0.421 (0.404, 0.438)	0.300 (0.279, 0.319)
Constant	0.00218 (0.00213, 0.00224)	0.000030 (0.000028, 0.000032)

95% CI, 95% confidence interval; HCUs, high-cost users; RRR, relative risk ratio.
