# Supplementary material for: Retrospective secondary data analysis to identify high-cost users in inpatient department of hospitals in Thailand, a middle-income country with universal healthcare coverage
Source: BMJ Open. 2021 Jul 27;11(7):e047330. doi: 10.1136/bmjopen-2020-047330 (PMC8319992; doi:10.1136/bmjopen-2020-047330)
Supplement: Author's manuscript [file bmjopen-2020-047330.draft_revisions.pdf]

BMJ Open

BMJ Open is committed to open peer review. As part of this commitment we make the peer review history of every article we publish publicly available.

When an article is published we post the peer reviewers' comments and the authors' responses online. We also post the versions of the paper that were used during peer review. These are the versions that the peer review comments apply to.

The versions of the paper that follow are the versions that were submitted during the peer review process. They are not the versions of record or the final published versions. They should not be cited or distributed as the published version of this manuscript.

BMJ Open is an open access journal and the full, final, typeset and author-corrected version of record of the manuscript is available on our site with no access controls, subscription charges or pay-per-view fees (http://bmjopen.bmj.com).

If you have any questions on BMJ Open's open peer review process please email info.bmjopen@bmj.com

BMJ Open

High-cost healthcare users in a middle-income country with universal healthcare coverage: The search for an expensive necessity

Journal:	*BMJ Open*
Manuscript ID	bmjopen-2020-047330
Article Type:	Original research
Date Submitted by the Author:	27-Nov-2020
Complete List of Authors:	Rattanavipapong, Waranya; Health Intervention and Technology Assessment Program, Health<br>Yi, Wang; National University of Singapore<br>Butchon, Rukmanee; Health Intervention and Technology Assessment Program, Health<br>Kittiratchakool, Nitichen; Health Intervention and Technology Assessment Program<br>Thammatacharee , Jadej; National Health Security Office<br>Teerawattananon, Yot; Health Intervention and Technology Assessment Program, Health; National University of Singapore<br>Isaranuwatchai, Wanrudee; Health Intervention and Technology Assessment Program, Health
Keywords:	HEALTH ECONOMICS, Health policy < HEALTH SERVICES ADMINISTRATION & MANAGEMENT, PUBLIC HEALTH, STATISTICS & RESEARCH METHODS

SCHOLARONE™
Manuscripts

I, the Submitting Author has the right to grant and does grant on behalf of all authors of the Work (as defined in the below author licence), an exclusive licence and/or a non-exclusive licence for contributions from authors who are: i) UK Crown employees; ii) where BMJ has agreed a CC-BY licence shall apply, and/or iii) in accordance with the terms applicable for US Federal Government officers or employees acting as part of their official duties; on a worldwide, perpetual, irrevocable, royalty-free basis to BMJ Publishing Group Ltd ("BMJ") its licensees and where the relevant Journal is co-owned by BMJ to the co-owners of the Journal, to publish the Work in this journal and any other BMJ products and to exploit all rights, as set out in our licence.

The Submitting Author accepts and understands that any supply made under these terms is made by BMJ to the Submitting Author unless you are acting as an employee on behalf of your employer or a postgraduate student of an affiliated institution which is paying any applicable article publishing charge ("APC") for Open Access articles. Where the Submitting Author wishes to make the Work available on an Open Access basis (and intends to pay the relevant APC), the terms of reuse of such Open Access shall be governed by a Creative Commons licence – details of these licences and which Creative Commons licence will apply to this Work are set out in our licence referred to above.

Other than as permitted in any relevant BMJ Author's Self Archiving Policies, I confirm this Work has not been accepted for publication elsewhere, is not being considered for publication elsewhere and does not duplicate material already published. I confirm all authors consent to publication of this Work and authorise the granting of this licence.

For peer review only

High-cost healthcare users in a middle-income country with universal healthcare coverage: The search for an expensive necessity

Correspondence to Wanrudee Isaranuwatchai; 6th Floor, 6th Building, Department of Health, Ministry of Public Health, Tiwanon Rd., Muang, Nonthaburi 11000, Thailand; wanrudee.i@hitap.net; +662-590-4549, +662-590-4374, +662-590-4375

Authors and Institutions

Waranya Rattanavipapong[1], Wang Yi[2], Rukmanee Butchon[1], Nitichen Kittiratchakool[1], Jadej Thammatacharee[3], Yot Teerawattananon[1], Wanrudee Isaranuwatchai[1]

Author affiliations

[1]Health Intervention and Technology Assessment Program, Nonthaburi, Thailand

[2]Health Intervention and Policy Evaluation Research, Saw Swee Hock School of Public Health, National University of Singapore, Singapore

[3]National Health Security Office, Thailand

[4]Institute or Health Policy, Management and Evaluation, University of Toronto, Canada

Word count: 3,209/4000

For peer review only

For peer review only - http://bmjopen.bmj.com/site/about/guidelines.xhtml

ABSTRACT(251/300 words)

Objectives

The study aims to identify high-cost healthcare users (HCUs) in Thailand including their common characteristics, patterns of health care utilization and expenditure compared to low-cost users, and to explore potential predictors of HCUs so the health care system can be prepared to support the HCUs including those who have increased chance of becoming HCUs.

Design and setting

A retrospective secondary data analysis using hospitalization data from Thailand's universal coverage scheme (UCS)obtained from the National Health Security Office over a 5-year period from October 2014 to September 2019 (fiscal year 2014-2018).

Participants

Study participants included patients who had at least one inpatient admission to hospitals under UCS over the studyperiod.

Results

Over the 5-year period,the top 5% of hospitalized population or HCUs consumes almost 50% of the health expenditure each year. HCUs were more likely to have longer hospital stays, have a higher annual number of visits, and admitted at multiple hospitals each year when compared to the low-cost users (the bottom 50% of hospitalized population). The study further reported that the chance of becoming HCUs is associated with several factors such as increasing age, being male, having comorbidity, and being admitted to hospitals in Bangkok.

Conclusions

This study confirmed that the HCUs phenomenon existed in Thailand, where a majority of inpatient care spending under is concentrated in the top 5% of hospitalized population.Thestudy findingscall attention to potential initiatives that can help monitor the magnitude and trend of HCUs and develop policies to prevent HCUs.

For peer review only - http://bmjopen.bmj.com/site/about/guidelines.xhtml

Strengths and limitations of this study

- This is the first study in Thailand showing an evidence of high-cost users (HCUs) which could help address policy-relevant questions such as who are the HCUs, what supports (in term of inpatient services) do they need, and what are the key factors to help predict future HCUs.
- The study findings (e.g., needs of HCUs) could be used to assist in the planning and development of universal coverage scheme (UCS) in which various stakeholders (e.g. policy makers, public healthcare payer, and health providers).
- The study represents an example of howexisting routinely collected real-world data (from administrative databases) can be used in a middle-income country to potentiallyimprove care and health service, and may be explored by other low- and middle-income countries who are exploring the potential of real-world evidence.
- The analysis was limited by data available; therefore, only hospitalization data were used with limited numbers of potential confounders to explore over the period of five years.Future work could expand to explore other types of services such as outpatient services, rehabilitation, and prevention and promotion programs including other potential confounders and support for data quality process.

Keywords:high-cost users, high-cost, high spending, health care utilization, hospitalization, administrative databases, Thailand

For peer review only

For peer review only - http://bmjopen.bmj.com/site/about/guidelines.xhtml

INTRODUCTION

There is an increasing emphasis on the sustainability of healthcare systems for many reasons including the availability of new costly health technologies, the greater demand in healthcare from aging population, and the government's commitmentto make universal health coverage (UHC) under limited resources.As such, several solutions have been attempted to enhance the efficiency of healthcare systems around the world. These potential solutions include prioritization of health problems and associated interventionsbased on, for example, burden of disease and cost-effectiveness evidence,[1 2] identifying low-value health care that can be omitted without adversely affecting patient outcomes,[3 4] and promoting the use of preventive measures to avoid patients developedchronic conditions.[5 6]

Based on literature review on high-cost users (HCUs) phenomenon in severalcountries,e.g., Japan, United States, Australia, and Canada,[7-12] HCU is generally defined as the top 5% of patients with the highest healthspending in a given year, and low-cost user (LCU) is, on the other hand, accounting to the bottom50% of patients with the lowest healthcare cost. A better understanding of the HCUs i.e. their characteristics and patterns ofhealthcare utilization can help the country to identify potential HCUs and developmore tailored and appropriate interventions to improve the management and financing of these patients.

Even though the Thai government investsapproximately 13.3% of the government budget on public health which cover almost 100% of its 70 million population,[13]the Universal Coverage Scheme (UCS) budget keeps increasing every year during the past decadeand the UCS budget is increasing at a faster rate than the annualgross domestic product (GDP) growth in the past few years which is directly affecting the sustainability of the healthcare system.[13 14]The three main health insurance programs employed to cover nearly allThai population includethe Civil Servant Medical Benefit Scheme (CSMBS) for government employees and their dependents, the Social Security Scheme (SSS) for formal private sector employees, and the UCS for the remaining of Thai citizens.[13]The UCS,which is managed by National Health Security Office (NHSO),covers approximately 75% ofthe total population who are not eligible for the CSMBS and SSS, including low-income or unemployed citizens. Characteristics of three health insurance programs in Thailand are published elsewhere.[13]

The UCS provides a comprehensive benefits package for curative, rehabilitation as well as health promotion and prevention services.[15]Most of the UCS budget is allocated to outpatient and inpatient services. Payment for outpatientcare generally is based on capitation while inpatientpaymentis using diagnosis-related groups (DRGs).[16 17]In addition, there is an additional budget for specific high-costinterventions or medications.[18]Nearly half of the UCS expenditure (e.g.,42% in 2011) is spent on inpatient care.[14]

For peer review only - http://bmjopen.bmj.com/site/about/guidelines.xhtml

Findings from a study on the HCUs phenomenon underThailand’s biggest public health insurance scheme, i.e.,UCS,is essential for developingpotential measures that are deemed‘preventable’ spending. This study may be the first study in low- and middle-income countries(LMICs) and its findings can be used to compare with the previous studies conducted in high-income countries andto shed light for healthcare managers and policy-makers in other LMICs.This study aimed to answer the followingquestions. First, who were the high-cost users? Second, how significant is the spending among HCUs as compared to total inpatient care budget? Third, what werethe common characteristics andpatterns ofhealth care utilization and expenditure among high-cost users compared to low-cost users?Lastly, what were potential predictors of high-cost users, i.e., what characteristics increased the chance of becoming HCUs?

METHODS

Study population, setting, and data

This studywas a retrospective secondary data analysis whichexaminedinpatient department (or hospitalization)datafromThailand’s UCSobtained from NHSOovera 5-year period from 2014 to 2018.Using this administrative hospitalization database, our study population included Thai citizens (of any ages) who were hospitalized and discharged under UCS between October 1st, 2014, and September 30th, 2019, according to the Thai government’s fiscal year.

All data were anonymized and de-identified. Records that were duplicated or had incomplete data were excluded from analysis. To ensure that the data wereaccurate and consistent, conflicting demographic information about date of birth, gender, and admit and discharge dates were investigated and resolved. Conflictingrecords were removed.

Variables in the analysis comprised of:gender; date of birth; primary diagnosis (using ICD-10 codes);admit and discharge dates; discharge status (full recovery, improved, not improved, normal delivery, undelivered, normal child discharged with mother, separation of mother and baby, stillbirth, and death); types of hospitals (i.e., from the lowest level being clinics,community hospitals to the higher level namely, private hospitals, general hospitals, regional hospitals,and other hospitals not under the Ministry of Public Health such as university hospitals); health regionsas shown in the Supplementary Appendix (**Figure A1**);Charlson comorbidity index; and reimbursement cost (which refers to the amount estimated to be paid by NHSO and reported in the hospitalization database).

The study received ethics approval from Institute for the Development of Human Research Protections, Ministry of Public Health, Thailand.

For peer review only - http://bmjopen.bmj.com/site/about/guidelines.xhtml

Analysis

Using the NHSO's hospitalizationdatabase described earlier, we examined HCUs and persistent HCUs. HCUs and LCUs were defined by the percentile of hospitalization costs accrued. HCUswere individuals who had cost higher than 95% of all users (>95th percentile or the top 5%). LCUs were patients in the lowest cost group (< 50th percentile of cost or the bottom 50%). The dataset was treated as repeated cross-sectional when examining HCUs;and the analysis was conducted separately by year. We defined persistent HCUs as those who were identified as HCUs for more than one year; further explanation is provided below. When examining persistent HCUs, we constructed a panel dataset by tracking the individual status (of being an HCU) over years.

We tracked every hospital admissionwithin each fiscal year (October 1st to September 30th). To address the study aims, first, we sought to determine the concentration of inpatient department expenditures to explore the phenomenon of HCUs in Thailand. Total costs associated with all visits within each fiscal year for each patient (i.e., estimated reimbursement costs)were calculated and then individuals were grouped into 5 groups according to percentile of costs: 1) above the 99th percentile; 2) between the 95th and the 98th percentile; 3) between the 90th and 94th percentiles; 4) between the 50th and 89th percentiles; and 5) below the 50th percentile.

Next, we examined the differences in demographiccharacteristics, costs, and comorbidities of HCUs compared to LCUs.We analyzed descriptive characteristics of each group by age and gender,and identified main diagnosis based on ICD-10 codes. We also reported health care utilization and expenditure patterns between HCUs and LCUs, specifically number of visits, length of stay (LOS), types of hospitals visited, and annual cost per patient.

Logistic regression was conducted to identify predictors of HCUs. The outcome variable was whether a patient was an HCU(yes/no) and the exploratory variables included age, gender, number of visits, types and health regions of hospitals visited, main diagnoses, and comorbidities (measured as Charlson comorbidity index, and the presence of key primary diagnosis namely neoplasms, circulatory disease, respiratory disease, digestive problems, and injuries).Interaction terms werealso included to examine the heterogeneous effects on becoming a potential HCU, specifically between death, age, or gender and the top five primary diagnoses (neoplasms, circulatory disease, respiratory disease, digestive problems, and injuries) including between types of hospital and health regions.

To identify the persistent HCUs, only the data between 2015 and 2017 were examined due to the completeness of the data. To construct a panel dataset, patients who were presented in the dataset in 2015 were used for the analysis. We dropped the patients who died in 2015.

For peer review only - http://bmjopen.bmj.com/site/about/guidelines.xhtml

For the remaining patients, we examined their HCUstatus in year 2016 and 2017 to determine whether they were persistent HCUs.They were grouped into threelevels: 0 (not an HCU for all the three years); 1 (being an HCU for only one year); and 2 (being an HCU for at leasttwo years). Group 2 was defined as persistent HCUs. Furthermore, a multinomial logistic regression was applied to examine the predictors for persistent HCUs.

All analyseswere conducted using Stata Statistical Software (Release 15, College Station, TX).Chi-square tests and t-tests were used to test forsignificant differences.

RESULTS

A total of29,899,719 admissions (14,464,180 unique patients)between fiscal years 2014–2018 were analyzed. On average, each year had approximately 5.9 million admissions (about 3.9 million unique patients).

The hospitalization data of UCS have shown that, comparable to existing literature on HCUs phenomenon, a small proportion of patients (the top 5% of hospitalized population or HCUs) consumed almost half of healthcare expenditure. The same trend was observed over a 5-year period as presented in **Figure 1**.During this period, patients in thetop 1% and 5% cost groups accounted for16-18% and 45-48% of total spending, respectively. Furthermore, a separate analysis showed that HCUs had higher annual mortality rates compared to LCUs. Among HCUs, themortality ranged from 19% to 21% over 5 years, compared with0.1% to 1.3% in LCUs.

Figure 1Distribution of total hospitalization's expenditures across cost percentiles

To facilitate the understanding of HCU phenomenon, the profile of HCUs were studied. **Table1**provides information on HCUs compared with LCUs.

For peer review only

For peer review only - http://bmjopen.bmj.com/site/about/guidelines.xhtml

Table 1 Demographic characteristics and patterns of utilization and expenditure of HCUs and LCUs from 2014 to 2018*

Characteristic	2014		2015		2016		2017		2018	
	HCUs	LCUs	HCUs	LCUs	HCUs	LCUs	HCUs	LCUs	HCUs	LCUs
Number of patients	234,214	1,913,712	235,954	1,964,952	243,850	1,991,451	239,180	1,953,317	243,951	1,992,271
Number of admissions	864,787	1,991,875	863,308	2,054,265	899,142	2,090,249	926,434	2,053,590	951,527	2,096,159
Mean age in years (SD)	54.6 (22.6)	22.6 (25.0)	54.5 (22.4)	24.1 (25.5)	55.4 (22.3)	23.8 (25.4)	55.6 (22.3)	24.8 (25.9)	56.1 (22.1)	25.2 (26.1)
Male (%)	55%	44%	55%	44%	55%	45%	55%	45%	55%	45%
Average length of stay in days (SD)	11.3 (21.2)	2.5 (2.8)	11.3 (22.0)	2.6 (2.1)	10.9 (21.6)	2.6 (2.9)	10.8 (19.1)	2.6 (2.6)	10.4 (18.8)	2.6 (2.3)
Average number of visits (SD)	3.7 (3.3)	1.0 (0.2)	3.7 (3.3)	1.0 (0.2)	3.7 (3.4)	1.0 (0.2)	3.9 (3.5)	1.0 (0.2)	3.9 (3.5)	1.0 (0.2)
Number of hospitals visited (% of admissions)										
1	49.4%	99.0%	49.7%	99.0%	49.8%	99.0%	48.5%	99.0%	48.4%	99.0%
2	41.3%	1.0%	40.4%	1.0%	40.7%	1.0%	41.6%	1.0%	41.8%	1.0%
3	8.1%	-	8.6%	-	8.3%	-	8.7%	-	8.6%	-
>3	1.2%	-	1.3%	-	1.2%	-	1.2%	-	1.2%	-
Types of hospitals visited (% of admissions)										
Regional hospitals	35.5%	16.2%	33.6%	16.2%	34.9%	15.8%	34.0%	16.3%	34.3%	16.0%
General hospitals	21.2%	21.8%	20.0%	22.0%	20.9%	21.7%	21.5%	22.0%	22.4%	21.8%
Community hospitals	20.1%	53.3%	18.7%	54.2%	19.6%	54.1%	20.7%	53.3%	21.4%	53.7%
Others	23.2%	8.7%	27.7%	7.6%	24.6%	8.4%	23.8%	8.4%	21.9%	8.5%
Average total cost per	130,700	2,800	107,600	2,300	121,300	2,600 (970)	123,800	2,700	127,700	2,900

For peer review only - http://bmjopen.bmj.com/site/about/guidelines.xhtml

Characteristic	2014		2015		2016		2017		2018	
	**HCUs**	**LCUs**	**HCUs**	**LCUs**	**HCUs**	**LCUs**	**HCUs**	**LCUs**	**HCUs**	**LCUs**
patient in baht (SD)	(102,500)	(1,070)	(107,600)	(820)	(88,500)		(87,100)	(1,060)	(88,800)	(1,130)

HCUs = high-cost users; LCU = low cost users; SD = standard deviation.

*The number (except number of patients and admissions) presented in table was rounded to the nearest whole number.

For peer review only - http://bmjopen.bmj.com/site/about/guidelines.xhtml

Over the 5-year period,HCUs were olderand included more mencompared to LCUs. The mean age ranged between 55-56 years and 23-25 years among HCUs and LCUs, respectively. HCUshad a longer length of stayand used more health care services (higher frequency of hospital visits and higher number of visited hospitals). Moreover, HCUs visitedhigher level hospitals (e.g., regional and university hospitals), while LCUs visited community hospitals. The pattern of the results were similar across the 5-years period.

As for the expenditure pattern, HCUs spent considerably more per year than LCUs inthe index year. Over 5 years, the average annual spending for HCUs was between 107,600 (± 107,600) to 130,700 (± 102,500) Baht, compared to 2,300 (± 820) to 2,900 (± 1,130) Baht for LCUs, where 1 US dollar is approximately 32 Thai baht. Details of an expenditure pattern are presented in **Table1**.

Themostcommon and most expensive clinical conditions between HCUs and LCUs were examined and found to differ.**Table 2** confirms that the expenditures were spent on different diagnoses. Among HCUs, the most frequent diagnoses included neoplasms, diseases of circulatory system and respiratory system which consumed more than half of annual expenditure. Among LCUs, the most frequent diagnoses were broaderand included infectious and parasitic diseases, factors influencing health status and contact with health services (e.g., admissions for investigation), and diseases of the respiratory system;almost half of LCUs' annual expenditure was spent on these conditions.

For peer review only

For peer review only - http://bmjopen.bmj.com/site/about/guidelines.xhtml

1 **Table 2 The top five primary diagnoses of HCUs and LCUs over a 5-year study period***

High-cost users (HCUs)			Low cost users (LCUs)		
**Conditions**	**% of total admissions**	**% of total expenditures**	**Conditions**	**% of total admissions**	**% of total expenditures**
Neoplasm	22-23	17-20	Certain infectious and parasitic diseases	17-19	15-17
Diseases of circulatory system	17-20	25-30	Factors influencing health status and contact with health services	14-17	9-12
Diseases of respiratory system	13-14	10-12	Diseases of the respiratory system	11-14	13-17
Diseases of genitourinary system	7-8	3	Pregnancy, childbirth, puerperium	10-12	11-14
Diseases of the digestive system	7	5	Injury, poisoning and certain other consequences of external causes	9	8-9

2 HCUs = high-cost users; LCU = low cost users.

3 *The findings reported covered the 5-year study period so the lowest percentage and highest percentage during this period were presented.

For peer review only

Regression models were used to examinethe potential predictors of being an HCU (**Table 3**). The findings suggested that age, gender, types and zones of hospital admissions, disease and multimorbidity, number of visits, and mortality were associated with becoming an HCU (although the relative contribution of these factors towards the expenditure varied).Most of the interaction terms were statistically significant,suggesting that the impact of these factors were heterogenous among different groups.

For peer review only

For peer review only - http://bmjopen.bmj.com/site/about/guidelines.xhtml

Table 3 Predictors associated with high-cost users in UCS*

Factors Predicting Being High-cost User	2014	2015	2016	2017	2018
	OR (95%CI)	OR (95%CI)	OR (95%CI)	OR (95%CI)	OR (95%CI)
Age	1.03 (1.03, 1.03)	1.03 (1.03, 1.04)	1.03 (1.03, 1.04)	1.03 (1.03, 1.03)	1.03 (1.03, 1.03)
Age*Age	0.99 (0.99, 0.99)	0.99 (0.99, 0.99)	0.99 (0.99, 0.99)	0.99 (0.99, 0.99)	0.99 (0.99, 0.99)
Gender (ref: female)	0.73 (0.71, 0.74)	0.77 (0.75, 0.78)	0.79 (0.78, 0.80)	0.79 (0.78, 0.80)	0.82 (0.81, 0.83)
Hospital type (ref: other clinics)					
Regional hospitals	3.42 (3.37, 3.47)	3.45 (3.40, 3.50)	3.47 (3.42, 3.52)	3.24 (3.19, 3.29)	3.16 (3.11, 3.20)
General hospitals, community hospitals, and other MOPH agencies	1.01 (1.00, 1.03)	1.03 (1.02, 1.05)	1.00 (0.98, 1.02)	1.02 (1.00, 1.03)	1.02 (1.01, 1.04)
Non MOPH agencies such as university hospitals	6.60 (6.45, 6.75)	9.06 (8.86, 9.26)	7.51 (7.35, 7.69)	7.21 (7.04, 7.37)	6.42 (6.28, 6.57)
Private hospitals	2.10 (2.03, 2.18)	4.06 (3.94, 4.18)	3.46 (3.35, 3.56)	3.28 (3.17, 3.40)	3.15 (3.04, 3.26)
Hospital zone: (ref: hospitals outside Bangkok)					
Bangkok	3.12 (2.97, 3.29)	4.73 (4.51, 4.97)	3.8 (3.61, 3.99)	3.92 (3.73, 4.12)	3.25 (3.09, 3.42)
Primary diagnosis					
Neoplasm	9.30 (8.78, 9.85)	8.96 (8.46, 9.49)	9.09 (8.59, 9.62)	8.77 (8.28, 9.28)	10.0 (9.49, 10.6)
Diseases of circulatory system	9.48 (9.01, 9.97)	9.6 (9.13, 10.0)	10.5 (10.0, 11.1)	11.0 (10.4, 11.5)	10.9 (10.4, 11.5)
Diseases of respiratory system	1.01 (0.98, 1.05)	1.00 (0.96, 1.03)	1.04 (1, 1.07)	0.99 (0.96, 1.02)	1.11 (1.07, 1.15)
Diseases of the digestive system	0.62 (0.59, 0.65)	0.61 (0.58, 0.65)	0.62 (0.59, 0.65)	0.62 (0.59, 0.65)	0.61 (0.58, 0.64)
Injury, or poisoning and certain other consequences of external causes	2.41 (2.33, 2.50)	2.45 (2.37, 2.54)	2.37 (2.29, 2.46)	2.1 (2.02, 2.18)	2.26 (2.17, 2.34)
Comorbidity (Charlson comorbidity index)	1.07 (1.07, 1.08)	1.05 (1.04, 1.05)	1.05 (1.04, 1.05)	1.03 (1.03, 1.04)	1.02 (1.01, 1.02)
Death	6.53 (6.34, 6.73)	5.42 (5.26, 5.58)	5.43 (5.28, 5.59)	4.73 (4.59, 4.88)	4.06 (3.94, 4.19)
Number of primary diagnoses	1.20 (1.19, 1.21)	1.25 (1.24, 1.26)	1.24 (1.23, 1.25)	1.27 (1.26, 1.28)	1.28 (1.27, 1.29)

For peer review only

For peer review only - http://bmjopen.bmj.com/site/about/guidelines.xhtml

Factors Predicting Being High-cost User	2014	2015	2016	2017	2018
	OR (95%CI)	OR (95%CI)	OR (95%CI)	OR (95%CI)	OR (95%CI)
Number of visits in a year	1.65 (1.64, 1.66)	1.57 (1.56, 1.58)	1.55 (1.54, 1.56)	1.56 (1.56, 1.57)	1.54 (1.54, 1.55)
Neoplasm*Death	0.31 (0.3, 0.33)	0.38 (0.36, 0.39)	0.39 (0.37, 0.41)	0.39 (0.38, 0.41)	0.43 (0.41, 0.45)
Diseases of circulatory system*Death	0.36 (0.35, 0.37)	0.40 (0.39, 0.42)	0.35 (0.33, 0.36)	0.39 (0.38, 0.41)	0.44 (0.42, 0.46)
Diseases of respiratory system*Death	2.00 (1.92, 2.08)	1.59 (1.53, 1.66)	1.75 (1.68, 1.81)	1.50 (1.45, 1.56)	1.44 (1.39, 1.50)
Diseases of the digestive system*Death	1.09 (1.04, 1.14)	1.2 (1.15, 1.26)	1.12 (1.07, 1.18)	1.22 (1.16, 1.28)	1.38 (1.31, 1.44)
Injury, or poisoning and certain other consequences of external causes*Death	0.75 (0.71, 0.79)	0.64 (0.6, 0.67)	0.63 (0.59, 0.66)	0.71 (0.67, 0.75)	0.76 (0.72, 0.8)
Neoplasm*Age	0.99 (0.99, 0.99)	0.99 (0.99, 0.99)	0.99 (0.99, 0.99)	0.99 (0.99, 0.99)	0.99 (0.98, 0.99)
Diseases of circulatory system*Age	0.98 (0.98, 0.98)	0.98 (0.98, 0.98)	0.98 (0.98, 0.98)	0.98 (0.98, 0.98)	0.98 (0.98, 0.98)
Diseases of respiratory system*Age	1.01 (1.01, 1.01)	1.00 (1.00, 1.00)	1.00 (1.00, 1.01)	1.00 (1.00, 1.00)	1.00 (1.00, 1.00)
Diseases of the digestive system*Age	1.01 (1.01, 1.01)	1.00 (1.00, 1.01)	1.01 (1.00, 1.01)	1.00 (1.00, 1.01)	1.00 (1.00, 1.01)
Injury, or poisoning and certain other consequences of external causes*Age	0.99 (0.99, 1.00)	1.00 (1.00, 1.00)	1.00 (1.00, 1.00)	1.00 (1.00, 1.00)	1.00 (0.99, 1.00)
Neoplasm*Gender	0.84 (0.82, 0.87)	0.75 (0.72, 0.77)	0.71 (0.69, 0.74)	0.71 (0.69, 0.73)	0.66 (0.64, 0.68)
Diseases of circulatory system*Gender	1.00 (0.98, 1.03)	0.9 (0.88, 0.92)	0.91 (0.89, 0.93)	0.9 (0.88, 0.92)	0.88 (0.86, 0.91)
Diseases of respiratory system*Gender	0.98 (0.95, 1.01)	0.98 (0.95, 1.01)	0.94 (0.91, 0.96)	0.93 (0.91, 0.96)	0.91 (0.89, 0.93)
Diseases of the digestive system*Gender	1.07 (1.04, 1.11)	1.06 (1.03, 1.1)	1.02 (0.99, 1.05)	1.03 (1, 1.07)	1.01 (0.98, 1.05)
Injury, or poisoning and certain other consequences of external causes*Gender	1.17 (1.13, 1.21)	1.2 (1.16, 1.24)	1.21 (1.18, 1.25)	1.23 (1.19, 1.26)	1.22 (1.19, 1.26)
Hospital zone: Bangkok* Regional hospitals	0.62 (0.59, 0.66)	0.57 (0.54, 0.61)	0.59 (0.56, 0.63)	0.59 (0.56, 0.63)	0.66 (0.62, 0.69)
Hospital zone: Bangkok* General hospitals, community hospitals, and other MOPH agencies	1.79 (1.71, 1.88)	1.88 (1.79, 1.96)	1.83 (1.75, 1.92)	1.69 (1.61, 1.76)	1.7 (1.62, 1.78)
Hospital zone: Bangkok* Non MOPH agencies	0.30 (0.29, 0.32)	0.23 (0.22, 0.24)	0.26 (0.24, 0.27)	0.25 (0.24, 0.27)	0.3 (0.29, 0.32)

For peer review only - http://bmjopen.bmj.com/site/about/guidelines.xhtml

Factors Predicting Being High-cost User	2014	2015	2016	2017	2018
	OR (95%CI)	OR (95%CI)	OR (95%CI)	OR (95%CI)	OR (95%CI)
Hospital zone: Bangkok* Private hospitals	0.54 (0.51, 0.58)	0.28 (0.27, 0.30)	0.34 (0.32, 0.36)	0.33 (0.31, 0.35)	0.32 (0.3, 0.34)
Constant	0.00 (0.00, 0.00)	0.00 (0.00, 0.00)	0.00 (0.00, 0.00)	0.00 (0.00, 0.00)	0.00 (0.00, 0.00)

95% CI = 95% confidence interval; OR = odds ratio.

*All variables are significant (p-value < 0.005) across 5 years.

For peer review only

For peer review only - http://bmjopen.bmj.com/site/about/guidelines.xhtml

On average, the high-cost users were more likely to be: 1) older; 2) male; 3) admitted at higher level hospitals (e.g., regional and university hospitals); 4) admitted at hospitals in Bangkok; 5) patient with a primary malignant tumor, diseases of the circulatory system, injury, poisoning and certain other consequences of external causes; 6) patients with multiple diagnosis; 7) patients with other comorbidities; 8) patients with more than one hospitalization within a year; and 9) patients who died at discharged. The marginal effects are presented inthe Supplementary Appendix (**Table A2**), showing thatthe results,in term of the probability of being an HCU for averageindividuals,were in line with the logistic regression. For example, in 2014, an average increasein age by one year had a predicted 0.03% higherchance of becomingan HCU. The average patient with neoplasm (a primary malignant tumor) had a predicted 8% higher chance of being an HCU.

The interactions of each of thepredictors with the high-cost user status showed that individuals who had diseases of the respiratory and/or digestive system and died at discharge were more likely to be HCUs.Moreover, those who admitted at a general hospital, community hospital, or other MOPH agencies in Bangkok were also more likely to be HCUs.

This study alsoexamined factors that could potentially identify who waslikely to remain an HCU over time. In the data, all the persistent HCUs were HCUs for at least two years. No patient was found to be an HCU for three consecutive years. The conditions that were associated with a higher likelihood of becoming persistently HCUswere similar to the predictors of HCUs as shown in the Supplementary Appendix (**Table A3**).However, the magnitude of the impact could differ in some cases. For example, the relative risk ratio(RRR) for neoplasms was 7.9 for HCUs, but was 43.5 for persistent HCUs. This finding suggests that patientswith neoplasms were likely to be HCUs, and HCUs with neoplasms were also likely to be persistent HCUs.

DISCUSSION

There is a need for understanding the characteristics of HCUs who are in highest need in terms of consuming health care resources and their underlying factors that may be driving costs. This study confirmed that the HCUs phenomenon existed in Thailand, an upper middle-income country,where a majority of inpatient care spending under is concentrated in the top 5% of hospitalized population.

The predictors of HCUs appear to align with those reported in the international literature. Older patients and males were more likely to be HCUs than younger and female patients.[10] HCUs were also likely to have multiple diagnoses, comorbidity conditions, and serious types of diagnoses (e.g., cancer and cardiovascular diseases); and therefore, they were likely to

For peer review only - http://bmjopen.bmj.com/site/about/guidelines.xhtml

also be admitted to hospitals in Bangkok (the capital of Thailand) with more advanced treatments available. Furthermore, actual costs of health care resources consumed by these hospitals should be explored to ensure that the expense paid by NHSO using diagnosis-related group approach is adequate in order to develop and maintain referral systems, and prevent hospital rejecting referred patients.Knowing the common diagnoses of HCUs could also underline the associated costs or burden of these health conditions. As frequent users of the health care system, HCUs would be expected to visit a hospital more than once a year as the findings have shown. Information on these potential predictors of HCUs could assist health care professionals and hospitals in creating more tailored treatment and management plan for patients who were likely to be HCUs. On the other hand, patients who died at discharged were also determined to have higher chance to be HCUs. This predictor (died at discharged) may not be as informative in assisting the development of treatment plan as it is related to the outcome. In the model without this predictor, other predictors (mentioned above) remain to be significant in helping to identify potential HCUs.

The study highlighted strengths and several limitations for future research. To our knowledge, this study is the first study to confirm this unique phenomenon in a LMIC and could be applicable to other settings because knowing who are in need would equip the health care system to respond accordingly, for example, health care providers could design more tailored and appropriate care to the patients. This research represents an example of how existing administrative database could be used to answer policy-relevant questions. The study highlighted the importance of data quality in administrative databases. There were issues regarding the completeness of data between 2014 and 2018. Year 2014 was the first year that data were computerized; therefore, the data were not complete as the data entering was just started. Claims data from hospitals are usually collected by hospitals through medical charts (which can take time) and entered into NHSO system on a later date (after being checked for accuracy through NHSO auditing step) so the data in 2018arenot completely integrated. Regardless, it is useful to include data from 2014 and 2018 to assist the study of magnitude and trend of HCUs.Moreover, the analyses encountered several situations such as conflicting sex or date of birth of same individuals with multiple hospitalizations. For instance, someone with multiple visits could have different sex for each visit. Furthermore, when linking across databases (hospitalization and death databases), a data quality check should be put in place to ensure accurate mergingof data; this step should be standard practice for similar type of analysis. As a secondary data analysis, the study was limited by data availability.Consequently, there could be additional potential confounders which could not be included due to lack of data (e.g., education and income), an area where future research could build on. For example, if we were able to link the

For peer review only - http://bmjopen.bmj.com/site/about/guidelines.xhtml

database to outpatient visit database, the analysis may be able to examine potential predictors of HCUs at ambulatory care stage as well including for rehabilitation, and prevention and promotion programs.In addition, selected high-cost specialty medicines and interventions (such as 'high-cost medicines E2 access program',[18] stent and prosthetic hip) were not included in the analysis.These interventions areprocured centrally by NHSO anddistributed directly to hospitals.Therefore, they are not included in the claims data and could be explored in future research.

The findings could be applied to several scenarios to support policy-making processwherefuture research could examine further. For example, knowing who the HCUs are could help health professional monitor the magnitude and trend of HCUs and take into account the HCU phenomenon when developing relevant policies such as UCS reimbursement policy in order to reduce avoidable HCU or improve outcome from high-cost interventions.Moreover, an algorithm (a mechanism) could be built to support tertiary hospitals to identify HCUs upon admission and develop measures to support them on a routine basis as opposed to ad hoc monitoring and to consider whether to provide aggressive treatment to patients with low probability of survival (e.g., higher Charlson comorbidity score or with many organ failure) especially in palliative care management. Furthermore, public communications should be made to raise awareness and encourage prevention of riskfactors through prevention & promotion (P&P) program to avoid having comorbidities or other chronic diseases (e.g., cancer).Additionally, in the healthcare system without UHC, government could explore having people with risk factors of HCUs to be one of the priority groups so they can monitor closely and ensure that the patients are receiving appropriate care before they become HCUs.Low-value care is also of concern in healthcare systems,[3 4 19] and a study of low-value care usage among HCUs could explore the significance of these low-value care services being used by this population. Further work on referral system across different level of health facilities and other types of services (in addition to hospitalization) including how to provide right care at the right time and right place could create evidence to assist policy-makers in their planning and delivery of health services. Exploration of HCUs and their transition across years could further develop our understanding of those with expensive necessity.

For peer review only

For peer review only - http://bmjopen.bmj.com/site/about/guidelines.xhtml

Acknowledgements

The authors would like to thank the National Health Security Office (including Ms. Kanjana Sirigomon, Ms. Jutatip Thungthong, and Mr. Poonchana Wareechi) for providing the data and support for this study; Dr. Lou Jing, a research fellow at Health Intervention and Policy Evaluation Research, Saw Swee Hock School of Public Health, National University of Singapore, for providing support for the data cleaning steps; and Mr. Sarin KC from HITAP for proofreading support.

Contributors

The study was conceptualised by YT and WI. WR, WY, RB, NK, and JT contributed to its design. Fundingwas acquired by YT. JTwas instrumental in providing data access.The analysesand interpretationwere carried out by WR, WY, RB, undersupervision of YT and WI. NK provided assistance with presentation of thefindings.The initial draft was written by WR and WI, and refined with input from WY, RB, NK, JT and YT. All authors read and approved the final manuscript.

Funding

This study was supported by the Thailand Research Fund under the senior research scholar on Health Technology Assessment [grant numbers RTA5980011] and Ministry of Public Health, Thailand. Health Intervention and Technology Assessment Programme (HITAP) and Saw Swee Hock School of Public Health (SSHSPH) are part of the International Decision Support Initiative (https://www.idsihealth.org/), which supports countries to get the best value for money from health spending. International Decision Support Initiative receives funding support from the Bill & Melinda Gates Foundation (OPP1202541), the UK Department for International Development, and the Rockefeller Foundation.

Disclaimer

The findings, interpretations and conclusions expressed in this article are of the authors and do not necessarily reflect the views of the aforementioned funding agencies.

Competing interests

The authors declared no competing interests relevant to the submitted manuscript.

Patient and Public Involvement

No patient involved.

For peer review only - http://bmjopen.bmj.com/site/about/guidelines.xhtml

Patient consent for publication

This study has used an existing administrative database (with de-identified individual-levelhospitalization data); therefore, patients had no direct involvement and no consent was required.

Ethics approval

The research was approved byInstitute for the Development of Human Research Protections (IHRP No.795/2561, Date of approval on 22 November 2018) and complied with the Non-disclosure agreement between National Health Security Office and Health Intervention and Technology Assessment Program to protect individual identification.

Data sharing statement

The data are not publicly available due to privacy or ethical restrictions according to the data sharing agreement with the National Health Security Office.

For peer review only

For peer review only - http://bmjopen.bmj.com/site/about/guidelines.xhtml

REFERENCES

1. Watkins DA, Jamison DT, Mills A, et al. Universal health coverage and essential packages of care. In: Jamison DT, Gelband H, Horton S, eds. Disease control priorities: improving health and reducing poverty. 3 ed. Washington (DC): The International Bank for Reconstruction and Development 2017:43-68.
2. Teerawattananon Y, Kingkaew P, Koopitakkajorn T, et al. Development of a health screening package under the universal health coverage: the role of health technology assessment. *Health Econ* 2016;25(Suppl 1):162-78. doi: 10.1002/hec.3301 [published Online First: 2016/01/17]
3. Badgery-Parker T, Pearson SA, Chalmers K, et al. Low-value care in Australian public hospitals: prevalence and trends over time. *BMJ Qual Saf* 2019;28(3):205-14. doi: 10.1136/bmjqs-2018-008338 [published Online First: 2018/08/08]
4. Schwartz AL, Landon BE, Elshaug AG, et al. Measuring low-value care in Medicare. *JAMA Intern Med* 2014;174(7):1067-76. doi: 10.1001/jamainternmed.2014.1541 [published Online First: 2014/05/14]
5. Breslow L. From disease prevention to health promotion. *JAMA* 1999;281(11):1030-33. doi: 10.1001/jama.281.11.1030 [published Online First: 1999/03/23]
6. Labarthe DR, Dunbar SB. Global cardiovascular health promotion and disease prevention: 2011 and beyond. *Circulation* 2012;125(21):2667-76. doi: 10.1161/circulationaha.111.087726 [published Online First: 2012/05/31]
7. Calver J, Brameld KJ, Preen DB, et al. High-cost users of hospital beds in Western Australia: a population-based record linkage study. *The Medical journal of Australia* 2006;184(8):393-7. [published Online First: 2006/04/19]
8. Kozyrskyj A, Lix L, Dahl M, et al. High-cost users of pharmaceuticals: Who are they? Manitoba: Manitoba Centre for Health Policy, Department of Community Health Sciences, Faculty of Medicine, University of Manitoba 2005:1-53.
9. Rais S, Nazerian A, Ardal S, et al. High-cost users of Ontario's healthcare services. *Healthcare policy = Politiques de sante* 2013;9(1):44-51. [published Online First: 2013/08/24]
10. Rosella LC, Fitzpatrick T, Wodchis WP, et al. High-cost health care users in Ontario, Canada: demographic, socio-economic, and health status characteristics. *BMC Health Serv Res* 2014;14(1):532. doi: 10.1186/s12913-014-0532-2 [published Online First: 2014/11/02]
11. Tanke MAC, Feyman Y, Bernal-Delgado E, et al. A challenge to all. A primer on inter-country differences of high-need, high-cost patients. *PloS one* 2019;14(6):e0217353. doi: 10.1371/journal.pone.0217353 [published Online First: 2019/06/20]
12. Wodchis WP, Austin PC, Henry DA. A 3-year study of high-cost users of health care. *CMAJ : Canadian Medical Association journal = journal de l'Association medicale canadienne* 2016;188(3):182-88. doi: 10.1503/cmaj.150064 [published Online First: 2016/01/13]
13. Tangcharoensathien V, Witthayapipopsakul W, Panichkriangkrai W, et al. Health systems development in Thailand: a solid platform for successful implementation of universal health coverage. *Lancet* 2018;391(10126):1205-23. doi: 10.1016/s0140-6736(18)30198-3 [published Online First: 2018/02/06]
14. Hanvoravongchai P. Health financing reform in Thailand: toward universal coverage under fiscal constraints. UNICO Study Series;No. 20., 2013.
15. Mohara A, Youngkong S, Velasco RP, et al. Using health technology assessment for informing coverage decisions in Thailand. *J Comp Eff Res* 2012;1(2):137-46. doi: 10.2217/cer.12.10 [published Online First: 2012/03/01]
16. Pannarunothai S. Diagnosis Related Groups (DRGs) Development in Thailand. In: Pramualratana P, Wibulpolpraser S, eds. Health Insurance Systems in Thailand. Nonthaburi: Health Systems Research Institute (HSRI) 2002:143-54.

For peer review only - http://bmjopen.bmj.com/site/about/guidelines.xhtml

17. Pongpirul K, Walker DG, Winch PJ, et al. A qualitative study of DRG coding practice in hospitals under the Thai Universal Coverage Scheme. *BMC Health Serv Res* 2011;11(1):71. doi: 10.1186/1472-6963-11-71
18. Sruamsiri R, Wagner AK, Ross-Degnan D, et al. Expanding access to high-cost medicines through the E2 access program in Thailand: effects on utilisation, health outcomes and cost using an interrupted time-series analysis. *BMJ Open* 2016;6(3):e008671. doi: 10.1136/bmjopen-2015-008671 [published Online First: 2016/03/19]
19. Bhatia RS, Levinson W, Shortt S, et al. Measuring the effect of Choosing Wisely: an integrated framework to assess campaign impact on low-value care. *BMJ quality & safety* 2015;24(8):523-31. doi: 10.1136/bmjqs-2015-004070 [published Online First: 2015/06/21]

For peer review only

For peer review only - http://bmjopen.bmj.com/site/about/guidelines.xhtml

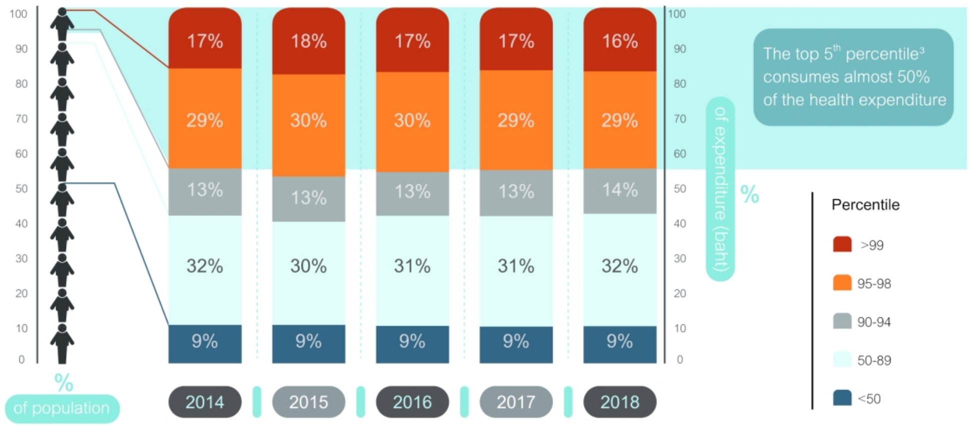

Distribution of total hospitalization's expenditures across cost percentiles

Figure A1 Different health regions in Thailand

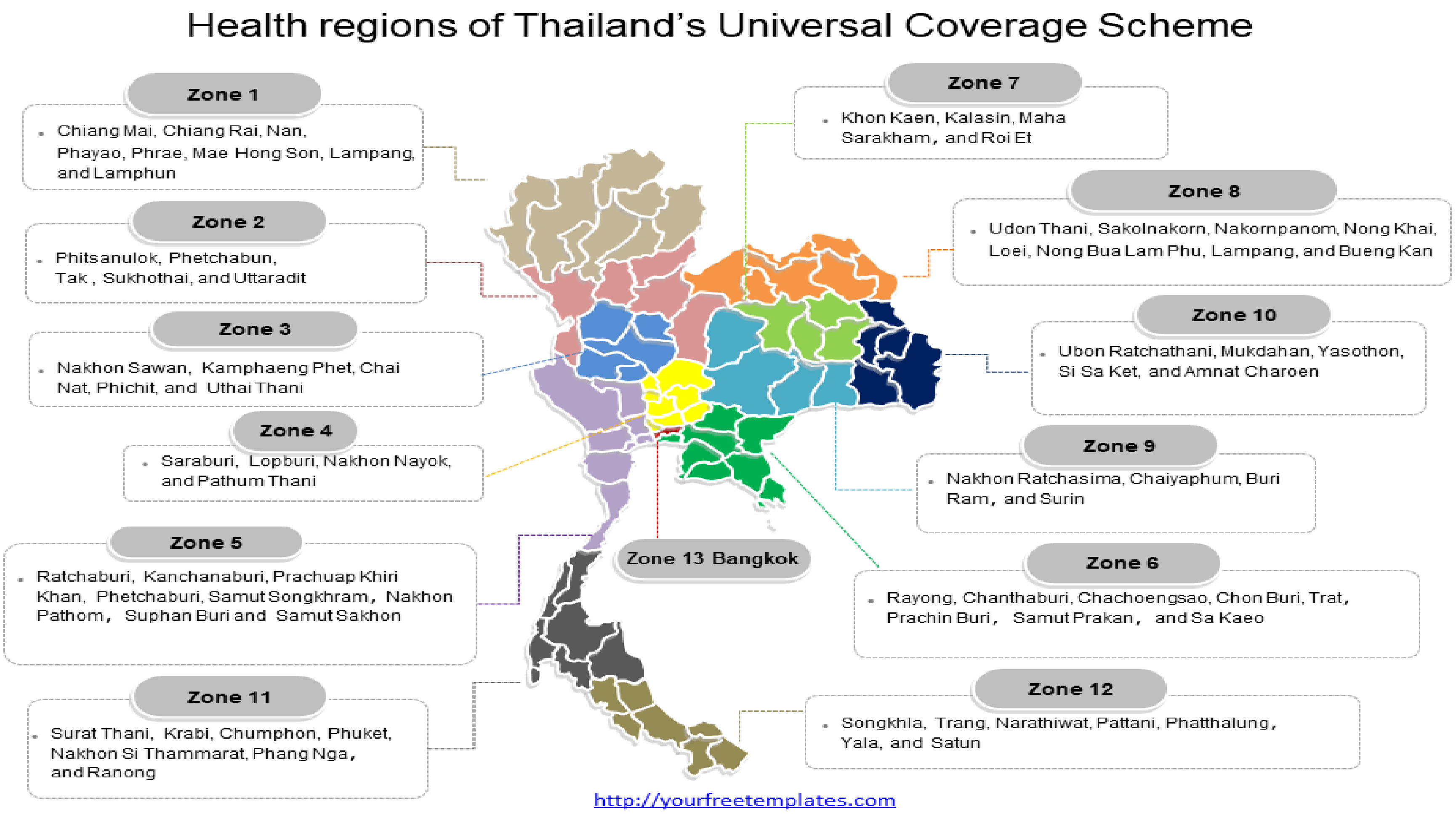

Table A2 Marginal effect of Predictors associated with high-cost users in UCS

	2013	2014	2015	2016	2017
	Prob (95%CI)	Prob (95%CI)	Prob (95%CI)	Prob (95%CI)	Prob (95%CI)
Age	0.00 (0.00, 0.00)	0.00 (0.00, 0.00)	0.00 (0.00, 0.00)	0.00 (0.00, 0.00)	0.00 (0.00, 0.00)
Gender (ref: female)	-0.01 (-0.01, -0.01)	-0.01 (-0.01, -0.01)	-0.01 (-0.01, -0.01)	-0.01 (-0.01, -0.01)	-0.01 (-0.01, -0.00)
Regional hospitals	0.05 (0.05, 0.05)	0.05 (0.05, 0.05)	0.05 (0.05, 0.05)	0.05 (0.05, 0.05)	0.05 (0.05, 0.05)
Hospital type (ref: other clinics)					
General hospitals, community hospitals, and other MOPH agencies	0.00 (0.00, 0.00)	0.01 (0.00, 0.01)	0.00 (0.00, 0.00)	0.00 (0.00, 0.00)	0.00 (0.00, 0.00)
Non MOPH agencies such as university hospitals	0.11 (0.1, 0.11)	0.13 (0.13, 0.14)	0.12 (0.12, 0.12)	0.11 (0.11, 0.12)	0.11 (0.11, 0.11)
Private hospitals	0.03 (0.03, 0.03)	0.07 (0.06, 0.07)	0.06 (0.06, 0.06)	0.05 (0.05, 0.06)	0.05 (0.05, 0.06)
Hospital zone: (ref: hospitals outside Bangkok)					
Bangkok	0.06 (0.06, 0.06)	0.09 (0.09, 0.09)	0.07 (0.07, 0.08)	0.07 (0.07, 0.08)	0.07 (0.06, 0.07)
Primary diagnosis					
Neoplasm	0.08 (0.08, 0.08)	0.08 (0.08, 0.08)	0.07 (0.07, 0.07)	0.07 (0.07, 0.08)	0.08 (0.07, 0.08)
Diseases of circulatory system	0.07 (0.07, 0.07)	0.07 (0.07, 0.08)	0.08 (0.08, 0.08)	0.08 (0.08, 0.08)	0.08 (0.08, 0.08)
Diseases of respiratory system	0.02 (0.02, 0.02)	0.01 (0.01, 0.01)	0.01 (0.01, 0.01)	0.01 (0.01, 0.01)	0.01 (0.01, 0.01)
Diseases of the digestive system	-0.00 (-0.00, -0.00)	-0.00 (-0.00, -0.00)	-0.00 (-0.00, -0.00)	-0.01 (-0.01, -0.01)	-0.00 (-0.00, -0.00)
Injury, or poisoning and certain other consequences of external causes	0.04 (0.04, 0.04)	0.05 (0.05, 0.05)	0.04 (0.04, 0.04)	0.04 (0.04, 0.04)	0.04 (0.04, 0.04)
Death	0.1 (0.1, 0.11)	0.08 (0.08, 0.09)	0.09 (0.08, 0.09)	0.07 (0.07, 0.08)	0.07 (0.06, 0.07)
Number of primary diagnoses	0.01 (0.01, 0.01)	0.01 (0.01, 0.01)	0.01 (0.01, 0.01)	0.01 (0.01, 0.01)	0.01 (0.01, 0.01)
Number of visits in a year	0.02 (0.02, 0.02)	0.02 (0.02, 0.02)	0.02 (0.02, 0.02)	0.02 (0.02, 0.02)	0.02 (0.02, 0.02)

Notes: Prob = Probability; 95% CI = 95% confidence interval.

For peer review only

For peer review only - http://bmjopen.bmj.com/site/about/guidelines.xhtml

Table A3 Relative risk ratio of factors predicting being high-cost users and persistent high-cost users

Factors	Factors predicting being HCUs RRR (95% CI)	Factors predicting being persistent HCUs RRR (95% CI)
Age	1.04 (1.04, 1.04)	1.08 (1.08, 1.09)
Age*Age	0.99 (0.99, 0.99)	0.99 (0.99, 0.99)
Gender (ref: female)	0.69 (0.68, 0.70)	0.80 (0.77, 0.83)
Hospital type (ref: other clinics)		
Regional hospitals	2.83 (2.8, 2.86)	3.19 (3.12, 3.26)
General hospitals, community hospitals, and other MOPH agencies	1.09 (1.07, 1.10)	0.86 (0.83, 0.88)
Non MOPH agencies such as university hospitals	5.56 (5.46, 5.66)	10.2 (9.93, 10.5)
Private hospitals	2.29 (2.23, 2.35)	3.18 (3.04, 3.32)
Hospital zone: (ref: hospitals located outside Bangkok)		
Bangkok	4.40 (4.24, 4.57)	8.15 (7.63, 8.71)
Primary diagnosis		
Neoplasm	7.91 (7.54, 8.31)	43.5 (40.3, 47.0)
Diseases of circulatory system	7.24 (6.96, 7.53)	12.4 (11.5, 13.4)
Diseases of respiratory system	1.30 (1.27, 1.33)	2.21 (2.08, 2.35)
Diseases of the digestive system	0.73 (0.7, 0.75)	0.95 (0.89, 1.02)
Injury, or poisoning and certain other consequences of external causes	1.99 (1.93, 2.04)	2.78 (2.6, 2.97)
Comorbidity (Charlson comorbidity index)	1.12 (1.12, 1.13)	1.21 (1.20, 1.22)
Death	3.03 (2.93, 3.14)	3.63 (3.43, 3.85)
Number of primary diagnoses	1.71 (1.70, 1.71)	1.75 (1.74, 1.77)
Number of visits in a year	1.06 (1.06, 1.06)	1.16 (1.16, 1.17)
Neoplasm*Death	0.56 (0.54, 0.59)	0.60 (0.56, 0.64)
Diseases of circulatory system*Death	0.51 (0.49, 0.52)	0.55 (0.53, 0.59)
Diseases of respiratory system*Death	1.39 (1.34, 1.44)	1.79 (1.70, 1.88)
Diseases of the digestive system*Death	1.12 (1.08, 1.16)	1.22 (1.16, 1.29)
Injury, or poisoning and certain other consequences of external causes*Death	0.68 (0.65, 0.71)	0.77 (0.72, 0.82)
Neoplasm*Age	0.98 (0.98, 0.98)	0.96 (0.96, 0.97)
Diseases of circulatory system*Age	0.98 (0.98, 0.98)	0.98 (0.97, 0.98)
Diseases of respiratory system*Age	0.99 (0.99, 0.99)	0.99 (0.99, 0.99)
Diseases of the digestive system*Age	1.00 (1.00, 1.00)	0.99 (0.99, 0.99)
Injury, or poisoning and certain other consequences of external causes*Age	0.99 (0.99, 0.99)	0.99 (0.99, 0.99)

For peer review only

For peer review only - http://bmjopen.bmj.com/site/about/guidelines.xhtml

Factors	Factors predicting being HCUs RRR (95% CI)	Factors predicting being persistent HCUs RRR (95% CI)
Neoplasm*Gender	0.84 (0.82, 0.87)	0.69 (0.66, 0.72)
Diseases of circulatory system*Gender	1.01 (0.99, 1.03)	0.83 (0.80, 0.87)
Diseases of respiratory system*Gender	0.95 (0.93, 0.97)	0.82 (0.79, 0.86)
Diseases of the digestive system*Gender	1.06 (1.04, 1.09)	0.97 (0.93, 1.00)
Injury, or poisoning and certain other consequences of external causes*Gender	1.19 (1.16, 1.22)	1.23 (1.18, 1.28)
Hospital zone: Bangkok* Regional hospitals	0.55 (0.53, 0.57)	0.52 (0.49, 0.55)
Hospital zone: Bangkok* General hospitals, community hospitals, and other MOPH agencies	1.31 (1.27, 1.35)	1.56 (1.48, 1.65)
Hospital zone: Bangkok* Non MOPH agencies	0.28 (0.27, 0.29)	0.19 (0.18, 0.20)
Hospital zone: Bangkok* Private hospitals	0.42 (0.40, 0.44)	0.30 (0.28, 0.32)
Constant	0.00 (0.00, 0.00)	0.00 (0.00, 0.00)

Notes: 95% CI = 95% confidence interval; HCUs = high cost users; RRR = relative risk ratio.

For peer review only - http://bmjopen.bmj.com/site/about/guidelines.xhtml

STROBE 2007 (v4) Statement—Checklist of items that should be included in reports of *cross-sectional studies*

Section/Topic	Item #	Recommendation	Reported on page #
**Title and abstract**	1	(*a*) Indicate the study's design with a commonly used term in the title or the abstract	2
		(*b*) Provide in the abstract an informative and balanced summary of what was done and what was found	2
**Introduction**			
Background/rationale	2	Explain the scientific background and rationale for the investigation being reported	4-5
Objectives	3	State specific objectives, including any prespecified hypotheses	5
**Methods**			
Study design	4	Present key elements of study design early in the paper	5
Setting	5	Describe the setting, locations, and relevant dates, including periods of recruitment, exposure, follow-up, and data collection	5
Participants	6	(*a*) Give the eligibility criteria, and the sources and methods of selection of participants	5-7
Variables	7	Clearly define all outcomes, exposures, predictors, potential confounders, and effect modifiers. Give diagnostic criteria, if applicable	5-7
Data sources/ measurement	8*	For each variable of interest, give sources of data and details of methods of assessment (measurement). Describe comparability of assessment methods if there is more than one group	5-6
Bias	9	Describe any efforts to address potential sources of bias	5-7
Study size	10	Explain how the study size was arrived at	5-6
Quantitative variables	11	Explain how quantitative variables were handled in the analyses. If applicable, describe which groupings were chosen and why	5-6
Statistical methods	12	(*a*) Describe all statistical methods, including those used to control for confounding	6-7
		(*b*) Describe any methods used to examine subgroups and interactions	6-7
		(*c*) Explain how missing data were addressed	5
		(*d*) If applicable, describe analytical methods taking account of sampling strategy	N/A
		(*e*) Describe any sensitivity analyses	N/A
**Results**			

For peer review only

Participants	13*	(a) Report numbers of individuals at each stage of study—eg numbers potentially eligible, examined for eligibility, confirmed eligible, included in the study, completing follow-up, and analysed	7
		(b) Give reasons for non-participation at each stage	N/A
		(c) Consider use of a flow diagram	N/A
Descriptive data	14*	(a) Give characteristics of study participants (eg demographic, clinical, social) and information on exposures and potential confounders	7-10
		(b) Indicate number of participants with missing data for each variable of interest	7
Outcome data	15*	Report numbers of outcome events or summary measures	7-11
Main results	16	(*a*) Give unadjusted estimates and, if applicable, confounder-adjusted estimates and their precision (eg, 95% confidence interval). Make clear which confounders were adjusted for and why they were included	7-16
		(*b*) Report category boundaries when continuous variables were categorized	7-16
		(*c*) If relevant, consider translating estimates of relative risk into absolute risk for a meaningful time period	N/A
Other analyses	17	Report other analyses done—eg analyses of subgroups and interactions, and sensitivity analyses	N/A
**Discussion**			
Key results	18	Summarise key results with reference to study objectives	16-18
Limitations	19	Discuss limitations of the study, taking into account sources of potential bias or imprecision. Discuss both direction and magnitude of any potential bias	17-18
Interpretation	20	Give a cautious overall interpretation of results considering objectives, limitations, multiplicity of analyses, results from similar studies, and other relevant evidence	16-18
Generalisability	21	Discuss the generalisability (external validity) of the study results	16-18
**Other information**			
Funding	22	Give the source of funding and the role of the funders for the present study and, if applicable, for the original study on which the present article is based	19

*Give information separately for cases and controls in case-control studies and, if applicable, for exposed and unexposed groups in cohort and cross-sectional studies.

Note: An Explanation and Elaboration article discusses each checklist item and gives methodological background and published examples of transparent reporting. The STROBE checklist is best used in conjunction with this article (freely available on the Web sites of PLoS Medicine at http://www.plosmedicine.org/, Annals of Internal Medicine at http://www.annals.org/, and Epidemiology at http://www.epidem.com/). Information on the STROBE Initiative is available at www.strobe-statement.org.

For peer review only

BMJ Open

Retrospective secondary data analysis to identify high-cost healthcare users in Thailand, a middle-income country with universal healthcare coverage

Journal:	*BMJ Open*
Manuscript ID	bmjopen-2020-047330.R1
Article Type:	Original research
Date Submitted by the Author:	26-May-2021
Complete List of Authors:	Rattanavipapong, Waranya; Health Intervention and Technology Assessment Program, Health<br>Yi, Wang; National University of Singapore<br>Butchon, Rukmanee; Health Intervention and Technology Assessment Program, Health<br>Kittiratchakool, Nitichen; Health Intervention and Technology Assessment Program<br>Thammatacharee , Jadej; National Health Security Office<br>Teerawattananon, Yot; Health Intervention and Technology Assessment Program, Health; National University of Singapore<br>Isaranuwatchai, Wanrudee; Health Intervention and Technology Assessment Program, Health
**Primary Subject Heading**:	Health policy
Secondary Subject Heading:	Evidence based practice
Keywords:	HEALTH ECONOMICS, Health policy < HEALTH SERVICES ADMINISTRATION & MANAGEMENT, PUBLIC HEALTH, STATISTICS & RESEARCH METHODS

SCHOLARONE™
Manuscripts

I, the Submitting Author has the right to grant and does grant on behalf of all authors of the Work (as defined in the below author licence), an exclusive licence and/or a non-exclusive licence for contributions from authors who are: i) UK Crown employees; ii) where BMJ has agreed a CC-BY licence shall apply, and/or iii) in accordance with the terms applicable for US Federal Government officers or employees acting as part of their official duties; on a worldwide, perpetual, irrevocable, royalty-free basis to BMJ Publishing Group Ltd ("BMJ") its licensees and where the relevant Journal is co-owned by BMJ to the co-owners of the Journal, to publish the Work in this journal and any other BMJ products and to exploit all rights, as set out in our licence.

The Submitting Author accepts and understands that any supply made under these terms is made by BMJ to the Submitting Author unless you are acting as an employee on behalf of your employer or a postgraduate student of an affiliated institution which is paying any applicable article publishing charge ("APC") for Open Access articles. Where the Submitting Author wishes to make the Work available on an Open Access basis (and intends to pay the relevant APC), the terms of reuse of such Open Access shall be governed by a Creative Commons licence – details of these licences and which Creative Commons licence will apply to this Work are set out in our licence referred to above.

Other than as permitted in any relevant BMJ Author's Self Archiving Policies, I confirm this Work has not been accepted for publication elsewhere, is not being considered for publication elsewhere and does not duplicate material already published. I confirm all authors consent to publication of this Work and authorise the granting of this licence.

For peer review only

Retrospective secondary data analysis to identify high-cost healthcare users in Thailand, a middle-income country with universal healthcare coverage

Correspondence to Wanrudee Isaranuwatchai; 6th Floor, 6th Building, Department of Health, Ministry of Public Health, Tiwanon Rd., Muang, Nonthaburi 11000, Thailand; wanrudee.i@hitap.net; +662-590-4549, +662-590-4374, +662-590-4375

Authors and Institutions

Waranya Rattanavipapong[1], Wang Yi[2], Rukmanee Butchon[1], Nitichen Kittiratchakool[1], Jadej Thammatacharee[3], Yot Teerawattananon[1], Wanrudee Isaranuwatchai[1,4]

Author affiliations

Waranya Rattanavipapong[1], Wang Yi[2], Rukmanee Butchon[1], Nitichen Kittiratchakool[1], Jadej Thammatacharee[3], Yot Teerawattananon[1,2], Wanrudee Isaranuwatchai[1,4]

[1]Health Intervention and Technology Assessment Program, Nonthaburi, Thailand
[2]Health Intervention and Policy Evaluation Research, Saw Swee Hock School of Public Health, National University of Singapore, Singapore
[3]National Health Security Office, Thailand
[4]Institute or Health Policy, Management and Evaluation, University of Toronto, Canada

Word count: 3,970/4,000

For peer review only

ABSTRACT

Objectives

The study aims to identify high-cost healthcare users (HCUs) in Thailand including their common characteristics, patterns of healthcare utilization and expenditure compared to low-cost users, and to explore potential factors associated with HCUs so the healthcare system can be prepared to support the HCUs including those who have increased chance of becoming HCUs.

Design and setting

A retrospective secondary data analysis using hospitalization data from Thailand's universal coverage scheme (UCS) obtained from the National Health Security Office over a 5-year period from October 2014 to September 2019 (fiscal year 2014-2018).

Participants

Study participants included Thai citizens who had at least one inpatient admission to hospitals under the UCS over the study period.

Results

Over the 5-year period, the top 5% of hospitalised population (or HCUs) consumed almost 50% of the health expenditure each year. HCUs were more likely to have longer hospital stays, have a higher annual number of visits, and be admitted to multiple hospitals each year when compared to the low-cost users (the bottom 50% of hospitalised population). The study further reported that the chance of becoming HCUs is associated with several factors such as increasing age, being male, having comorbidity, and being admitted to hospitals in Bangkok.

Conclusions

This study confirmed that the HCUs phenomenon existed in Thailand, where a majority of inpatient care spending under is concentrated in the top 5% of hospitalised population. The study findings call attention to potential initiatives that can help monitor the magnitude and trend of HCUs and develop policies to prevent HCUs.

For peer review only - http://bmjopen.bmj.com/site/about/guidelines.xhtml

Strengths and limitations of this study

- This study is one of the first studies to use real-world administrative hospitalization data from Thailand's universal coverage scheme to explore the high-cost users (HCUs) phenomenon, and to identify characteristics of HCUs.
- The study represents an example of how existing routinely collected real-world data can be used to potentially improve care and health service and to help address policy-relevant questions.
- The analysis was limited by data availability, and future work could explore beyond hospitalization data to consider other types of health services (e.g. outpatient services), and could include other potential confounders (e.g. education and income).
- Significant work were conducted to clean and manage the data which highlighted the importance of data quality especially in countries who may have not used real-world data to their full potential.

Keywords: high-cost users, high-cost, health care utilization, hospitalization, administrative databases, real-world data, Thailand

For peer review only

INTRODUCTION

There is an increasing emphasis on the sustainability of healthcare systems for many reasons, including the availability of new costly health technologies, the greater demand for healthcare by an aging population, and the government's commitment to achieve universal health coverage (UHC) under limited resources.[1 2] As such, several solutions have been attempted to enhance the efficiency of healthcare systems around the world.[3-6] These potential solutions include the prioritisation of health problems and associated interventions based on factors such as burden of disease and cost-effectiveness evidence,[7 8] identifying low-value health services that can be omitted without adversely affecting patient outcomes,[9 10] and promoting the use of preventive measures to avoid patients' development of chronic conditions.[11 12]

Based on a literature review of the high-cost users (HCUs) phenomenon in several countries, for example, Japan, the United States, Australia, and Canada,[13-18] HCUs are generally defined as the top 5% of patients with the highest health expenditure in a given year, while low-cost user (LCU) are defined as the bottom 50% of patients with the lowest healthcare costs.[15-17] Although the aforementioned evidence is only from high-income countries, this phenomenon is relevant and important for low- and middle-income countries (LMICs) where health resources are limited. Additionally, there are studies on health service utilization in LMICs focusing on general patterns of utilization and certain high-cost interventions;[19-21] however they do not focus on HCUs. Therefore, a better understanding of the HCUs in Thailand (an upper middle-income country (UMIC)) regarding HCUs' characteristics and patterns of healthcare utilization may help the country (including other LMICs with similar country profiles) to identify potential HCUs and develop more tailored and appropriate interventions to improve the management and financing of these patients.

Thailand has been classified as a UMIC since 2011[22] and has a rapidly growing aging population. The proportion of the population aged 60 years or over increased from 12% in 2011 to 18% in 2020[23], and is projected to increase further to 25% in 2030.[24] Leading causes of burdens of disease (cause of death and disability) in the Thai population include injury, cardiovascular disease, cancer, and diabetes mellitus.[25] These population characteristics, to some extent, align with potential factors associated with HCUs identified in the literature from high-income countries such as being older and having comorbidities.[16 18]

Although the Thai government invests approximately 13.3% of the government budget on public health,[22] the Universal Coverage Scheme (UCS) budget has increased annually during the past decade and at a faster rate than the annual gross domestic product (GDP) growth, which is directly affecting the sustainability of the healthcare system.[22 26] The three

For peer review only - http://bmjopen.bmj.com/site/about/guidelines.xhtml

main health insurance programs employed to cover nearly the full Thai population include the Civil Servant Medical Benefit Scheme (CSMBS) for government employees and their dependents, the Social Security Scheme (SSS) for formal private sector employees, and the UCS for the remaining Thai citizens.[22] The UCS, which is managed by the National Health Security Office (NHSO), covers approximately 80% of the total population who are not eligible for the CSMBS and SSS, including low-income or unemployed citizens. Characteristics of the aforementioned three health insurance programs in Thailand are published elsewhere.[22]

The UCS provides a comprehensive benefits package for curative, rehabilitation, and health promotion and prevention services.[27] Most of the UCS budget is allocated to outpatient and inpatient services. Payment for outpatient care is generally based on capitation while inpatient care payment is based on diagnosis-related groups (DRGs).[28 29] There is also an additional budget for specific high-cost interventions or medications.[30] Nearly half of the UCS expenditure (e.g. 42% in 2011) is spent on inpatient care.[26]

Research on the HCUs phenomenon under Thailand's biggest public health insurance scheme, the UCS, is essential for identifying potential measures that are deemed 'preventable' spending. This study aims to identify demographic characteristics of HCUs; understand the common characteristics and patterns of healthcare utilization and expenditure among HCUs as compared to LCUs; and determine potential factors associated with HCUs. This study is the first of its kind in UMICs and its findings have the potential to be used by healthcare managers and policy-makers in relevant settings (including LMICs) to avert avoidable HCUs and reallocate these limited resources for other cost-effective options.

METHODS

Study population, setting, and data

This study was a retrospective secondary data analysis which examined inpatient department (or hospitalization) data from Thailand's UCS obtained from the NHSO over a 5-year period from 2014 to 2018. Using this administrative hospitalization database, the study population included Thai citizens of all ages who were hospitalised under the UCS health insurance scheme and discharged between October 1st, 2014, and September 30th, 2019, according to the Thai government's fiscal year. A unique national identification (ID) number is registered for each Thai citizen in the system. Thai citizens with a national ID who had at least one hospital admission during the study period were included in the study dataset. The

For peer review only - http://bmjopen.bmj.com/site/about/guidelines.xhtml

subjects' unique IDs were masked using a private key by the data holder before the dataset was shared with the research team.

All data were anonymised and de-identified. Records with encrypted identities were checked, and duplicated records or those with incomplete data were excluded from analysis. To ensure that the data were accurate and consistent, conflicting demographic information about date of birth, gender, and admission and discharge dates were investigated and resolved. Conflicting records were removed (with the final number of records being 29,899,719).

Variables considered in the analysis comprised of: gender; date of birth; primary diagnosis (using ICD-10 codes); admission and discharge dates; discharge status (full recovery, improved, not improved, normal delivery, undelivered, normal child discharged with mother, separation of mother and baby, stillbirth, and death); types of hospitals (i.e. from the lowest level being clinics and community hospitals to the higher level, namely private hospitals, general hospitals, regional hospitals, and other hospitals not under the Ministry of Public Health, such as university hospitals); health regions as shown in the Supplementary figure 1; Charlson comorbidity index;[31 32] and reimbursement cost (which refers to the amount estimated to be paid by NHSO and reported in the hospitalization database).

This study received ethical approval from Institute for the Development of Human Research Protections, Ministry of Public Health, Thailand. This study used an existing administrative database (with de-identified individual-level hospitalization data); therefore, patients had no direct involvement or risk and no consent was required.

Patient and public involvement

No patient involved.

Analysis

Using the NHSO's hospitalization database, HCUs and persistent HCUs were examined using descriptive and multivariable regression analysis. HCUs and LCUs were defined by the percentile of hospitalization costs accrued. HCUs were individuals who had incurred costs higher than 95% of all users (the top 5%). LCUs were patients in the lowest cost group (the bottom 50%). The dataset was treated as repeated cross-sectional when examining HCUs and the analysis was conducted separately by year based on patients' discharge dates using similar methods as seen in the literature.[16 18] For example, if patients were admitted to a hospital in one fiscal year but discharged in the following fiscal year, their costs

For peer review only

would be included since the admitted fiscal year. We define persistent HCUs as those who were identified as HCUs for more than one year; further explanation is provided below. When examining persistent HCUs, a panel dataset was constructed by tracking the individuals' statuses (of being an HCU) over multiple years.

Every hospital admission was tracked within each fiscal year (October 1st to September 30th). To address the study aims, first, we sought to determine the concentration of inpatient department expenditures to explore the phenomenon of HCUs in Thailand. Total costs associated with all visits within each fiscal year for each patient (i.e. estimated reimbursement costs) were calculated and individuals were sorted into 5 groups according to the percentile of costs: 1) above the 99th percentile; 2) between the 95th and the 98th percentile; 3) between the 90th and 94th percentiles; 4) between the 50th and 89th percentiles; and 5) below the 50th percentile. Patients may have more than one hospital admission; therefore, we summed up all admissions per patients within each year to calculate the annual hospitalization cost per patient as used in the HCU grouping.

Next, we examined the differences in demographic characteristics, costs, and comorbidities of HCUs as compared to LCUs. Descriptive characteristics of each group were analysed by age, gender, and identified main diagnosis based on ICD-10 codes. We also reported healthcare utilization and expenditure patterns between HCUs and LCUs, specifically number of visits, length of stay (LOS), types of hospitals visited, and annual cost per patient.

Multivariable logistic regression was conducted to identify potential factors associated with HCUs. The outcome variable was patient HCU status (yes/no) and the exploratory variables included age, gender, number of visits, types and health regions of hospitals visited, main diagnoses, and comorbidities (measured as Charlson comorbidity index, and the presence of key primary diagnosis namely neoplasms, circulatory disease, respiratory disease, digestive problems, and injuries). Interaction terms were also included to examine the heterogeneous effects on becoming a potential HCU; specifically, interactions between death, age, or gender and the top five primary diagnoses (neoplasms, circulatory disease, respiratory disease, digestive problems, and injuries) including between types of hospital and health regions were considered.

To identify the persistent HCUs, only the data between 2015 and 2017 were examined due to the completeness of the data. To construct a panel dataset, patients who were presented in the dataset in 2015 were used for the analysis. Any patients who died were excluded as they could not become persistent HCUs. For the remaining patients, we examined their HCU status in the years 2016 and 2017 to determine whether they were persistent HCUs. They were then grouped into three levels: 0 (not an HCU for any of the three years); 1 (being an

For peer review only - http://bmjopen.bmj.com/site/about/guidelines.xhtml

HCU for only one year); and 2 (being an HCU for at least two years). Group 2 was defined as persistent HCUs. A multinomial logistic regression was applied to examine the potential factors associated with persistent HCUs.

All analyses were conducted using Stata Statistical Software (Release 15, College Station, TX). Chi-square tests and t-tests were used to test for significance of differences at p-value < 0.05.

RESULTS

A total of 29,899,719 admissions (14,464,180 unique patients) between fiscal years 2014–2018 were analysed. On average, each year had approximately 5.9 million admissions (about 3.9 million unique patients). Additional details on the data management process can be found in Supplementary figure 2.

The hospitalization data of UCS have shown that a small proportion of patients (the top 5% of hospitalised population or HCUs) consumed almost half of the healthcare expenditure; this trend was observed over a 5-year period as presented in **Figure 1**. During this period, patients in the top 1% and 5% cost groups accounted for 16—18% and 45—48% of total spending, respectively. Furthermore, a separate analysis showed that HCUs had higher annual mortality rates compared to LCUs. Among HCUs, mortality ranged from 19%—21% over 5 years, compared with 0.1%—1.3% in LCUs.

Figure 1 Distribution of total hospitalization's expenditures across cost percentiles

To facilitate the understanding of the HCU phenomenon, the profile of HCUs was studied. Generally, the average age of HCUs was 55—56 years old, where more than half (50-54% each year) were in the age group of ≥60. In Thailand, about 18% of the population were older adults (≥60). A approximately 55% of this sample were male, while approximately 49% were male in the general Thai population.[23] **Table 1** provides the descriptive characteristics of HCUs as compared with LCUs.

For peer review only - http://bmjopen.bmj.com/site/about/guidelines.xhtml

1 **Table 1 Demographic characteristics and patterns of utilization and expenditure of HCUs and LCUs from 2014 to 2018*,****

Characteristic	2014		2015		2016		2017		2018	
	HCUs	LCUs	HCUs	LCUs	HCUs	LCUs	HCUs	LCUs	HCUs	LCUs
Number of patients	234,214	1,913,712	235,954	1,964,952	243,850	1,991,451	239,180	1,953,317	243,951	1,992,271
Number of admissions	864,787	1,991,875	863,308	2,054,265	899,142	2,090,249	926,434	2,053,590	951,527	2,096,159
Mean age in years (SD)	54.6 (22.6)	22.6 (25.0)	54.5 (22.4)	24.1 (25.5)	55.4 (22.3)	23.8 (25.4)	55.6 (22.3)	24.8 (25.9)	56.1 (22.1)	25.2 (26.1)
Male (%)	55%	44%	55%	44%	55%	45%	55%	45%	55%	45%
Average length of stay in days (SD)	11.3 (21.2)	2.5 (2.8)	11.3 (22.0)	2.6 (2.1)	10.9 (21.6)	2.6 (2.9)	10.8 (19.1)	2.6 (2.6)	10.4 (18.8)	2.6 (2.3)
Average number of visits (SD)	3.7 (3.3)	1.0 (0.2)	3.7 (3.3)	1.0 (0.2)	3.7 (3.4)	1.0 (0.2)	3.9 (3.5)	1.0 (0.2)	3.9 (3.5)	1.0 (0.2)
Number of hospitals visited (% of admissions)										
1	49.4%	99.0%	49.7%	99.0%	49.8%	99.0%	48.5%	99.0%	48.4%	99.0%
2	41.3%	1.0%	40.4%	1.0%	40.7%	1.0%	41.6%	1.0%	41.8%	1.0%
3	8.1%	-	8.6%	-	8.3%	-	8.7%	-	8.6%	-
>3	1.2%	-	1.3%	-	1.2%	-	1.2%	-	1.2%	-
Types of hospitals visited (% of admissions)										
Regional hospitals	35.5%	16.2%	33.6%	16.2%	34.9%	15.8%	34.0%	16.3%	34.3%	16.0%
General hospitals	21.2%	21.8%	20.0%	22.0%	20.9%	21.7%	21.5%	22.0%	22.4%	21.8%
Community hospitals	20.1%	53.3%	18.7%	54.2%	19.6%	54.1%	20.7%	53.3%	21.4%	53.7%
Others	23.2%	8.7%	27.7%	7.6%	24.6%	8.4%	23.8%	8.4%	21.9%	8.5%

For peer review only - http://bmjopen.bmj.com/site/about/guidelines.xhtml

Characteristic	2014		2015		2016		2017		2018	
	HCUs	LCUs	HCUs	LCUs	HCUs	LCUs	HCUs	LCUs	HCUs	LCUs
Average total cost per patient in baht (SD)	130,700 (102,500)	2,800 (1,070)	107,600 (107,600)	2,300 (820)	121,300 (88,500)	2,600 (970)	123,800 (87,100)	2,700 (1,060)	127,700 (88,800)	2,900 (1,130)

HCUs = high-cost users; LCU = low cost users; SD = standard deviation.

*The number (except number of patients and admissions) presented in table was rounded to the nearest whole number.

**All variables are significant (p-value < 0.05).

For peer review only - http://bmjopen.bmj.com/site/about/guidelines.xhtml

Over the study period, HCUs were older and included more men than compared to LCUs. The mean age ranged between 55—56 years and 23—25 years among HCUs and LCUs, respectively. HCUs had longer lengths of stay and used more healthcare services (higher frequency of hospital visits and higher number of visited hospitals). Moreover, HCUs visited high level hospitals (e.g. regional and university hospitals), while LCUs visited predominantly community hospitals. The pattern of the results were similar across the 5-year period.

Regarding the expenditure pattern, HCUs spent considerably more per year than LCUs in the index year. Over 5 years, the average annual spending for HCUs was between 107,600 (± 107,600) to 130,700 (± 102,500) Baht, compared to 2,300 (± 820) to 2,900 (± 1,130) Baht for LCUs, where 1 US dollar is approximately 32 Thai baht. Details of the expenditure pattern are presented in **Table 1**.

The most common and most expensive clinical conditions between HCUs and LCUs were examined and found to differ. **Table 2** confirms that the expenditures were spent on different diagnoses. Among HCUs, the most frequent diagnoses included neoplasms and diseases of the circulatory system and respiratory system which consumed more than half of annual expenditure. Among LCUs, the most frequent diagnoses were broader and included infectious and parasitic diseases, factors influencing health status and contact with health services (e.g. admissions for investigation), and diseases of the respiratory system; almost half of LCUs' annual expenditure was spent on these conditions.

For peer review only - http://bmjopen.bmj.com/site/about/guidelines.xhtml

1 **Table 2 The top five primary diagnoses of HCUs and LCUs over a 5-year study period***

High-cost users (HCUs)			Low cost users (LCUs)		
**Conditions**	**% of total admissions**	**% of total expenditures**	**Conditions**	**% of total admissions**	**% of total expenditures**
Neoplasm	22-23	17-20	Certain infectious and parasitic diseases	17-19	15-17
Diseases of circulatory system	17-20	25-30	Factors influencing health status and contact with health services	14-17	9-12
Diseases of respiratory system	13-14	10-12	Diseases of the respiratory system	11-14	13-17
Diseases of genitourinary system	7-8	3	Pregnancy, childbirth, puerperium	10-12	11-14
Diseases of the digestive system	7	5	Injury, poisoning and certain other consequences of external causes	9	8-9

2 HCUs = high-cost users; LCU = low cost users.

3 *The findings reported covered the 5-year study period so the lowest percentage and highest percentage during this period were presented.

For peer review only - http://bmjopen.bmj.com/site/about/guidelines.xhtml

Regression models were used to examine the potential factors associated with being an HCU (**Table 3**). The findings suggest that age, gender, types and zones of hospital admissions, disease and multimorbidity, number of visits, and mortality were associated with becoming an HCU (although the relative contribution of these factors towards the expenditure varied). Most of the interaction terms were statistically significant, suggesting that the impact of these factors were heterogenous among different groups.

For peer review only

For peer review only - http://bmjopen.bmj.com/site/about/guidelines.xhtml

Table 3 Factors associated with high-cost users in UCS*

Factors Associated with Being High-cost User	2014	2015	2016	2017	2018
	OR (95%CI)	OR (95%CI)	OR (95%CI)	OR (95%CI)	OR (95%CI)
Age	1.03 (1.03, 1.03)	1.03 (1.03, 1.04)	1.03 (1.03, 1.04)	1.03 (1.03, 1.03)	1.03 (1.03, 1.03)
Age*Age	0.99 (0.99, 0.99)	0.99 (0.99, 0.99)	0.99 (0.99, 0.99)	0.99 (0.99, 0.99)	0.99 (0.99, 0.99)
Gender (ref: female)	0.73 (0.71, 0.74)	0.77 (0.75, 0.78)	0.79 (0.78, 0.80)	0.79 (0.78, 0.80)	0.82 (0.81, 0.83)
Hospital type (ref: other clinics)					
Regional hospitals	3.42 (3.37, 3.47)	3.45 (3.40, 3.50)	3.47 (3.42, 3.52)	3.24 (3.19, 3.29)	3.16 (3.11, 3.20)
General hospitals, community hospitals, and other MOPH agencies	1.01 (1.00, 1.03)[+]	1.03 (1.02, 1.05)	1.00 (0.98, 1.02)[+]	1.02 (1.00, 1.03)	1.02 (1.01, 1.04)
Non MOPH agencies such as university hospitals	6.60 (6.45, 6.75)	9.06 (8.86, 9.26)	7.51 (7.35, 7.69)	7.21 (7.04, 7.37)	6.42 (6.28, 6.57)
Private hospitals	2.10 (2.03, 2.18)	4.06 (3.94, 4.18)	3.46 (3.35, 3.56)	3.28 (3.17, 3.40)	3.15 (3.04, 3.26)
Hospital zone: (ref: hospitals outside Bangkok)					
Bangkok	3.12 (2.97, 3.29)	4.73 (4.51, 4.97)	3.8 (3.61, 3.99)	3.92 (3.73, 4.12)	3.25 (3.09, 3.42)
Primary diagnosis					
Neoplasm	9.30 (8.78, 9.85)	8.96 (8.46, 9.49)	9.09 (8.59, 9.62)	8.77 (8.28, 9.28)	10.0 (9.49, 10.6)
Diseases of circulatory system	9.48 (9.01, 9.97)	9.6 (9.13, 10.0)	10.5 (10.0, 11.1)	11.0 (10.4, 11.5)	10.9 (10.4, 11.5)
Diseases of respiratory system	1.01 (0.98, 1.05)	1.00 (0.96, 1.03)	1.04 (1.00, 1.07)	0.99 (0.96, 1.02)[+]	1.11 (1.07, 1.15)
Diseases of the digestive system	0.62 (0.59, 0.65)	0.61 (0.58, 0.65)	0.62 (0.59, 0.65)	0.62 (0.59, 0.65)	0.61 (0.58, 0.64)
Injury, or poisoning and certain other consequences of external causes	2.41 (2.33, 2.50)	2.45 (2.37, 2.54)	2.37 (2.29, 2.46)	2.1 (2.02, 2.18)	2.26 (2.17, 2.34)
Comorbidity (Charlson comorbidity index)	1.07 (1.07, 1.08)	1.05 (1.04, 1.05)	1.05 (1.04, 1.05)	1.03 (1.03, 1.04)	1.02 (1.01, 1.02)
Death	6.53 (6.34, 6.73)	5.42 (5.26, 5.58)	5.43 (5.28, 5.59)	4.73 (4.59, 4.88)	4.06 (3.94, 4.19)
Number of primary diagnoses	1.20 (1.19, 1.21)	1.25 (1.24, 1.26)	1.24 (1.23, 1.25)	1.27 (1.26, 1.28)	1.28 (1.27, 1.29)

For peer review only

Factors Associated with Being High-cost User	2014	2015	2016	2017	2018
	OR (95%CI)	OR (95%CI)	OR (95%CI)	OR (95%CI)	OR (95%CI)
Number of visits in a year	1.65 (1.64, 1.66)	1.57 (1.56, 1.58)	1.55 (1.54, 1.56)	1.56 (1.56, 1.57)	1.54 (1.54, 1.55)
Neoplasm*Death	0.31 (0.30, 0.33)	0.38 (0.36, 0.39)	0.39 (0.37, 0.41)	0.39 (0.38, 0.41)	0.43 (0.41, 0.45)
Diseases of circulatory system*Death	0.36 (0.35, 0.37)	0.40 (0.39, 0.42)	0.35 (0.33, 0.36)	0.39 (0.38, 0.41)	0.44 (0.42, 0.46)
Diseases of respiratory system*Death	2.00 (1.92, 2.08)	1.59 (1.53, 1.66)	1.75 (1.68, 1.81)	1.50 (1.45, 1.56)	1.44 (1.39, 1.50)
Diseases of the digestive system*Death	1.09 (1.04, 1.14)	1.2 (1.15, 1.26)	1.12 (1.07, 1.18)	1.22 (1.16, 1.28)	1.38 (1.31, 1.44)
Injury, or poisoning and certain other consequences of external causes*Death	0.75 (0.71, 0.79)	0.64 (0.6, 0.67)	0.63 (0.59, 0.66)	0.71 (0.67, 0.75)	0.76 (0.72, 0.80)
Neoplasm*Age	0.99 (0.99, 0.99)	0.99 (0.99, 0.99)	0.99 (0.99, 0.99)	0.99 (0.99, 0.99)	0.99 (0.98, 0.99)
Diseases of circulatory system*Age	0.98 (0.98, 0.98)	0.98 (0.98, 0.98)	0.98 (0.98, 0.98)	0.98 (0.98, 0.98)	0.98 (0.98, 0.98)
Diseases of respiratory system*Age	1.01 (1.01, 1.01)	1.00 (1.00, 1.00)	1.00 (1.00, 1.01)	1.00 (1.00, 1.00)	1.00 (1.00, 1.00)
Diseases of the digestive system*Age	1.01 (1.01, 1.01)	1.00 (1.00, 1.01)	1.01 (1.00, 1.01)	1.00 (1.00, 1.01)	1.00 (1.00, 1.01)
Injury, or poisoning and certain other consequences of external causes*Age	0.99 (0.99, 1.00)[+]	1.00 (1.00, 1.00)	1.00 (1.00, 1.00)	1.00 (1.00, 1.00)	1.00 (0.99, 1.00)[+]
Neoplasm*Gender	0.84 (0.82, 0.87)	0.75 (0.72, 0.77)	0.71 (0.69, 0.74)	0.71 (0.69, 0.73)	0.66 (0.64, 0.68)
Diseases of circulatory system*Gender	1.00 (0.98, 1.03)[+]	0.9 (0.88, 0.92)	0.91 (0.89, 0.93)	0.90 (0.88, 0.92)	0.88 (0.86, 0.91)
Diseases of respiratory system*Gender	0.98 (0.95, 1.01)[+]	0.98 (0.95, 1.01)[+]	0.94 (0.91, 0.96)	0.93 (0.91, 0.96)	0.91 (0.89, 0.93)
Diseases of the digestive system*Gender	1.07 (1.04, 1.11)	1.06 (1.03, 1.1)	1.02 (0.99, 1.05)[+]	1.03 (1.00, 1.07)	1.01 (0.98, 1.05)[+]
Injury, or poisoning and certain other consequences of external causes*Gender	1.17 (1.13, 1.21)	1.2 (1.16, 1.24)	1.21 (1.18, 1.25)	1.23 (1.19, 1.26)	1.22 (1.19, 1.26)
Hospital zone: Bangkok* Regional hospitals	0.62 (0.59, 0.66)	0.57 (0.54, 0.61)	0.59 (0.56, 0.63)	0.59 (0.56, 0.63)	0.66 (0.62, 0.69)
Hospital zone: Bangkok* General hospitals, community hospitals, and other MOPH agencies	1.79 (1.71, 1.88)	1.88 (1.79, 1.96)	1.83 (1.75, 1.92)	1.69 (1.61, 1.76)	1.7 (1.62, 1.78)
Hospital zone: Bangkok* Non MOPH agencies	0.30 (0.29, 0.32)	0.23 (0.22, 0.24)	0.26 (0.24, 0.27)	0.25 (0.24, 0.27)	0.3 (0.29, 0.32)

For peer review only

For peer review only - http://bmjopen.bmj.com/site/about/guidelines.xhtml

Factors Associated with Being High-cost User	2014	2015	2016	2017	2018
	**OR (95%CI)**	**OR (95%CI)**	**OR (95%CI)**	**OR (95%CI)**	**OR (95%CI)**
Hospital zone: Bangkok* Private hospitals	0.54 (0.51, 0.58)	0.28 (0.27, 0.30)	0.34 (0.32, 0.36)	0.33 (0.31, 0.35)	0.32 (0.30, 0.34)
Constant	0.00 (0.00, 0.00)	0.00 (0.00, 0.00)	0.00 (0.00, 0.00)	0.00 (0.00, 0.00)	0.00 (0.00, 0.00)

95% CI = 95% confidence interval; OR = odds ratio.

+P-value not statistically significant (>0.05).

For peer review only

On average, HCUs were more likely to be: 1) older; 2) male; 3) admitted to higher level hospitals (e.g. regional and university hospitals); 4) admitted to hospitals in Bangkok; 5) patient with a primary malignant tumour, diseases of the circulatory system, injury, poisoning and certain other consequences of external causes; 6) patients with multiple diagnosis; 7) patients with other comorbidities; 8) patients with more than one hospitalization within a year; and 9) patients who died before their discharge. The marginal effects are presented in the Supplementary table 1, showing that the results, in term of the probability of being an HCU for average individuals, were in line with the logistic regression. For example, in 2014, an average increase in age by one year had a predicted 0.03% higher chance of becoming an HCU. The average patient with neoplasm (a primary malignant tumour) had a predicted 8% higher chance of being an HCU.

The interactions of each of the potential factors associated with HCU status showed that individuals who died before their discharge having had diseases of the respiratory system (OR = 1.39–2.08) or of the digestive system (OR = 1.04–1.44) were more likely to be HCUs. Moreover, those who were admitted to a general hospital, community hospital, or other MOPH agencies in Bangkok were also more likely to be HCUs.

This study also examined potential identifying factors of individuals who were likely to remain HCUs over time. In the data, all the persistent HCUs were HCUs for at least two years; no patient was found to be an HCU for three consecutive years. From a descriptive analysis, the average annual cost was higher for persistent HCUs (52,867 ± 74,024) as compared to those who were once off HCUs (24,577 ± 50,495). In a multinomial logistic regression, the conditions that were associated with a higher likelihood of becoming a persistent HCU were similar to the factors associated with HCUs as shown in the Supplementary table 2. However, the magnitude of the impact differed in some cases. For example, the relative risk ratio (RRR) for neoplasms was 7.9 for HCUs, but was 43.5 for persistent HCUs. This finding suggests that patients with neoplasms were likely to be HCUs, and HCUs with neoplasms were also likely to be persistent HCUs.

DISCUSSION

There is a need for understanding the characteristics of HCUs with the highest need in terms of consuming healthcare resources and their underlying factors that may be influencing costs. Recognizing that among all HCUs, some may truly need those health services whereas some may not. This study first confirmed that the HCUs phenomenon existed in Thailand, an upper middle-income country, where a majority of inpatient care spending is concentrated in the top 5% of the hospitalised population; therefore, our results are comparable to the existing literature on this phenomenon globally.

For peer review only - http://bmjopen.bmj.com/site/about/guidelines.xhtml

For peer review only

The factors associated with HCUs appear to align with those reported in the international literature. Older patients and males were more likely to be HCUs than younger and female patients, as compared to a study by Rosella et al. in Canada.[16] HCUs were also likely to have multiple diagnoses, comorbidity conditions, and serious types of diagnoses (e.g. cancer and cardiovascular diseases), and therefore, they were likely to also be admitted to hospitals in Bangkok (the capital of Thailand) with more advanced treatments available. Having chronic conditions (e.g. cancer and respiratory diseases) also increased the probability of being a persistent HCU (i.e. more than one year) as found in the published literature of high-income OECD countries.[17] Furthermore, the actual costs of healthcare resources consumed by these hospitals should be explored to ensure that the expense paid by NHSO using the DRG approach is adequate for developing and maintaining referral systems, and preventing hospitals from rejecting referred patients.[33] Additionally, the DRG creep, which is a method of modifying clinical coding practices to maximize reimbursement, should be investigated as well.[28 34] Knowing the common diagnoses of HCUs could also underline the associated costs or burden of these health conditions. As frequent users of the healthcare system, HCUs would be expected to visit a hospital more than once a year, as the findings have shown. Information on these potential factors associated with HCUs could assist healthcare professionals and hospitals in creating more tailored treatments and management plans for patients who are likely to become HCUs.

This study includes multiple strengths and limitations for future research. To our knowledge, this study is the first to confirm the HCU phenomenon in UMICs, and it is hoped that the results could be applicable to other settings (with similar country profiles), especially low-income countries which may be facing limited resources and need evidence to improve the efficiency of their healthcare system. Knowing characteristics of those in need would equip the healthcare system to respond accordingly; for example, healthcare providers could design more tailored and appropriate care to the patients. This research represents an example of how existing administrative databases could be used to answer policy-relevant questions.

This study highlights the importance of data quality in administrative databases. There were issues regarding the completeness of data between 2014 and 2018. Year 2014 was the first year that data were computerised; therefore, the data were not complete as the data entering had just started. Claims data from hospitals are usually collected by hospitals through medical charts (which can take time) and entered into NHSO system at a later date (after being checked for accuracy through the NHSO auditing steps); therefore, the data in 2018 are not completely integrated. Regardless, it is useful to include data from 2014 and 2018 to assist in investigating the magnitude and trend of HCUs. Moreover, the analyses

For peer review only - http://bmjopen.bmj.com/site/about/guidelines.xhtml

encountered several inaccuracies, such as conflicting sexes or dates of birth of individuals with multiple hospitalizations. Therefore, it is integral that when linking across databases (hospitalization and death databases), a data quality check is put in place to ensure the accurate merging of data; this step should be standard practice for similar analyses.

Additionally, as a secondary data analysis, this study was limited by data availability. Consequently, there could be additional potential confounders which were not included due to lack of data (e.g. education and income); this is a limitation that future research should consider. For example, if we were able to link the database to an outpatient visit database, the analysis may be able to examine potential factors associated with HCUs at the ambulatory-care stage as well, considering rehabilitation and prevention and promotion programs. Consequently, patients who died before their discharge were also determined to have a higher chance of being HCUs. This factor (died before discharged) may not be as informative in assisting the development of treatment plans as it is related to the outcome. In the model without this death factor, other factors (mentioned above) remain to be significant in helping to identify potential HCUs. Only in-hospital mortality was included as hospital admission data for this study; thus, future research should consider national mortality data as well. In addition, selected high-cost specialty medicines and interventions (such as high-cost medicines E2 access program[30] and stents[35]) were not included in the analysis.These interventions are procured centrally by NHSO and distributed directly to hospitals. Therefore, they are not included in the claims data and could be explored in future research for more detailed results. Furthermore, the categorization of diagnoses was achieved based on the DRG system which thus led to rather broad categories. This approach was meant to represent initial exploration on common diagnoses among HCUs; therefore, future research could explore diagnoses in a more detailed manner. The current analysis could not distinguish between those who are in true need (for high-cost treatments) versus those whom the healthcare system can support to avoid unnecessary hospitalization. Some diseases may incur substantial costs over a lifetime (e.g. cystic fibrosis), whereas others may incur very high costs in a short amount of time. Further analysis to incorporate these details could add to the understanding of HCUs and, subsequently, further assist the planning of healthcare resources, including highlighting the importance of having a data system with long-term data which allows for exploration of long-term health and economic impacts.

These findings could be applied to several scenarios to support policy-making processes. For example, knowing who the HCUs are could help health professionals monitor the magnitude and trend of HCUs and consider the HCU phenomenon when developing relevant policies such as UCS reimbursement policies to reduce avoidable HCU or improve

For peer review only - http://bmjopen.bmj.com/site/about/guidelines.xhtml

the outcomes of high-cost interventions. Moreover, a mechanism could be built to support tertiary hospitals in identifying HCUs upon admission and developing measures to support them on a routine basis as opposed to ad hoc monitoring. This mechanism can also make it easier to consider whether to provide aggressive treatment to patients with low probabilities of survival (e.g. higher Charlson comorbidity score or with multiple organ failure), especially in palliative care management. Furthermore, public communications should be made to individuals in society to raise awareness and encourage the prevention of risk factors through publicly funded prevention and promotion (P&P) programs in support of avoiding future comorbidities or other chronic diseases (e.g. cancer).[36]

In addition, in the healthcare system without UHC, the Thai government could explore having people with risk factors of HCUs be one of the priority groups so they can monitor and ensure that the patients are receiving appropriate care before they become HCUs. Low-value care is also of concern in healthcare systems,[9 10 37] and a study of its usage among HCUs could explore the significance of these low-value care services in this population. Further work on referral systems across different levels of health facilities and other types of services (in addition to hospitalization), including how to provide appropriate care at the right time and right place, could provide evidence to assist policy-makers in their planning and delivery of health services. Exploration of HCUs and their transitions across years could further develop the understanding of those with expensive necessities.

CONCLUSION

This study confirmed the HCU phenomenon in Thailand under UCS and reported the characteristics of HCUs. Factors contributing to being HCUs were being male, older, having cormorbidities, longer hospital stays, higher annual number of visits, being admitted to multiple hospitals, and having primary diagnoses such as neoplasm, diseases of the circulatory system, injury, or poisoning. Currently, HCUs (the top 5% of hospitalised patients) consumed almost 50% of the healthcare expenditure each year in Thailand. With a better understanding of HCUs, healthcare managers and policy-makers may be more equipped to assist patients by providing more tailored and targeted interventions.

Acknowledgements

The authors would like to thank the National Health Security Office (including Ms. Kanjana Sirigomon, Ms. Jutatip Thungthong, and Mr. Poonchana Wareechi) for providing the data and support for this study; Dr. Lou Jing, a research fellow at Health Intervention and Policy Evaluation Research, Saw Swee Hock School of Public Health, National University of

For peer review only - http://bmjopen.bmj.com/site/about/guidelines.xhtml

Singapore, for providing support for the data cleaning steps; and Mr. Sarin KC from HITAP for proofreading support.

Health Intervention and Technology Assessment Programme (HITAP) and Saw Swee Hock School of Public Health (SSHSPH) are part of the International Decision Support Initiative (https://www.idsihealth.org/), which supports countries to get the best value for money from health spending. International Decision Support Initiative receives funding support from the Bill & Melinda Gates Foundation, the UK Department for International Development, and the Rockefeller Foundation.

Contributors

The study was conceptualised by YT and WI. WR, WY, RB, NK, and JT contributed to its design. Funding was acquired by YT. JT was instrumental in providing data access. The analyses and interpretation were carried out by WR, WY, RB, under supervision of YT and WI. NK provided assistance with presentation of the findings. The initial draft was written by WR and WI, and refined with input from WY, RB, NK, JT and YT. All authors read and approved the final manuscript.

Funding

This study was supported by the Thailand Research Fund under the senior research scholar on Health Technology Assessment grant numbers [RTA5980011] and the Bill & Melinda Gates Foundation, the UK Department for International Development, and the Rockefeller Foundation grant number [OPP1202541] through the International Decision Support Initiative (https://www.idsihealth.org/).

Disclaimer

The findings, interpretations and conclusions expressed in this article are of the authors and do not necessarily reflect the views of the aforementioned funding agencies.

Competing interests

The authors declared no competing interests relevant to the submitted manuscript.

Ethics approval

The research was approved by Institute for the Development of Human Research Protections (IHRP No.795/2561, Date of approval on 22 November 2018) and complied with the Non-disclosure agreement between National Health Security Office and Health Intervention and Technology Assessment Program to protect individual identification.

For peer review only

Data sharing statement

The data are not publicly available due to privacy or ethical restrictions according to the data sharing agreement with the National Health Security Office.

For peer review only

For peer review only - http://bmjopen.bmj.com/site/about/guidelines.xhtml

REFERENCES

1. Borgonovi E, Compagni A. Sustaining universal health coverage: the interaction of social, political, and economic sustainability. *Value Health* 2013;16(1 Suppl):S34-8. doi: 10.1016/j.jval.2012.10.006 [published Online First: 2013/01/18]

2. Kabaniha GA, Ataguba JE-O, Kutzin J. Global Healthcare Financing. In: Haring R, Kickbusch I, Ganten D, et al., eds. Handbook of Global Health. Cham: Springer International Publishing 2020:1-42.

3. Abdirahman AS, Sagwa EL. Influence of resource mobilization on sustainability of universal health coverage in vulnerable livelihoods in wajir county, Kenya 2020 [updated 8 Jul 2020. Available from: https://cedred.org/images/Issues/Jul_Aug_Sept_2020/PDF_Abdullahi__Sagwa_Influence_of_Resource_Mobilization_on_Sustainability_of_Universal_Health_Coverage_in_in_Wajir_County_Kenya.pdf [Accessed 5 May 2021].

4. Chunharas S. Making Thailand's UHC 'SAFE' (sustainable, affordable, fair and efficient). *Global Social Policy* 2020;20(2):231-36. doi: 10.1177/1468018120922225

5. Ezoe S. Universal health coverage in japan: leveraging technologies to ensure equitable, affordable, and quality health care for all. In: Murthy P, Ansehl A, eds. Technology and Global Public Health: Springer, Cham 2020:388.

6. Tegama N. Sustainable resource mobilisation to strengthen global healthcare interventions: South African Institute of International Affairs; 2020 [updated 17 Dec 2020. Available from: https://www.africaportal.org/publications/sustainable-resource-mobilisation-strengthen-global-healthcare-interventions/ [Accessed 5 May 2021].

7. Watkins DA, Jamison DT, Mills T, et al. Universal Health Coverage and Essential Packages of Care. In: rd, Jamison DT, Gelband H, et al., eds. Disease Control Priorities: Improving Health and Reducing Poverty. Washington (DC): The International Bank for Reconstruction and Development, The World Bank 2017.

8. Teerawattananon Y, Kingkaew P, Koopitakkajorn T, et al. Development of a Health Screening Package Under the Universal Health Coverage: The Role of Health Technology Assessment. *Health Econ* 2016;25 Suppl 1(Suppl Suppl 1):162-78. doi: 10.1002/hec.3301 [published Online First: 2016/01/17]

9. Badgery-Parker T, Pearson SA, Chalmers K, et al. Low-value care in Australian public hospitals: prevalence and trends over time. *BMJ Qual Saf* 2019;28(3):205-14. doi: 10.1136/bmjqs-2018-008338 [published Online First: 2018/08/08]

For peer review only - http://bmjopen.bmj.com/site/about/guidelines.xhtml

10. Schwartz AL, Landon BE, Elshaug AG, et al. Measuring low-value care in Medicare. *JAMA Intern Med* 2014;174(7):1067-76. doi: 10.1001/jamainternmed.2014.1541 [published Online First: 2014/05/14]

11. Breslow L. From disease prevention to health promotion. *JAMA* 1999;281(11):1030-3. doi: 10.1001/jama.281.11.1030 [published Online First: 1999/03/23]

12. Labarthe DR, Dunbar SB. Global cardiovascular health promotion and disease prevention: 2011 and beyond. *Circulation* 2012;125(21):2667-76. doi: 10.1161/CIRCULATIONAHA.111.087726 [published Online First: 2012/05/31]

13. Calver J, Brameld KJ, Preen DB, et al. High-cost users of hospital beds in Western Australia: a population-based record linkage study. *Med J Aust* 2006;184(8):393-7. doi: 10.5694/j.1326-5377.2006.tb00289.x [published Online First: 2006/04/19]

14. Kozyrskyj A, Lix L, Dahl M, et al. High-cost users of pharmaceuticals : who are they? Manitoba: Manitoba Centre for Health Policy, University of Manitoba, 2005.

15. Rais S, Nazerian A, Ardal S, et al. High-cost users of Ontario's healthcare services. *Healthc Policy* 2013;9(1):44-51. [published Online First: 2013/08/24]

16. Rosella LC, Fitzpatrick T, Wodchis WP, et al. High-cost health care users in Ontario, Canada: demographic, socio-economic, and health status characteristics. *BMC Health Serv Res* 2014;14:532. doi: 10.1186/s12913-014-0532-2 [published Online First: 2014/11/02]

17. Tanke MAC, Feyman Y, Bernal-Delgado E, et al. A challenge to all. A primer on inter-country differences of high-need, high-cost patients. *PLoS One* 2019;14(6):e0217353. doi: 10.1371/journal.pone.0217353 [published Online First: 2019/06/20]

18. Wodchis WP, Austin PC, Henry DA. A 3-year study of high-cost users of health care. *CMAJ* 2016;188(3):182-88. doi: 10.1503/cmaj.150064 [published Online First: 2016/01/13]

19. Brinda EM, Kowal P, Attermann J, et al. Health service use, out-of-pocket payments and catastrophic health expenditure among older people in India: the WHO Study on global AGEing and adult health (SAGE). *J Epidemiol Community Health* 2015;69(5):489-94. doi: 10.1136/jech-2014-204960 [published Online First: 2015/01/13]

20. Peltzer K, Williams JS, Kowal P, et al. Universal health coverage in emerging economies: findings on health care utilization by older adults in China, Ghana, India, Mexico, the Russian Federation, and South Africa. *Glob Health Action* 2014;7:25314. doi: 10.3402/gha.v7.25314 [published Online First: 2014/11/05]

21. Ruiz R, Strasser-Weippl K, Touya D, et al. Improving access to high-cost cancer drugs in Latin America: Much to be done. *Cancer* 2017;123(8):1313-23. doi: 10.1002/cncr.30549 [published Online First: 2017/02/10]

For peer review only

22. Tangcharoensathien V, Witthayapipopsakul W, Panichkriangkrai W, et al. Health systems development in Thailand: a solid platform for successful implementation of universal health coverage. *Lancet* 2018;391(10126):1205-23. doi: 10.1016/s0140-6736(18)30198-3 [published Online First: 2018/02/06]

23. National Statistical Office. Size and structure of the population by age and sex 2020 [Available from: http://statbbi.nso.go.th/staticreport/page/sector/en/01.aspx [Accessed 2 May 2021].

24. Office of the National Economic and Social Development Council. Report of the Population Projections for Thailand 2010-2040 (Revision). Bangkok: Office of the National Economic and Social Development Council, 2019.

25. The Thai Working Group on Burden of Disease and Injury. Burden of disease and injury of the Thai population 2014. Nonthaburi: International Health Policy Program, Ministry of Public Health, 2017.

26. Hanvoravongchai P. Health financing reform in Thailand: toward universal coverage under fiscal constraints: World Bank; 2013 [Available from: http://hdl.handle.net/10986/13298 [Accessed 2 May 2021].

27. Mohara A, Youngkong S, Velasco RP, et al. Using health technology assessment for informing coverage decisions in Thailand. *J Comp Eff Res* 2012;1(2):137-46. doi: 10.2217/cer.12.10 [published Online First: 2012/03/01]

28. Pongpirul K, Walker DG, Winch PJ, et al. A qualitative study of DRG coding practice in hospitals under the Thai Universal Coverage Scheme. *BMC Health Serv Res* 2011;11(1):71. doi: 10.1186/1472-6963-11-71

29. Pannarunothai S. Diagnosis Related Groups (DRGs) Development in Thailand. In: Pramualratana P, Wibulpolpraser S, eds. Health insurance systems in Thailand. Nonthaburi: Health Systems Research Institute (HSRI) 2002:143-54.

30. Sruamsiri R, Wagner AK, Ross-Degnan D, et al. Expanding access to high-cost medicines through the E2 access program in Thailand: effects on utilisation, health outcomes and cost using an interrupted time-series analysis. *BMJ Open* 2016;6(3):e008671. doi: 10.1136/bmjopen-2015-008671 [published Online First: 2016/03/19]

31. Charlson M, Szatrowski TP, Peterson J, et al. Validation of a combined comorbidity index. *J Clin Epidemiol* 1994;47(11):1245-51. doi: 10.1016/0895-4356(94)90129-5 [published Online First: 1994/11/01]

32. D'Hoore W, Bouckaert A, Tilquin C. Practical considerations on the use of the Charlson comorbidity index with administrative data bases. *J Clin Epidemiol* 1996;49(12):1429-33. doi: 10.1016/s0895-4356(96)00271-5 [published Online First: 1996/12/01]

33. Sriratanaban J. Impact of universal coverage policy implementation on public hospitals, and their responses that affect medical services Nonthaburi: Health

For peer review only

Systems Research Institute; 2012 [updated 20 Apr 2012. Available from: https://www.hsri.or.th/sites/default/files/browse/tor5-1-2.pdf [Accessed 2 May 2021].

34. Pongpirul K, Walker DG, Rahman H, et al. DRG coding practice: a nationwide hospital survey in Thailand. *BMC Health Serv Res* 2011;11:290. doi: 10.1186/1472-6963-11-290 [published Online First: 2011/11/02]

35. Ingun P, Boongerd P. Thailand health information system improvement through universal health coverage implementation. *J Thai Med Inform Assoc* 2015;2:137-47.

36. Faramnuayphol P. Impact of UCS on public health Nonthaburi: Health Systems Research Institute; 2012 [updated 20 Apr 2012. Available from: https://www.hsri.or.th/sites/default/files/browse/tor5-1-3.pdf [Accessed 2 May 2021].

37. Bhatia RS, Levinson W, Shortt S, et al. Measuring the effect of Choosing Wisely: an integrated framework to assess campaign impact on low-value care. *BMJ Qual Saf* 2015;24(8):523-31. doi: 10.1136/bmjqs-2015-004070 [published Online First: 2015/06/21]

For peer review only

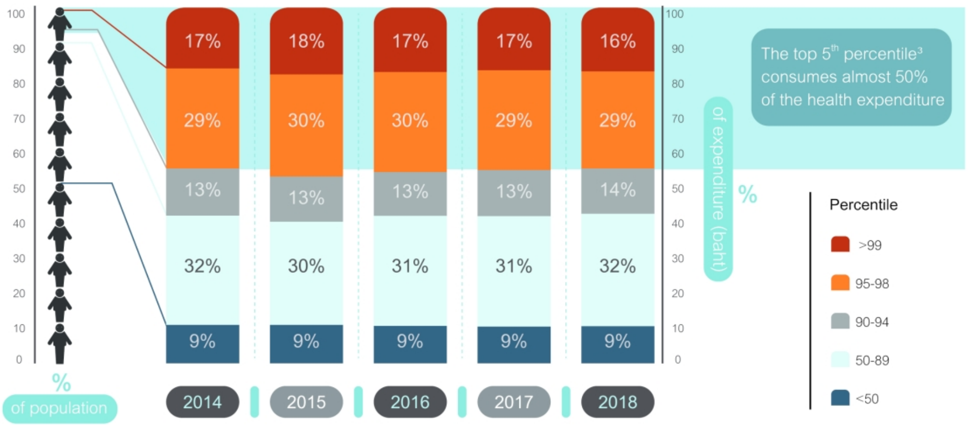

Figure 1 Distribution of total hospitalization's expenditures across cost percentiles

Supplementary figure 1 Different health regions in Thailand

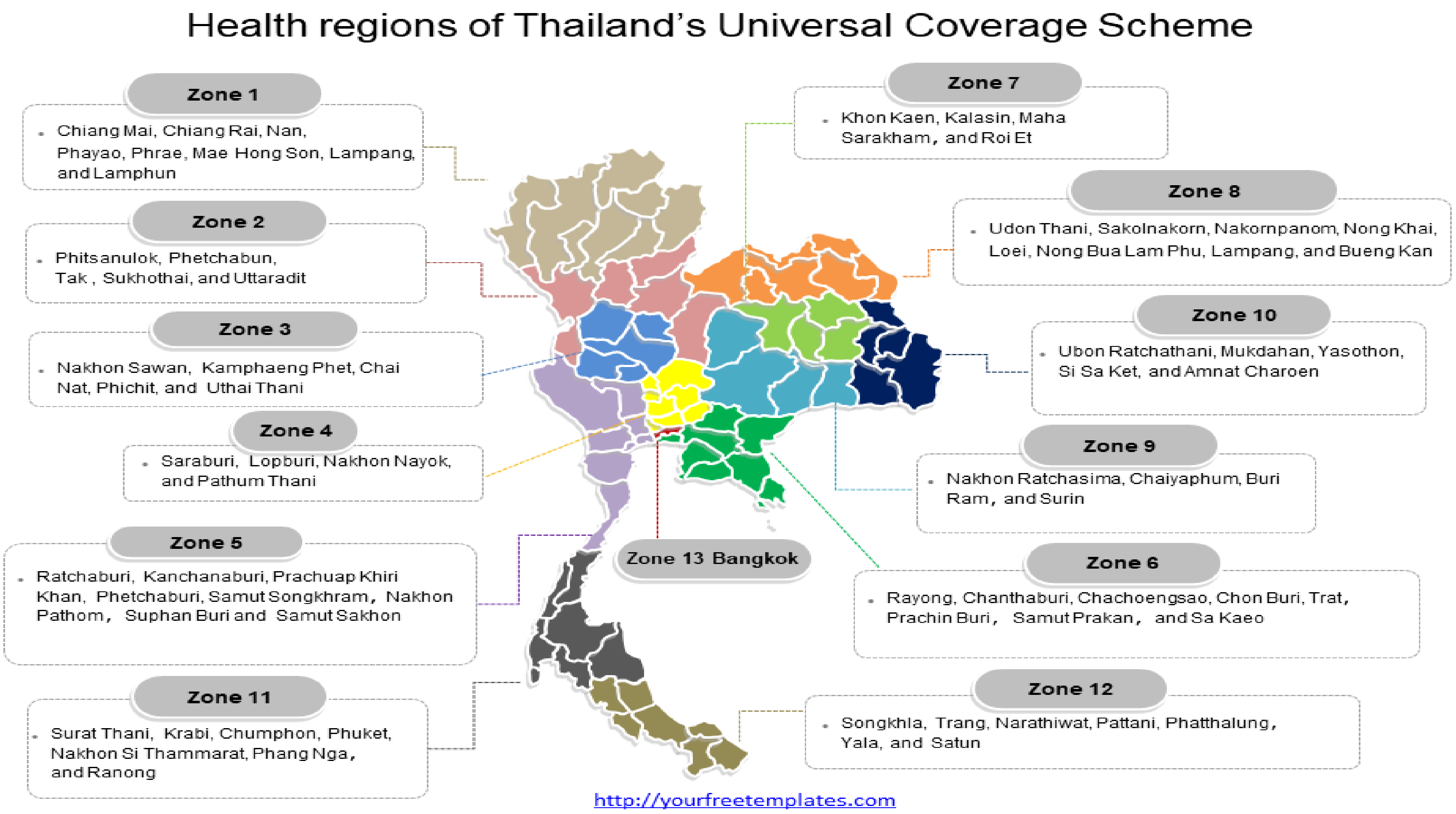

Supplementary figure 2 Flowchart of the data management process

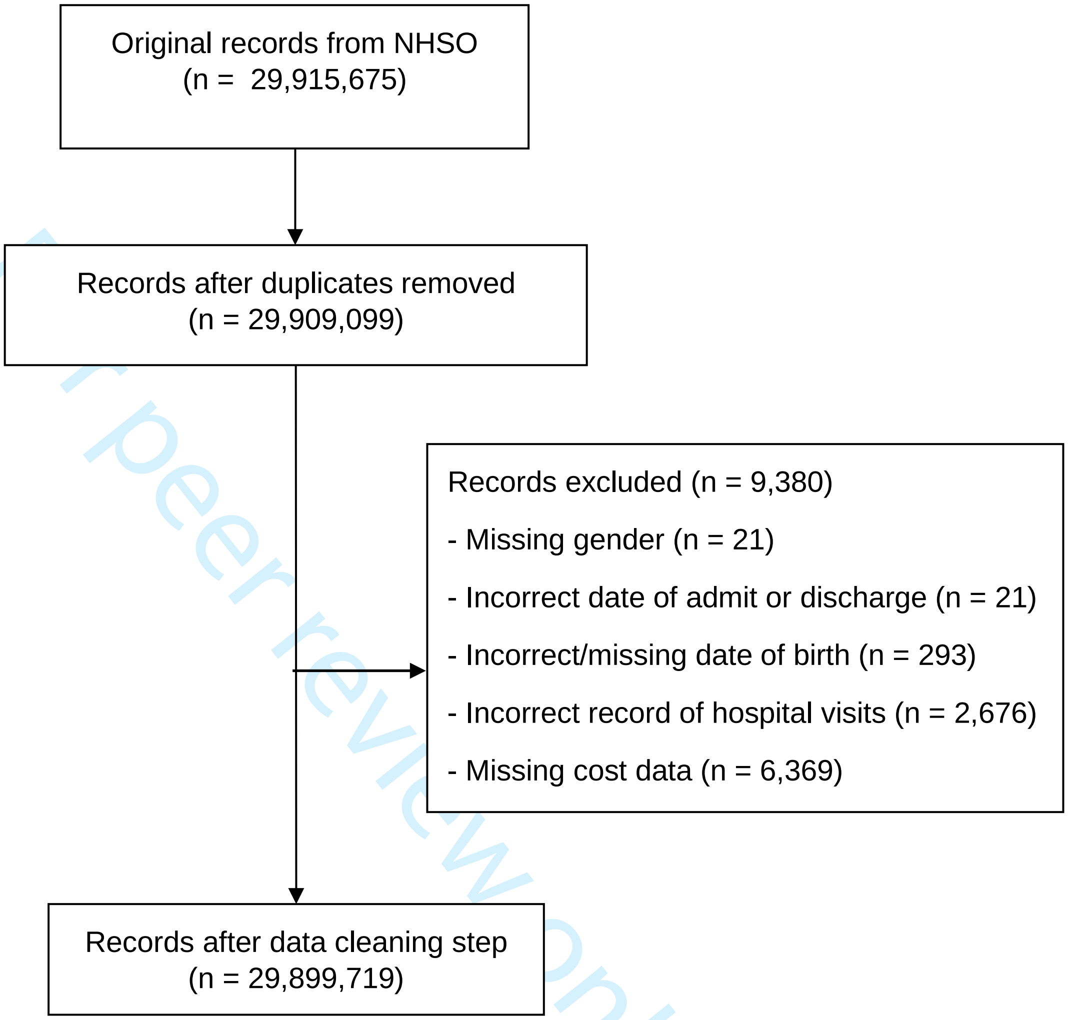

For peer review only - http://bmjopen.bmj.com/site/about/guidelines.xhtml

Supplementary table 1 Marginal effect of Predictors associated with high-cost users in UCS

	2013	2014	2015	2016	2017
	Prob (95%CI)	Prob (95%CI)	Prob (95%CI)	Prob (95%CI)	Prob (95%CI)
Age	0.00 (0.00, 0.00)	0.00 (0.00, 0.00)	0.00 (0.00, 0.00)	0.00 (0.00, 0.00)	0.00 (0.00, 0.00)
Gender (ref: female)	-0.01 (-0.01, -0.01)	-0.01 (-0.01, -0.01)	-0.01 (-0.01, -0.01)	-0.01 (-0.01, -0.01)	-0.01 (-0.01, -0.00)
Regional hospitals	0.05 (0.05, 0.05)	0.05 (0.05, 0.05)	0.05 (0.05, 0.05)	0.05 (0.05, 0.05)	0.05 (0.05, 0.05)
Hospital type (ref: other clinics)					
General hospitals, community hospitals, and other MOPH agencies	0.00 (0.00, 0.00)	0.01 (0.00, 0.01)	0.00 (0.00, 0.00)	0.00 (0.00, 0.00)	0.00 (0.00, 0.00)
Non MOPH agencies such as university hospitals	0.11 (0.10, 0.11)	0.13 (0.13, 0.14)	0.12 (0.12, 0.12)	0.11 (0.11, 0.12)	0.11 (0.11, 0.11)
Private hospitals	0.03 (0.03, 0.03)	0.07 (0.06, 0.07)	0.06 (0.06, 0.06)	0.05 (0.05, 0.06)	0.05 (0.05, 0.06)
Hospital zone: (ref: hospitals outside Bangkok)					
Bangkok	0.06 (0.06, 0.06)	0.09 (0.09, 0.09)	0.07 (0.07, 0.08)	0.07 (0.07, 0.08)	0.07 (0.06, 0.07)
Primary diagnosis					
Neoplasm	0.08 (0.08, 0.08)	0.08 (0.08, 0.08)	0.07 (0.07, 0.07)	0.07 (0.07, 0.08)	0.08 (0.07, 0.08)
Diseases of circulatory system	0.07 (0.07, 0.07)	0.07 (0.07, 0.08)	0.08 (0.08, 0.08)	0.08 (0.08, 0.08)	0.08 (0.08, 0.08)
Diseases of respiratory system	0.02 (0.02, 0.02)	0.01 (0.01, 0.01)	0.01 (0.01, 0.01)	0.01 (0.01, 0.01)	0.01 (0.01, 0.01)
Diseases of the digestive system	-0.00 (-0.00, -0.00)	-0.00 (-0.00, -0.00)	-0.00 (-0.00, -0.00)	-0.01 (-0.01, -0.01)	-0.00 (-0.00, -0.00)
Injury, or poisoning and certain other consequences of external causes	0.04 (0.04, 0.04)	0.05 (0.05, 0.05)	0.04 (0.04, 0.04)	0.04 (0.04, 0.04)	0.04 (0.04, 0.04)
Death	0.1 (0.10, 0.11)	0.08 (0.08, 0.09)	0.09 (0.08, 0.09)	0.07 (0.07, 0.08)	0.07 (0.06, 0.07)
Number of primary diagnoses	0.01 (0.01, 0.01)	0.01 (0.01, 0.01)	0.01 (0.01, 0.01)	0.01 (0.01, 0.01)	0.01 (0.01, 0.01)
Number of visits in a year	0.02 (0.02, 0.02)	0.02 (0.02, 0.02)	0.02 (0.02, 0.02)	0.02 (0.02, 0.02)	0.02 (0.02, 0.02)

Notes: Prob = Probability; 95% CI = 95% confidence interval

For peer review only

Supplementary table 2 Relative risk ratio of factors predicting being high-cost users and persistent high-cost users

Factors	Factors predicting being HCUs RRR (95% CI)	Factors predicting being persistent HCUs RRR (95% CI)
Age	1.04 (1.04, 1.04)	1.08 (1.08, 1.09)
Age*Age	0.99 (0.99, 0.99)	0.99 (0.99, 0.99)
Gender (ref: female)	0.69 (0.68, 0.70)	0.80 (0.77, 0.83)
Hospital type (ref: other clinics)		
Regional hospitals	2.83 (2.8, 2.86)	3.19 (3.12, 3.26)
General hospitals, community hospitals, and other MOPH agencies	1.09 (1.07, 1.10)	0.86 (0.83, 0.88)
Non MOPH agencies such as university hospitals	5.56 (5.46, 5.66)	10.2 (9.93, 10.5)
Private hospitals	2.29 (2.23, 2.35)	3.18 (3.04, 3.32)
Hospital zone: (ref: hospitals located outside Bangkok)		
Bangkok	4.40 (4.24, 4.57)	8.15 (7.63, 8.71)
Primary diagnosis		
Neoplasm	7.91 (7.54, 8.31)	43.5 (40.3, 47.0)
Diseases of circulatory system	7.24 (6.96, 7.53)	12.4 (11.5, 13.4)
Diseases of respiratory system	1.30 (1.27, 1.33)	2.21 (2.08, 2.35)
Diseases of the digestive system	0.73 (0.7, 0.75)	0.95 (0.89, 1.02)
Injury, or poisoning and certain other consequences of external causes	1.99 (1.93, 2.04)	2.78 (2.6, 2.97)
Comorbidity (Charlson comorbidity index)	1.12 (1.12, 1.13)	1.21 (1.20, 1.22)
Death	3.03 (2.93, 3.14)	3.63 (3.43, 3.85)
Number of primary diagnoses	1.71 (1.70, 1.71)	1.75 (1.74, 1.77)
Number of visits in a year	1.06 (1.06, 1.06)	1.16 (1.16, 1.17)
Neoplasm*Death	0.56 (0.54, 0.59)	0.60 (0.56, 0.64)
Diseases of circulatory system*Death	0.51 (0.49, 0.52)	0.55 (0.53, 0.59)
Diseases of respiratory system*Death	1.39 (1.34, 1.44)	1.79 (1.70, 1.88)
Diseases of the digestive system*Death	1.12 (1.08, 1.16)	1.22 (1.16, 1.29)
Injury, or poisoning and certain other consequences of external causes*Death	0.68 (0.65, 0.71)	0.77 (0.72, 0.82)
Neoplasm*Age	0.98 (0.98, 0.98)	0.96 (0.96, 0.97)
Diseases of circulatory system*Age	0.98 (0.98, 0.98)	0.98 (0.97, 0.98)
Diseases of respiratory system*Age	0.99 (0.99, 0.99)	0.99 (0.99, 0.99)
Diseases of the digestive system*Age	1.00 (1.00, 1.00)	0.99 (0.99, 0.99)
Injury, or poisoning and certain other consequences of external causes*Age	0.99 (0.99, 0.99)	0.99 (0.99, 0.99)

For peer review only

Factors	Factors predicting being HCUs RRR (95% CI)	Factors predicting being persistent HCUs RRR (95% CI)
Neoplasm*Gender	0.84 (0.82, 0.87)	0.69 (0.66, 0.72)
Diseases of circulatory system*Gender	1.01 (0.99, 1.03)	0.83 (0.80, 0.87)
Diseases of respiratory system*Gender	0.95 (0.93, 0.97)	0.82 (0.79, 0.86)
Diseases of the digestive system*Gender	1.06 (1.04, 1.09)	0.97 (0.93, 1.00)
Injury, or poisoning and certain other consequences of external causes*Gender	1.19 (1.16, 1.22)	1.23 (1.18, 1.28)
Hospital zone: Bangkok* Regional hospitals	0.55 (0.53, 0.57)	0.52 (0.49, 0.55)
Hospital zone: Bangkok* General hospitals, community hospitals, and other MOPH agencies	1.31 (1.27, 1.35)	1.56 (1.48, 1.65)
Hospital zone: Bangkok* Non MOPH agencies	0.28 (0.27, 0.29)	0.19 (0.18, 0.20)
Hospital zone: Bangkok* Private hospitals	0.42 (0.40, 0.44)	0.30 (0.28, 0.32)
Constant	0.00 (0.00, 0.00)	0.00 (0.00, 0.00)

Notes: 95% CI = 95% confidence interval; HCUs = high cost users; RRR = relative risk ratio.

For peer review only

STROBE 2007 (v4) Statement—Checklist of items that should be included in reports of *cross-sectional studies*

Section/Topic	Item #	Recommendation	Reported on page #
**Title and abstract**	1	(*a*) Indicate the study's design with a commonly used term in the title or the abstract	2
		(*b*) Provide in the abstract an informative and balanced summary of what was done and what was found	2
**Introduction**			
Background/rationale	2	Explain the scientific background and rationale for the investigation being reported	4-5
Objectives	3	State specific objectives, including any prespecified hypotheses	5
**Methods**			
Study design	4	Present key elements of study design early in the paper	5
Setting	5	Describe the setting, locations, and relevant dates, including periods of recruitment, exposure, follow-up, and data collection	5
Participants	6	(*a*) Give the eligibility criteria, and the sources and methods of selection of participants	5-7
Variables	7	Clearly define all outcomes, exposures, predictors, potential confounders, and effect modifiers. Give diagnostic criteria, if applicable	5-7
Data sources/ measurement	8*	For each variable of interest, give sources of data and details of methods of assessment (measurement). Describe comparability of assessment methods if there is more than one group	5-6
Bias	9	Describe any efforts to address potential sources of bias	5-7
Study size	10	Explain how the study size was arrived at	5-6
Quantitative variables	11	Explain how quantitative variables were handled in the analyses. If applicable, describe which groupings were chosen and why	5-6
Statistical methods	12	(*a*) Describe all statistical methods, including those used to control for confounding	6-7
		(*b*) Describe any methods used to examine subgroups and interactions	6-7
		(*c*) Explain how missing data were addressed	5
		(*d*) If applicable, describe analytical methods taking account of sampling strategy	N/A
		(*e*) Describe any sensitivity analyses	N/A
**Results**			

For peer review only

Participants	13*	(a) Report numbers of individuals at each stage of study—eg numbers potentially eligible, examined for eligibility, confirmed eligible, included in the study, completing follow-up, and analysed	7
		(b) Give reasons for non-participation at each stage	N/A
		(c) Consider use of a flow diagram	N/A
Descriptive data	14*	(a) Give characteristics of study participants (eg demographic, clinical, social) and information on exposures and potential confounders	7-10
		(b) Indicate number of participants with missing data for each variable of interest	7
Outcome data	15*	Report numbers of outcome events or summary measures	7-11
Main results	16	(*a*) Give unadjusted estimates and, if applicable, confounder-adjusted estimates and their precision (eg, 95% confidence interval). Make clear which confounders were adjusted for and why they were included	7-16
		(*b*) Report category boundaries when continuous variables were categorized	7-16
		(*c*) If relevant, consider translating estimates of relative risk into absolute risk for a meaningful time period	N/A
Other analyses	17	Report other analyses done—eg analyses of subgroups and interactions, and sensitivity analyses	N/A
**Discussion**			
Key results	18	Summarise key results with reference to study objectives	16-18
Limitations	19	Discuss limitations of the study, taking into account sources of potential bias or imprecision. Discuss both direction and magnitude of any potential bias	17-18
Interpretation	20	Give a cautious overall interpretation of results considering objectives, limitations, multiplicity of analyses, results from similar studies, and other relevant evidence	16-18
Generalisability	21	Discuss the generalisability (external validity) of the study results	16-18
**Other information**			
Funding	22	Give the source of funding and the role of the funders for the present study and, if applicable, for the original study on which the present article is based	19

*Give information separately for cases and controls in case-control studies and, if applicable, for exposed and unexposed groups in cohort and cross-sectional studies.

Note: An Explanation and Elaboration article discusses each checklist item and gives methodological background and published examples of transparent reporting. The STROBE checklist is best used in conjunction with this article (freely available on the Web sites of PLoS Medicine at http://www.plosmedicine.org/, Annals of Internal Medicine at http://www.annals.org/, and Epidemiology at http://www.epidem.com/). Information on the STROBE Initiative is available at www.strobe-statement.org.

For peer review only

BMJ Open

Retrospective secondary data analysis to identify high-cost users in inpatient department of hospitals in Thailand, a middle-income country with universal healthcare coverage

Journal:	*BMJ Open*
Manuscript ID	bmjopen-2020-047330.R2
Article Type:	Original research
Date Submitted by the Author:	30-Jun-2021
Complete List of Authors:	Rattanavipapong, Waranya; Health Intervention and Technology Assessment Program, Health<br>Yi, Wang; National University of Singapore<br>Butchon, Rukmanee; Health Intervention and Technology Assessment Program, Health<br>Kittiratchakool, Nitichen; Health Intervention and Technology Assessment Program<br>Thammatacharee , Jadej; National Health Security Office<br>Teerawattananon, Yot; Health Intervention and Technology Assessment Program, Health; National University of Singapore<br>Isaranuwatchai, Wanrudee; Health Intervention and Technology Assessment Program, Health
**Primary Subject Heading**:	Health policy
Secondary Subject Heading:	Evidence based practice
Keywords:	HEALTH ECONOMICS, Health policy < HEALTH SERVICES ADMINISTRATION & MANAGEMENT, PUBLIC HEALTH, STATISTICS & RESEARCH METHODS

SCHOLARONE™
Manuscripts

I, the Submitting Author has the right to grant and does grant on behalf of all authors of the Work (as defined in the below author licence), an exclusive licence and/or a non-exclusive licence for contributions from authors who are: i) UK Crown employees; ii) where BMJ has agreed a CC-BY licence shall apply, and/or iii) in accordance with the terms applicable for US Federal Government officers or employees acting as part of their official duties; on a worldwide, perpetual, irrevocable, royalty-free basis to BMJ Publishing Group Ltd ("BMJ") its licensees and where the relevant Journal is co-owned by BMJ to the co-owners of the Journal, to publish the Work in this journal and any other BMJ products and to exploit all rights, as set out in our licence.

The Submitting Author accepts and understands that any supply made under these terms is made by BMJ to the Submitting Author unless you are acting as an employee on behalf of your employer or a postgraduate student of an affiliated institution which is paying any applicable article publishing charge ("APC") for Open Access articles. Where the Submitting Author wishes to make the Work available on an Open Access basis (and intends to pay the relevant APC), the terms of reuse of such Open Access shall be governed by a Creative Commons licence – details of these licences and which Creative Commons licence will apply to this Work are set out in our licence referred to above.

Other than as permitted in any relevant BMJ Author's Self Archiving Policies, I confirm this Work has not been accepted for publication elsewhere, is not being considered for publication elsewhere and does not duplicate material already published. I confirm all authors consent to publication of this Work and authorise the granting of this licence.

For peer review only

Retrospective secondary data analysis to identify high-cost users in inpatient department of hospitals in Thailand, a middle-income country with universal healthcare coverage

Correspondence to Wanrudee Isaranuwatchai; 6th Floor, 6th Building, Department of Health, Ministry of Public Health, Tiwanon Rd., Muang, Nonthaburi 11000, Thailand; wanrudee.i@hitap.net; +662-590-4549, +662-590-4374, +662-590-4375

Authors and Institutions

Waranya Rattanavipapong[1], Wang Yi[2], Rukmanee Butchon[1], Nitichen Kittiratchakool[1], Jadej Thammatacharee[3], Yot Teerawattananon[1], Wanrudee Isaranuwatchai[1,4]

Author affiliations

Waranya Rattanavipapong[1], Wang Yi[2], Rukmanee Butchon[1], Nitichen Kittiratchakool[1], Jadej Thammatacharee[3], Yot Teerawattananon[1,2], Wanrudee Isaranuwatchai[1,4]

[1]Health Intervention and Technology Assessment Program, Nonthaburi, Thailand

[2]Health Intervention and Policy Evaluation Research, Saw Swee Hock School of Public Health, National University of Singapore, Singapore

[3]National Health Security Office, Thailand

[4]Institute or Health Policy, Management and Evaluation, University of Toronto, Canada

Word count: 4,198

For peer review only

ABSTRACT

Objectives

The study aims to identify high-cost users (HCUs) in inpatient department of hospitals in Thailand including their common characteristics, patterns of healthcare utilization and expenditure compared to low-cost users, and to explore potential factors associated with HCUs so the healthcare system can be prepared to support the HCUs including those who have increased chance of becoming HCUs.

Design and setting

A retrospective secondary data analysis using hospitalization data from Thailand's universal coverage scheme (UCS) obtained from the National Health Security Office over a 5-year period from October 2014 to September 2019 (fiscal year 2014-2018).

Participants

Study participants included Thai citizens who had at least one inpatient admission to hospitals under the UCS over the study period.

Results

Over the 5-year period, the top 5% of hospitalized population (or HCUs) consumed almost 50% of the health expenditure each year. HCUs were more likely to have longer hospital stays, have a higher annual number of visits, and be admitted to multiple hospitals each year when compared to the low-cost users (the bottom 50% of hospitalized population). The study further reported that the chance of becoming HCUs is associated with several factors such as increasing age, being male, having comorbidity, and being admitted to hospitals in Bangkok.

Conclusions

This study confirmed that the HCUs phenomenon existed in Thailand, where a majority of inpatient care spending under is concentrated in the top 5% of hospitalized population. The study findings call attention to potential initiatives that can help monitor the magnitude and trend of HCUs and develop policies to prevent HCUs.

For peer review only

Strengths and limitations of this study

- This study is one of the first studies to use real-world administrative hospitalization data from Thailand's universal coverage scheme to explore the high-cost users (HCUs) phenomenon, and to identify characteristics of HCUs.
- The study represents an example of how existing routinely collected real-world data can be used to potentially improve care and health service and to help address policy-relevant questions.
- The analysis was limited by data availability, and future work could explore beyond hospitalization data to consider other types of health services (e.g. outpatient services), and could include other potential confounders (e.g. education and income).
- Significant work were conducted to clean and manage the data which highlighted the importance of data quality especially in countries who may have not used real-world data to their full potential.

Keywords: high-cost users, high-cost, health care utilization, hospitalization, administrative databases, real-world data, Thailand

For peer review only

INTRODUCTION

There is an increasing emphasis on the sustainability of healthcare systems for many reasons, including the availability of new costly health technologies, the greater demand for healthcare by an aging population, and the government's commitment to achieve universal health coverage (UHC) under limited resources.[1 2] As such, several solutions have been attempted to enhance the efficiency of healthcare systems around the world.[3-6] These potential solutions include the prioritisation of health problems and associated interventions based on factors such as burden of disease and cost-effectiveness evidence,[7 8] identifying low-value health services that can be omitted without adversely affecting patient outcomes,[9 10] and promoting the use of preventive measures to avoid patients' development of chronic conditions.[11 12]

Based on a literature review of the high-cost users (HCUs) phenomenon in several countries, for example, Japan, the United States, Australia, and Canada,[13-18] HCUs are generally defined as the top 5% of patients with the highest health expenditure in a given year, while low-cost users (LCUs) are defined as the bottom 50% of patients with the lowest healthcare costs.[15-17] Although the aforementioned evidence is only from high-income countries, this phenomenon is relevant and important for low- and middle-income countries (LMICs) where health resources are limited. Additionally, there are studies on health service utilization in LMICs focusing on general patterns of utilization and certain high-cost interventions;[19-21] however they do not focus on HCUs. Therefore, a better understanding of the HCUs in Thailand (an upper middle-income country (UMIC)[22] regarding HCUs' characteristics and patterns of healthcare utilization may help the country (including other LMICs with similar country profiles) to identify potential HCUs and develop more tailored and appropriate interventions to improve the management and financing of these patients.

Thailand has been classified as a UMIC since 2011[22] and has a rapidly growing aging population. The proportion of the population aged 60 years or over increased from 12% in 2011 to 18% in 2020,[23] and is projected to increase further to 25% in 2030.[24] Leading causes of burdens of disease (cause of death and disability) in the Thai population include injury, cardiovascular disease, cancer, and diabetes mellitus.[25] These population characteristics, to some extent, align with potential factors associated with HCUs identified in the literature from high-income countries such as being older and having comorbidities.[16 18]

Although the Thai government invests approximately 13.3% of the government budget on public health,[22] the Universal Coverage Scheme (UCS) budget has increased annually during the past decade and at a faster rate than the annual gross domestic product (GDP) growth, which is directly affecting the sustainability of the healthcare system.[22 26] The three

For peer review only

main health insurance programs employed to cover nearly the full Thai population include the Civil Servant Medical Benefit Scheme (CSMBS) for government employees and their dependents, the Social Security Scheme (SSS) for formal private sector employees, and the UCS for the remaining Thai citizens.[22] The UCS, which is managed by the National Health Security Office (NHSO), covers approximately 80% of the total population who are not eligible for the CSMBS and SSS, including low-income or unemployed citizens. Characteristics of the aforementioned three health insurance programs in Thailand are published elsewhere.[22]

The UCS provides a comprehensive benefits package for curative, rehabilitation, and health promotion and prevention services.[27] Most of the UCS budget is allocated to outpatient and inpatient services. Payment for outpatient care is generally based on capitation while inpatient care payment is based on diagnosis-related groups (DRGs).[28 29] There is also an additional budget for specific high-cost interventions or medications.[30] Nearly half of the UCS expenditure (e.g. 42% in 2011) is spent on inpatient care.[26]

Research on the HCUs phenomenon under Thailand's biggest public health insurance scheme, the UCS, is essential for identifying potential measures of which some of them are deemed 'preventable' spending. For example, spending on treatment for diabetic nephropathy that would have been avoidable if diabetic screening and early treatments had taken place. Moreover, treatment costs of lung cancer could have been avoided if specific public health interventions were available to reduce tobacco consumption.[31 32]

This study aims to identify demographic characteristics of HCUs; understand the common characteristics and patterns of healthcare utilization and expenditure in hospitals among HCUs as compared to LCUs; and determine potential factors associated with HCUs. This study is the first of its kind in UMICs and its findings have the potential to be used by healthcare managers and policy-makers in relevant settings (including LMICs) to avert avoidable HCUs in hospitals and reallocate these limited resources for other cost-effective options.

METHODS

Study population, setting, and data

This study was a retrospective secondary data analysis which examined inpatient department (or hospitalization) data from Thailand's UCS obtained from the NHSO over a 5-year period from 2014 to 2018. NHSO defines inpatient as a patient who was formally admitted into a hospital for treatment and who stayed for a minimum six hours. Using this

For peer review only - http://bmjopen.bmj.com/site/about/guidelines.xhtml

administrative hospitalization database, the study population included Thai citizens of all ages who were hospitalized under the UCS health insurance scheme and discharged between October 1st, 2014, and September 30th, 2019, according to the Thai government's fiscal year. A unique national identification (ID) number is registered for each Thai citizen in the system. Thai citizens with a national ID who had at least one hospital admission during the study period were included in the study dataset. The subjects' unique IDs were masked using a private key by the data holder before the dataset was shared with the research team.

All data were anonymised and de-identified. Records with encrypted identities were checked, and duplicated records or those with incomplete data were excluded from analysis. To ensure that the data were accurate and consistent, conflicting demographic information about date of birth, gender, and admission and discharge dates were investigated and resolved. Conflicting records were removed (with the final number of records being 29,899,719).

Variables considered in the analysis comprised of: gender; date of birth; primary diagnosis (using ICD-10 codes); admission and discharge dates; discharge status (full recovery, improved, not improved, normal delivery, undelivered, normal child discharged with mother, separation of mother and baby, stillbirth, and death); types of hospitals (i.e. from the lowest level being clinics and community hospitals to the higher level, namely private hospitals, general hospitals, regional hospitals, and other hospitals not under the Ministry of Public Health, such as university hospitals); health regions as shown in the Supplementary figure 1; Charlson comorbidity index;[33 34] and reimbursement cost (which refers to the amount estimated to be paid by NHSO and reported in the hospitalization database).

This study received ethical approval from Institute for the Development of Human Research Protections, Ministry of Public Health, Thailand. This study used an existing administrative database (with de-identified individual-level hospitalization data); therefore, patients had no direct involvement or risk and no consent was required.

Patient and public involvement

No patient involved.

Analysis

Using the NHSO's hospitalization database, HCUs and persistent HCUs were examined using descriptive and multivariable regression analysis. HCUs and LCUs were defined by the percentile of hospitalization costs accrued. HCUs were individuals who had incurred

For peer review only - http://bmjopen.bmj.com/site/about/guidelines.xhtml

costs higher than 95% of all users (the top 5%). LCUs were patients in the lowest cost group (the bottom 50%). The dataset was treated as repeated cross-sectional when examining HCUs and the analysis was conducted separately by year based on patients' discharge dates using similar methods as seen in the literature.[16 18] For example, if patients were admitted to a hospital in one fiscal year but discharged in the following fiscal year, their costs would be included since the admitted fiscal year. We define persistent HCUs as those who were identified as HCUs for more than one year; further explanation is provided below. When examining persistent HCUs, a panel dataset was constructed by tracking the individuals' statuses (of being an HCU) over multiple years.

Every hospital admission was tracked within each fiscal year (October 1st to September 30th). To address the study aims, first, we sought to determine the concentration of inpatient department expenditures to explore the phenomenon of HCUs in Thailand. Total costs associated with all visits within each fiscal year for each patient (i.e. estimated reimbursement costs) were calculated and individuals were sorted into 5 groups according to the percentile of costs: 1) above the 99th percentile; 2) between the 95th and the 98th percentile; 3) between the 90th and 94th percentiles; 4) between the 50th and 89th percentiles; and 5) below the 50th percentile. Patients may have more than one hospital admission; therefore, we summed up all admissions per patients within each year to calculate the annual hospitalization cost per patient as used in the HCU grouping.

Next, we examined the differences in demographic characteristics, costs, and comorbidities of HCUs as compared to LCUs. Descriptive characteristics of each group were analysed by age, gender, and identified main diagnosis based on ICD-10 codes. We also reported healthcare utilization and expenditure patterns between HCUs and LCUs, specifically number of visits, length of stay (LOS), types of hospitals visited, and annual cost per patient.

Multivariable logistic regression was conducted to identify potential factors associated with HCUs. The outcome variable was patient HCU status (yes/no) and the exploratory variables included age, gender, number of visits, types and health regions of hospitals visited, main diagnoses, and comorbidities (measured as Charlson comorbidity index, and the presence of the top five diagnoses with highest total cost to the public healthcare payer for the study population namely neoplasms, circulatory diseases, respiratory diseases, digestive problems, and injuries). Interaction terms were also included to examine the heterogeneous effects on becoming a potential HCU; specifically, interactions between death, age, or gender and the top five primary diagnoses (neoplasms, circulatory diseases, respiratory diseases, digestive problems, and injuries) including between types of hospital and health regions were considered.

For peer review only - http://bmjopen.bmj.com/site/about/guidelines.xhtml

To identify the persistent HCUs, only the data between 2015 and 2017 were examined due to the completeness of the data. To construct a panel dataset, patients who were presented in the dataset in 2015 were used for the analysis. Any patients who died were excluded as they could not become persistent HCUs. For the remaining patients, we examined their HCU status in the years 2016 and 2017 to determine whether they were persistent HCUs. They were then grouped into three levels: 0 (not an HCU for any of the three years); 1 (being an HCU for only one year); and 2 (being an HCU for at least two years). Group 2 was defined as persistent HCUs. A multinomial logistic regression was applied to examine the potential factors associated with persistent HCUs.

All analyses were conducted using Stata Statistical Software (Release 15, College Station, TX). Chi-square tests and t-tests were used to test for significance of differences at p-value < 0.05.

RESULTS

A total of 29,899,719 admissions (14,464,180 unique patients) between fiscal years 2014–2018 were analysed. On average, each year had approximately 5.9 million admissions (about 3.9 million unique patients). Additional details on the data management process can be found in Supplementary figure 2.

The hospitalization data of UCS have shown that a small proportion of patients (the top 5% of hospitalized population or HCUs) consumed almost half of the healthcare expenditure; this trend was observed over a 5-year period as presented in **Figure 1**. During this period, patients in the top 1% and 5% cost groups accounted for 16—18% and 45—48% of total spending, respectively. Furthermore, a separate analysis showed that HCUs had higher annual mortality rates compared to LCUs. Among HCUs, mortality ranged from 19%—21% over 5 years, compared with 0.1%—1.3% in LCUs.

Figure 1 Distribution of total hospitalization's expenditures across cost percentiles

To facilitate the understanding of the HCU phenomenon, the profile of HCUs was studied. Generally, the average age of HCUs was 55—56 years old, where more than half (50-54% each year) were in the age group of ≥60. In Thailand, about 18% of the population were older adults (≥60). A approximately 55% of this sample were male, while approximately 49% were male in the general Thai population.[23] **Table 1** provides the descriptive characteristics of HCUs as compared with LCUs.

For peer review only - http://bmjopen.bmj.com/site/about/guidelines.xhtml

Table 1 Demographic characteristics and patterns of utilization and expenditure of HCUs and LCUs from 2014 to 2018*,**

Characteristic	2014		2015		2016		2017		2018	
	HCUs	LCUs	HCUs	LCUs	HCUs	LCUs	HCUs	LCUs	HCUs	LCUs
Number of patients	234,214	1,913,712	235,954	1,964,952	243,850	1,991,451	239,180	1,953,317	243,951	1,992,271
Number of admissions	864,787	1,991,875	863,308	2,054,265	899,142	2,090,249	926,434	2,053,590	951,527	2,096,159
Mean age in years (SD)	54.6 (22.6)	22.6 (25.0)	54.5 (22.4)	24.1 (25.5)	55.4 (22.3)	23.8 (25.4)	55.6 (22.3)	24.8 (25.9)	56.1 (22.1)	25.2 (26.1)
Male (%)	55%	44%	55%	44%	55%	45%	55%	45%	55%	45%
Average length of stay in days (SD)	11.3 (21.2)	2.5 (2.8)	11.3 (22.0)	2.6 (2.1)	10.9 (21.6)	2.6 (2.9)	10.8 (19.1)	2.6 (2.6)	10.4 (18.8)	2.6 (2.3)
Average number of visits (SD)	3.7 (3.3)	1.0 (0.2)	3.7 (3.3)	1.0 (0.2)	3.7 (3.4)	1.0 (0.2)	3.9 (3.5)	1.0 (0.2)	3.9 (3.5)	1.0 (0.2)
Number of hospitals visited (% of admissions)										
1	49.4%	99.0%	49.7%	99.0%	49.8%	99.0%	48.5%	99.0%	48.4%	99.0%
2	41.3%	1.0%	40.4%	1.0%	40.7%	1.0%	41.6%	1.0%	41.8%	1.0%
3	8.1%	-	8.6%	-	8.3%	-	8.7%	-	8.6%	-
>3	1.2%	-	1.3%	-	1.2%	-	1.2%	-	1.2%	-
Types of hospitals visited (% of admissions)										
Regional hospitals	35.5%	16.2%	33.6%	16.2%	34.9%	15.8%	34.0%	16.3%	34.3%	16.0%
General hospitals	21.2%	21.8%	20.0%	22.0%	20.9%	21.7%	21.5%	22.0%	22.4%	21.8%
Community hospitals	20.1%	53.3%	18.7%	54.2%	19.6%	54.1%	20.7%	53.3%	21.4%	53.7%
Others	23.2%	8.7%	27.7%	7.6%	24.6%	8.4%	23.8%	8.4%	21.9%	8.5%

For peer review only - http://bmjopen.bmj.com/site/about/guidelines.xhtml

Characteristic	2014		2015		2016		2017		2018	
	HCUs	LCUs	HCUs	LCUs	HCUs	LCUs	HCUs	LCUs	HCUs	LCUs
Average total cost per patient in baht (SD)	113,500 (75,600)	2,800 (1,060)	98,000 (71,200)	2,200 (810)	109,200 (76,300)	2,500 (950)	111,100 (74,500)	2,700 (1,040)	115,000 (77,400)	2,900 (1,120)

HCUs = high-cost users; LCU = low cost users; SD = standard deviation.

*The number (except number of patients and admissions) presented in table was rounded to the nearest whole number.

**All variables are significant (p-value < 0.05).

For peer review only

Over the study period, HCUs were older and included more men than compared to LCUs. The mean age ranged between 55—56 years and 23—25 years among HCUs and LCUs, respectively. HCUs had longer lengths of stay and used more healthcare services (higher frequency of hospital visits and higher number of visited hospitals). Moreover, HCUs visited high level hospitals (e.g. regional and university hospitals), while LCUs visited predominantly community hospitals. The pattern of the results were similar across the 5-year period.

Regarding the expenditure pattern, HCUs spent considerably more per year than LCUs in the index year. Over 5 years, the average annual spending for HCUs was between 107,600 (± 107,600) to 130,700 (± 102,500) Baht, compared to 2,300 (± 820) to 2,900 (± 1,130) Baht for LCUs, where 1 US dollar is approximately 32 Thai baht. Details of the expenditure pattern are presented in **Table 1**.

The most common and most expensive clinical conditions between HCUs and LCUs were examined and found to differ. **Table 2** confirms that the expenditures were spent on different diagnoses. Among HCUs, the most frequent diagnoses included neoplasms and diseases of the circulatory system and respiratory system which consumed more than half of annual expenditure. Among LCUs, the most frequent diagnoses were broader and included infectious and parasitic diseases, factors influencing health status and contact with health services (e.g. admissions for investigation), and diseases of the respiratory system; almost half of LCUs' annual expenditure was spent on these conditions.

For peer review only - http://bmjopen.bmj.com/site/about/guidelines.xhtml

1 **Table 2 The top five primary diagnoses of HCUs and LCUs over a 5-year study period***

High-cost users (HCUs)			Low cost users (LCUs)		
**Conditions**	**% of total admissions**	**% of total expenditures**	**Conditions**	**% of total admissions**	**% of total expenditures**
Neoplasms	22-23	17-20	Certain infectious and parasitic diseases	17-19	15-17
Diseases of circulatory system	17-20	25-30	Factors influencing health status and contact with health services	14-17	9-12
Diseases of respiratory system	13-14	10-12	Diseases of the respiratory system	11-14	13-17
Diseases of genitourinary system	7-8	3	Pregnancy, childbirth, puerperium	10-12	11-14
Diseases of the digestive system	7	5	Injury, poisoning and certain other consequences of external causes	9	8-9

2 HCUs = high-cost users; LCU = low cost users.

3 *The findings reported covered the 5-year study period so the lowest percentage and highest percentage during this period were presented.

For peer review only - http://bmjopen.bmj.com/site/about/guidelines.xhtml

Regression models were used to examine the potential factors associated with being an HCU (**Table 3**). The findings suggest that age, gender, types and zones of hospital admissions, disease and multimorbidity, number of visits, and mortality were associated with becoming an HCU (although the relative contribution of these factors towards the expenditure varied). Most of the interaction terms were statistically significant, suggesting that the impact of these factors were heterogenous among different groups.

For peer review only

Table 3 Factors associated with high-cost users in UCS*

Factors Associated with Being High-cost User	2014	2015	2016	2017	2018
	OR (95%CI)	OR (95%CI)	OR (95%CI)	OR (95%CI)	OR (95%CI)
Age	1.03 (1.03, 1.03)	1.03 (1.03, 1.04)	1.03 (1.03, 1.04)	1.03 (1.03, 1.03)	1.03 (1.03, 1.03)
Age*Age	0.99 (0.99, 0.99)	0.99 (0.99, 0.99)	0.99 (0.99, 0.99)	0.99 (0.99, 0.99)	0.99 (0.99, 0.99)
Gender (ref: female)	0.73 (0.71, 0.74)	0.77 (0.75, 0.78)	0.79 (0.78, 0.80)	0.79 (0.78, 0.80)	0.82 (0.81, 0.83)
Hospital type (ref: other clinics)					
Regional hospitals	3.42 (3.37, 3.47)	3.45 (3.40, 3.50)	3.47 (3.42, 3.52)	3.24 (3.19, 3.29)	3.16 (3.11, 3.20)
General hospitals, community hospitals, and other MOPH agencies	1.01 (1.00, 1.03)[+]	1.03 (1.02, 1.05)	1.00 (0.98, 1.02)[+]	1.02 (1.00, 1.03)	1.02 (1.01, 1.04)
Non MOPH agencies such as university hospitals	6.60 (6.45, 6.75)	9.06 (8.86, 9.26)	7.51 (7.35, 7.69)	7.21 (7.04, 7.37)	6.42 (6.28, 6.57)
Private hospitals	2.10 (2.03, 2.18)	4.06 (3.94, 4.18)	3.46 (3.35, 3.56)	3.28 (3.17, 3.40)	3.15 (3.04, 3.26)
Hospital zone: (ref: hospitals outside Bangkok)					
Bangkok	3.12 (2.97, 3.29)	4.73 (4.51, 4.97)	3.8 (3.61, 3.99)	3.92 (3.73, 4.12)	3.25 (3.09, 3.42)
Primary diagnosis					
Neoplasms	9.30 (8.78, 9.85)	8.96 (8.46, 9.49)	9.09 (8.59, 9.62)	8.77 (8.28, 9.28)	10.0 (9.49, 10.6)
Diseases of circulatory system	9.48 (9.01, 9.97)	9.6 (9.13, 10.0)	10.5 (10.0, 11.1)	11.0 (10.4, 11.5)	10.9 (10.4, 11.5)
Diseases of respiratory system	1.01 (0.98, 1.05)	1.00 (0.96, 1.03)	1.04 (1.00, 1.07)	0.99 (0.96, 1.02)[+]	1.11 (1.07, 1.15)
Diseases of the digestive system	0.62 (0.59, 0.65)	0.61 (0.58, 0.65)	0.62 (0.59, 0.65)	0.62 (0.59, 0.65)	0.61 (0.58, 0.64)
Injury, or poisoning and certain other consequences of external causes	2.41 (2.33, 2.50)	2.45 (2.37, 2.54)	2.37 (2.29, 2.46)	2.1 (2.02, 2.18)	2.26 (2.17, 2.34)
Comorbidity (Charlson comorbidity index)	1.07 (1.07, 1.08)	1.05 (1.04, 1.05)	1.05 (1.04, 1.05)	1.03 (1.03, 1.04)	1.02 (1.01, 1.02)
Death	6.53 (6.34, 6.73)	5.42 (5.26, 5.58)	5.43 (5.28, 5.59)	4.73 (4.59, 4.88)	4.06 (3.94, 4.19)
Number of primary diagnoses	1.20 (1.19, 1.21)	1.25 (1.24, 1.26)	1.24 (1.23, 1.25)	1.27 (1.26, 1.28)	1.28 (1.27, 1.29)

For peer review only

For peer review only - http://bmjopen.bmj.com/site/about/guidelines.xhtml

Factors Associated with Being High-cost User	2014	2015	2016	2017	2018
	OR (95%CI)	OR (95%CI)	OR (95%CI)	OR (95%CI)	OR (95%CI)
Number of visits in a year	1.65 (1.64, 1.66)	1.57 (1.56, 1.58)	1.55 (1.54, 1.56)	1.56 (1.56, 1.57)	1.54 (1.54, 1.55)
Neoplasms*Death	0.31 (0.30, 0.33)	0.38 (0.36, 0.39)	0.39 (0.37, 0.41)	0.39 (0.38, 0.41)	0.43 (0.41, 0.45)
Diseases of circulatory system*Death	0.36 (0.35, 0.37)	0.40 (0.39, 0.42)	0.35 (0.33, 0.36)	0.39 (0.38, 0.41)	0.44 (0.42, 0.46)
Diseases of respiratory system*Death	2.00 (1.92, 2.08)	1.59 (1.53, 1.66)	1.75 (1.68, 1.81)	1.50 (1.45, 1.56)	1.44 (1.39, 1.50)
Diseases of the digestive system*Death	1.09 (1.04, 1.14)	1.2 (1.15, 1.26)	1.12 (1.07, 1.18)	1.22 (1.16, 1.28)	1.38 (1.31, 1.44)
Injury, or poisoning and certain other consequences of external causes*Death	0.75 (0.71, 0.79)	0.64 (0.6, 0.67)	0.63 (0.59, 0.66)	0.71 (0.67, 0.75)	0.76 (0.72, 0.80)
Neoplasms*Age	0.99 (0.99, 0.99)	0.99 (0.99, 0.99)	0.99 (0.99, 0.99)	0.99 (0.99, 0.99)	0.99 (0.98, 0.99)
Diseases of circulatory system*Age	0.98 (0.98, 0.98)	0.98 (0.98, 0.98)	0.98 (0.98, 0.98)	0.98 (0.98, 0.98)	0.98 (0.98, 0.98)
Diseases of respiratory system*Age	1.01 (1.01, 1.01)	1.00 (1.00, 1.00)	1.00 (1.00, 1.01)	1.00 (1.00, 1.00)	1.00 (1.00, 1.00)
Diseases of the digestive system*Age	1.01 (1.01, 1.01)	1.00 (1.00, 1.01)	1.01 (1.00, 1.01)	1.00 (1.00, 1.01)	1.00 (1.00, 1.01)
Injury, or poisoning and certain other consequences of external causes*Age	0.99 (0.99, 1.00)$^{+}$	1.00 (1.00, 1.00)	1.00 (1.00, 1.00)	1.00 (1.00, 1.00)	1.00 (0.99, 1.00)$^{+}$
Neoplasms*Gender	0.84 (0.82, 0.87)	0.75 (0.72, 0.77)	0.71 (0.69, 0.74)	0.71 (0.69, 0.73)	0.66 (0.64, 0.68)
Diseases of circulatory system*Gender	1.00 (0.98, 1.03)$^{+}$	0.9 (0.88, 0.92)	0.91 (0.89, 0.93)	0.90 (0.88, 0.92)	0.88 (0.86, 0.91)
Diseases of respiratory system*Gender	0.98 (0.95, 1.01)$^{+}$	0.98 (0.95, 1.01)$^{+}$	0.94 (0.91, 0.96)	0.93 (0.91, 0.96)	0.91 (0.89, 0.93)
Diseases of the digestive system*Gender	1.07 (1.04, 1.11)	1.06 (1.03, 1.1)	1.02 (0.99, 1.05)$^{+}$	1.03 (1.00, 1.07)	1.01 (0.98, 1.05)$^{+}$
Injury, or poisoning and certain other consequences of external causes*Gender	1.17 (1.13, 1.21)	1.2 (1.16, 1.24)	1.21 (1.18, 1.25)	1.23 (1.19, 1.26)	1.22 (1.19, 1.26)
Hospital zone: Bangkok* Regional hospitals	0.62 (0.59, 0.66)	0.57 (0.54, 0.61)	0.59 (0.56, 0.63)	0.59 (0.56, 0.63)	0.66 (0.62, 0.69)
Hospital zone: Bangkok* General hospitals, community hospitals, and other MOPH agencies	1.79 (1.71, 1.88)	1.88 (1.79, 1.96)	1.83 (1.75, 1.92)	1.69 (1.61, 1.76)	1.7 (1.62, 1.78)
Hospital zone: Bangkok* Non MOPH agencies	0.30 (0.29, 0.32)	0.23 (0.22, 0.24)	0.26 (0.24, 0.27)	0.25 (0.24, 0.27)	0.3 (0.29, 0.32)

For peer review only - http://bmjopen.bmj.com/site/about/guidelines.xhtml

Factors Associated with Being High-cost User	2014	2015	2016	2017	2018
	OR (95%CI)	OR (95%CI)	OR (95%CI)	OR (95%CI)	OR (95%CI)
Hospital zone: Bangkok* Private hospitals	0.54 (0.51, 0.58)	0.28 (0.27, 0.30)	0.34 (0.32, 0.36)	0.33 (0.31, 0.35)	0.32 (0.30, 0.34)
Constant	0.00 (0.00, 0.00)	0.00 (0.00, 0.00)	0.00 (0.00, 0.00)	0.00 (0.00, 0.00)	0.00 (0.00, 0.00)

95% CI = 95% confidence interval; OR = odds ratio.

[+]P-value not statistically significant (>0.05).

For peer review only

For peer review only - http://bmjopen.bmj.com/site/about/guidelines.xhtml

On average, HCUs were more likely to be: 1) older; 2) male; 3) admitted to higher level hospitals (e.g. regional and university hospitals); 4) admitted to hospitals in Bangkok; 5) patient with a primary malignant tumour, diseases of the circulatory system, injury, poisoning and certain other consequences of external causes; 6) patients with multiple diagnosis; 7) patients with other comorbidities; 8) patients with more than one hospitalization within a year; and 9) patients who died before their discharge. The marginal effects are presented in the Supplementary table 1, showing that the results, in term of the probability of being an HCU for average individuals, were in line with the logistic regression. For example, in 2014, an average increase in age by one year had a predicted 0.03% higher chance of becoming an HCU. The average patient with neoplasms (a primary malignant tumour) had a predicted 8% higher chance of being an HCU.

The interactions of each of the potential factors associated with HCU status showed that individuals who died before their discharge having had diseases of the respiratory system (OR = 1.39–2.08) or of the digestive system (OR = 1.04–1.44) were more likely to be HCUs. Moreover, those who were admitted to a general hospital, community hospital, or other MOPH agencies in Bangkok were also more likely to be HCUs.

This study also examined potential identifying factors of individuals who were likely to remain HCUs over time. In the data, all the persistent HCUs were HCUs for at least two years; no patient was found to be an HCU for three consecutive years. From a descriptive analysis, the average annual cost was higher for persistent HCUs (52,867 ± 74,024) as compared to those who were once off HCUs (24,577 ± 50,495). In a multinomial logistic regression, the conditions that were associated with a higher likelihood of becoming a persistent HCU were similar to the factors associated with HCUs as shown in the Supplementary table 2. However, the magnitude of the impact differed in some cases. For example, the relative risk ratio (RRR) for neoplasms was 7.9 for HCUs, but was 43.5 for persistent HCUs. This finding suggests that patients with neoplasms were likely to be HCUs, and HCUs with neoplasms were also likely to be persistent HCUs.

DISCUSSION

There is a need for understanding the characteristics of HCUs with the highest need in terms of consuming healthcare resources and their underlying factors that may be influencing costs. Recognizing that among all HCUs, some may truly need those health services whereas some may not. This study first confirmed that the HCUs phenomenon existed in Thailand, an upper middle-income country, where a majority of inpatient care spending is concentrated in the top 5% of the hospitalized population; therefore, our results are comparable to the existing literature on this phenomenon globally.

For peer review only - http://bmjopen.bmj.com/site/about/guidelines.xhtml

The factors associated with HCUs appear to align with those reported in the international literature. Older patients and males were more likely to be HCUs than younger and female patients, as compared to a study by Rosella et al. in Canada.[16] HCUs were also likely to have multiple diagnoses, comorbidity conditions, and serious types of diagnoses (e.g. cancer and cardiovascular diseases), and therefore, they were likely to also be admitted to hospitals in Bangkok (the capital of Thailand) with more advanced treatments available. Having chronic conditions (e.g. cancer and respiratory diseases) also increased the probability of being a persistent HCU (i.e. more than one year) as found in the published literature of high-income OECD countries.[17] This study raises an interesting point in setting health priority using disease burden that has been relied on morbidity and mortality outcomes in terms of disability-adjusted life-years (DALY)[35] though this measure did not take into account burden of care that was measured in this study. For example, although injury is one of the highest disease burdens in Thailand,[25] there are different types of injury; and many may incur low cost whereas some has high cost associated with it. Also, injury in Thailand is often occurred among young population and associated with lower average number of visits as compared to other diseases. Injury becomes a contributing factor of HCUs when adjusting other explanatory variables as shown in Table 3. This is incontrary to cardiovascular diseases and neoplasms, for example, that are high in both disease burden and cost of care.

Furthermore, the actual costs of healthcare resources consumed by these hospitals should be explored to ensure that the expense paid by NHSO using the DRG approach is adequate for developing and maintaining referral systems, and preventing hospitals from rejecting referred patients.[36] Additionally, the DRG creep, which is a method of modifying clinical coding practices to maximize reimbursement, should be investigated as well.[29 37] Knowing the common diagnoses of HCUs could also underline the associated costs or burden of these health conditions. As frequent users of the healthcare system, HCUs would be expected to visit a hospital more than once a year, as the findings have shown. Information on these potential factors associated with HCUs could assist healthcare professionals and hospitals in creating more tailored treatments and management plans for patients who are likely to become HCUs.

This study includes multiple strengths and limitations for future research. To our knowledge, this study is the first to confirm the HCU phenomenon in UMICs, and it is hoped that the results could be applicable to other settings (with similar country profiles), especially low-income countries which may be facing limited resources and need evidence to improve the efficiency of their healthcare system. Knowing characteristics of those in need would equip the healthcare system to respond accordingly; for example, healthcare providers could

For peer review only - http://bmjopen.bmj.com/site/about/guidelines.xhtml

design more tailored and appropriate care to the patients. This research represents an example of how existing administrative databases could be used to answer policy-relevant questions.

This study highlights the importance of data quality in administrative databases. There were issues regarding the completeness of data between 2014 and 2018. Year 2014 was the first year that data were computerised; therefore, the data were not complete as the data entering had just started. Claims data from hospitals are usually collected by hospitals through medical charts (which can take time) and entered into NHSO system at a later date (after being checked for accuracy through the NHSO auditing steps); therefore, the data in 2018 are not completely integrated. Regardless, it is useful to include data from 2014 and 2018 to assist in investigating the magnitude and trend of HCUs. Moreover, the analyses encountered several inaccuracies, such as conflicting sexes or dates of birth of individuals with multiple hospitalizations. Therefore, it is integral that when linking across databases (hospitalization and death databases), a data quality check is put in place to ensure the accurate merging of data; this step should be standard practice for similar analyses.

Additionally, as a secondary data analysis, this study was limited by data availability. Consequently, there could be additional potential confounders which were not included due to lack of data (e.g. education and income); this is a limitation that future research should consider. For example, if we were able to link the database to an outpatient visit database, the analysis may be able to examine potential factors associated with HCUs at the ambulatory-care stage as well, considering rehabilitation and prevention and promotion programs. Consequently, patients who died before their discharge were also determined to have a higher chance of being HCUs. This factor (died before discharged) may not be as informative in assisting the development of treatment plans as it is related to the outcome. In the model without this death factor, other factors (mentioned above) remain to be significant in helping to identify potential HCUs. Only in-hospital mortality was included as hospital admission data for this study; thus, future research should consider national mortality data as well. In addition, selected high-cost specialty medicines and interventions (such as high-cost medicines E2 access program[30] and stents[38]) were not included in the analysis.These interventions are procured centrally by NHSO and distributed directly to hospitals. Therefore, they are not included in the claims data and could be explored in future research for more detailed results. Furthermore, the categorization of diagnoses was achieved based on the DRG system which thus led to rather broad categories. This approach was meant to represent initial exploration on common diagnoses among HCUs; therefore, future research could explore diagnoses in a more detailed manner. The current analysis could not distinguish between those who are in true need (for high-cost treatments) versus those

For peer review only - http://bmjopen.bmj.com/site/about/guidelines.xhtml

whom the healthcare system can support to avoid unnecessary hospitalization. Some diseases may incur substantial costs over a lifetime (e.g. cystic fibrosis), whereas others may incur very high costs in a short amount of time. Further analysis to incorporate these details could add to the understanding of HCUs and, subsequently, further assist the planning of healthcare resources, including highlighting the importance of having a data system with long-term data which allows for exploration of long-term health and economic impacts.

These findings could be applied to several scenarios to support policy-making processes. For example, knowing who the HCUs are could help health professionals monitor the magnitude and trend of HCUs and consider the HCU phenomenon when developing relevant policies such as UCS reimbursement policies to reduce avoidable HCU or improve the outcomes of high-cost interventions. Moreover, a mechanism could be built to support tertiary hospitals in identifying HCUs upon admission and developing measures to support them on a routine basis as opposed to ad hoc monitoring. This mechanism can also make it easier to consider whether to provide aggressive treatment to patients with low probabilities of survival (e.g. higher Charlson comorbidity score or with multiple organ failure), especially in palliative care management. Furthermore, public communications should be made to individuals in society to raise awareness and encourage the prevention of risk factors through publicly funded prevention and promotion (P&P) programs in support of avoiding future comorbidities or other chronic diseases (e.g. cancer).[39]

In addition, in the healthcare system without UHC, the Thai government could explore having people with risk factors of HCUs be one of the priority groups so they can monitor and ensure that the patients are receiving appropriate care before they become HCUs. Low-value care is also of concern in healthcare systems,[9 10 40] and a study of its usage among HCUs could explore the significance of these low-value care services in this population. Further work on referral systems across different levels of health facilities and other types of services (in addition to hospitalization), including how to provide appropriate care at the right time and right place, could provide evidence to assist policy-makers in their planning and delivery of health services. Exploration of HCUs and their transitions across years could further develop the understanding of those with expensive necessities.

CONCLUSION

This study confirmed the HCU phenomenon in Thailand under UCS and reported the characteristics of HCUs. Factors contributing to being HCUs were being male, older, having cormorbidities, longer hospital stays, higher annual number of visits, being admitted to multiple hospitals, and having primary diagnoses such as neoplasms, diseases of the circulatory

For peer review only - http://bmjopen.bmj.com/site/about/guidelines.xhtml

system, injury, or poisoning. Currently, HCUs (the top 5% of hospitalized patients) consumed almost 50% of the healthcare expenditure each year in Thailand. With a better understanding of HCUs, healthcare managers and policy-makers may be more equipped to assist patients by providing more tailored and targeted interventions.

Acknowledgements

The authors would like to thank the National Health Security Office (including Ms. Kanjana Sirigomon, Ms. Jutatip Thungthong, and Mr. Poonchana Wareechi) for providing the data and support for this study; Dr. Lou Jing, a research fellow at Health Intervention and Policy Evaluation Research, Saw Swee Hock School of Public Health, National University of Singapore, for providing support for the data cleaning steps; and Mr. Sarin KC from HITAP for proofreading support.

Health Intervention and Technology Assessment Programme (HITAP) and Saw Swee Hock School of Public Health (SSHSPH) are part of the International Decision Support Initiative (https://www.idsihealth.org/), which supports countries to get the best value for money from health spending. International Decision Support Initiative receives funding support from the Bill & Melinda Gates Foundation, the UK Department for International Development, and the Rockefeller Foundation.

Contributors

The study was conceptualised by YT and WI. WR, WY, RB, NK, and JT contributed to its design. Funding was acquired by YT. JT was instrumental in providing data access. The analyses and interpretation were carried out by WR, WY, RB, under supervision of YT and WI. NK provided assistance with presentation of the findings. The initial draft was written by WR and WI, and refined with input from WY, RB, NK, JT and YT. All authors read and approved the final manuscript.

Funding

This study was supported by the Thailand Research Fund under the senior research scholar on Health Technology Assessment grant numbers [RTA5980011] and the Bill & Melinda Gates Foundation, the UK Department for International Development, and the Rockefeller Foundation grant number [OPP1202541] through the International Decision Support Initiative (https://www.idsihealth.org/).

For peer review only

Disclaimer

The findings, interpretations and conclusions expressed in this article are of the authors and do not necessarily reflect the views of the aforementioned funding agencies.

Competing interests

The authors declared no competing interests relevant to the submitted manuscript.

Ethics approval

The study did not involve any human participants and a research ethics approval was obtained for the secondary data analysis by Institute for the Development of Human Research Protections (IHRP No.795/2561, Date of approval on 22 November 2018). The research complied with the Non-disclosure agreement between National Health Security Office and Health Intervention and Technology Assessment Program to protect individual identification.

Data sharing statement

The data are not publicly available due to privacy or ethical restrictions according to the data sharing agreement with the National Health Security Office.

For peer review only

For peer review only - http://bmjopen.bmj.com/site/about/guidelines.xhtml

REFERENCES

1. Borgonovi E, Compagni A. Sustaining universal health coverage: The interaction of social, political, and economic sustainability. *Value Health* 2013;16(Supplement 1):S34-S38.

2. Kabaniha GA, Ataguba JE-O, Kutzin J. Global healthcare financing. In: Haring R, Kickbusch I, Ganten D, et al., editors. Handbook of global health. Cham: Springer 2020:1-42.

3. Abdirahman AS, Sagwa EV. Influence of resource mobilization on sustainability of universal health coverage in vulnerable livelihoods in Wajir county, Kenya. *JOPEA* 2020; 4(7):18-33.

4. Chunharas S. Making Thailand's UHC 'SAFE' (sustainable, affordable, fair and efficient). *Global Social Policy* 2020;20(2):231-36.

5. Ezoe S. Universal health coverage in japan: leveraging technologies to ensure equitable, affordable, and quality health care for all. In: Murthy P, Ansehl A, editors. Technology and Global Public Health. Cham: Springer 2020:388.

6. Tegama N. Sustainable resource mobilisation to strengthen global healthcare interventions: South African Institute of International Affairs, 2020. Available from: https://www.africaportal.org/publications/sustainable-resource-mobilisation-strengthen-global-healthcare-interventions/

7. Watkins DA, Jamison DT, Mills T, et al. Universal health coverage and essential packages of care. In: Jamison DT, Gelband H, et al., editors. Disease control priorities: improving health and reducing poverty. Washington, DC: The International Bank for Reconstruction and Development, The World Bank 2017.

8. Teerawattananon Y, Kingkaew P, Koopitakkajorn T, et al. Development of a health screening package under the universal health coverage: The role of health technology assessment. *Health Econ* 2016;25 (Supplement 1):162-78.

9. Badgery-Parker T, Pearson S-A, Chalmers K, et al. Low-value care in Australian public hospitals: prevalence and trends over time. *BMJ Qual Saf* 2019;28(3):205-14.

10. Schwartz AL, Landon BE, Elshaug AG, et al. Measuring low-value care in Medicare. *JAMA internal medicine* 2014;174(7):1067-76.

11. Breslow L. From disease prevention to health promotion. *JAMA* 1999;281(11):1030-3.

12. Labarthe DR, Dunbar SB. Global cardiovascular health promotion and disease prevention: 2011 and beyond. *Circulation* 2012;125(21):2667-76.

13. Calver J, Brameld KJ, Preen DB, et al. High-cost users of hospital beds in Western Australia: a population-based record linkage study. *Med J Aust* 2006;184(8):393-7.

For peer review only - http://bmjopen.bmj.com/site/about/guidelines.xhtml

14. Kozyrskyj A, Lix L, Dahl M, et al. High-cost users of pharmaceuticals: who are they? Winnipeg, MB: Manitoba Centre for Health Policy, 2005. Available from: http://mchp-appserv.cpe.umanitoba.ca/reference/high-cost.pdf

15. Rais S, Nazerian A, Ardal S, et al. High-cost users of Ontario's healthcare services. *Healthc Policy* 2013;9(1):44-51.

16. Rosella LC, Fitzpatrick T, Wodchis WP, et al. High-cost health care users in Ontario, Canada: demographic, socio-economic, and health status characteristics. *BMC Health Serv Res* 2014;14:532.

17. Tanke MAC, Feyman Y, Bernal-Delgado E, et al. A challenge to all. A primer on inter-country differences of high-need, high-cost patients. *PLoS One* 2019;14(6):e0217353.

18. Wodchis WP, Austin PC, Henry DA. A 3-year study of high-cost users of health care. *CMAJ* 2016;188(3):182-88.

19. Brinda EM, Kowal P, Attermann J, et al. Health service use, out-of-pocket payments and catastrophic health expenditure among older people in India: the WHO study on global ageing and adult health (SAGE). *J Epidemiol Community Health* 2015;69(5):489-94.

20. Peltzer K, Williams JS, Kowal P, et al. Universal health coverage in emerging economies: findings on health care utilization by older adults in China, Ghana, India, Mexico, the Russian Federation, and South Africa. *Global health action* 2014;7:25314.

21. Ruiz R, Strasser-Weippl K, Touya D, et al. Improving access to high-cost cancer drugs in Latin America: Much to be done. *Cancer* 2017;123(8):1313-23.

22. Tangcharoensathien V, Witthayapipopsakul W, Panichkriangkrai W, et al. Health systems development in Thailand: a solid platform for successful implementation of universal health coverage. *Lancet* 2018;391(10126):1205-23.

23. National Statistical Office. Size and structure of the population by age and sex, 2020 Available from: http://statbbi.nso.go.th/staticreport/page/sector/en/01.aspx

24. Office of the National Economic and Social Development Council. Report of the population projections for Thailand 2010-2040 (revision): Office of the National Economic and Social Development Council, 2019. Available from: http://statbbi.nso.go.th/staticreport/page/sector/en/01.aspx

25. The Thai Working Group on Burden of Disease and Injury. Burden of disease and injury of Thai population 2014. Nonthaburi: International Health Policy Program, Ministry of Public Health 2017.

26. Hanvoravongchai P. Health financing reform in Thailand: toward universal coverage under fiscal constraints: The World Bank, 2013 Available from: http://hdl.handle.net/10986/13298

For peer review only

For peer review only - http://bmjopen.bmj.com/site/about/guidelines.xhtml

27. Mohara A, Youngkong S, Velasco RP, et al. Using health technology assessment for informing coverage decisions in Thailand. *J Comp Eff Res* 2012;1(2):137-46.
28. Pannarunothai S. Diagnosis related groups (DRGs) development in Thailand. In: Pramualratana P, Wibulpolpraser S, editors. Health insurance systems in Thailand. Nonthaburi: Health Systems Research Institute 2002:143-54.
29. Pongpirul K, Walker DG, Winch PJ, et al. A qualitative study of DRG coding practice in hospitals under the Thai Universal Coverage Scheme. *BMC Health Serv Res* 2011;11(1):71.
30. Sruamsiri R, Wagner AK, Ross-Degnan D, et al. Expanding access to high-cost medicines through the E2 access program in Thailand: effects on utilisation, health outcomes and cost using an interrupted time-series analysis. *BMJ open* 2016;6(3):e008671.
31. Isaranuwatchai W, Teerawattananon Y, Archer RA, et al. Prevention of non-communicable disease: best buys, wasted buys, and contestable buys. *BMJ* 2020;368
32. World Health Organization. Tackling NCDs: 'best buys' and other recommended interventions for the prevention and control of noncommunicable diseases: World Health Organization, 2017. Available from: https://apps.who.int/iris/handle/10665/259232
33. Charlson M, Szatrowski TP, Peterson J, et al. Validation of a combined comorbidity index. *J Clin Epidemiol* 1994;47(11):1245-51.
34. D'Hoore W, Bouckaert A, Tilquin C. Practical considerations on the use of the Charlson comorbidity index with administrative data bases. *J Clin Epidemiol* 1996;49(12):1429-33.
35. Bundhamcharoen K, Odton P, Phulkerd S, et al. Burden of disease in Thailand: changes in health gap between 1999 and 2004. *BMC Public Health* 2011;11(1):1-9.
36. Sriratanaban J. Impact of universal coverage policy implementation on public hospitals, and their responses that affect medical services: Health Systems Research Institute, 2012. Available from: https://www.hsri.or.th/sites/default/files/browse/tor5-1-2.pdf
37. Pongpirul K, Walker DG, Rahman H, et al. DRG coding practice: a nationwide hospital survey in Thailand. *BMC Health Serv Res* 2011;11:290.
38. Ingun P, Boongerd P. Thailand health information system improvement through universal health coverage implementation. *J Thai Med Inform Assoc* 2015;2:137-47.
39. Faramnuayphol P. Impact of UCS on public health: Health Systems Research Institute; 2012. Available from: https://www.hsri.or.th/sites/default/files/browse/tor5-1-3.pdf

For peer review only

40. Bhatia RS, Levinson W, Shortt S, et al. Measuring the effect of Choosing Wisely: an integrated framework to assess campaign impact on low-value care. *BMJ Qual Saf* 2015;24(8):523-31.

For peer review only

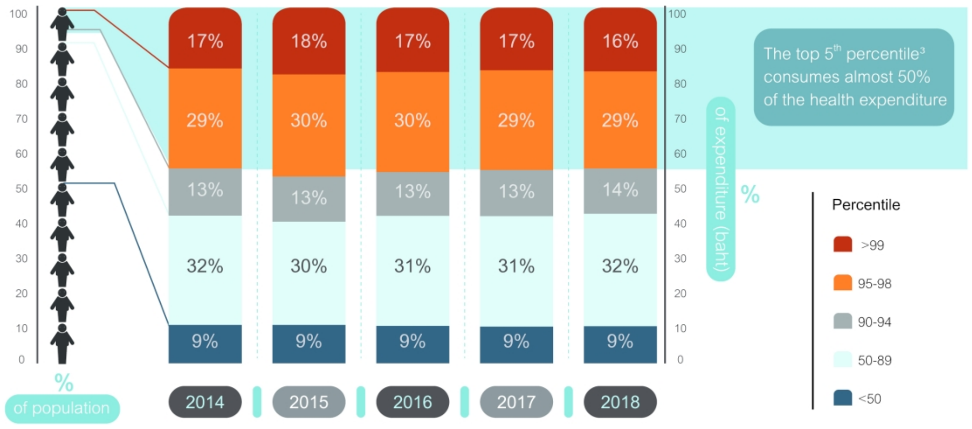

Figure 1 Distribution of total hospitalization's expenditures across cost percentiles

Supplementary figure 1 Different health regions in Thailand

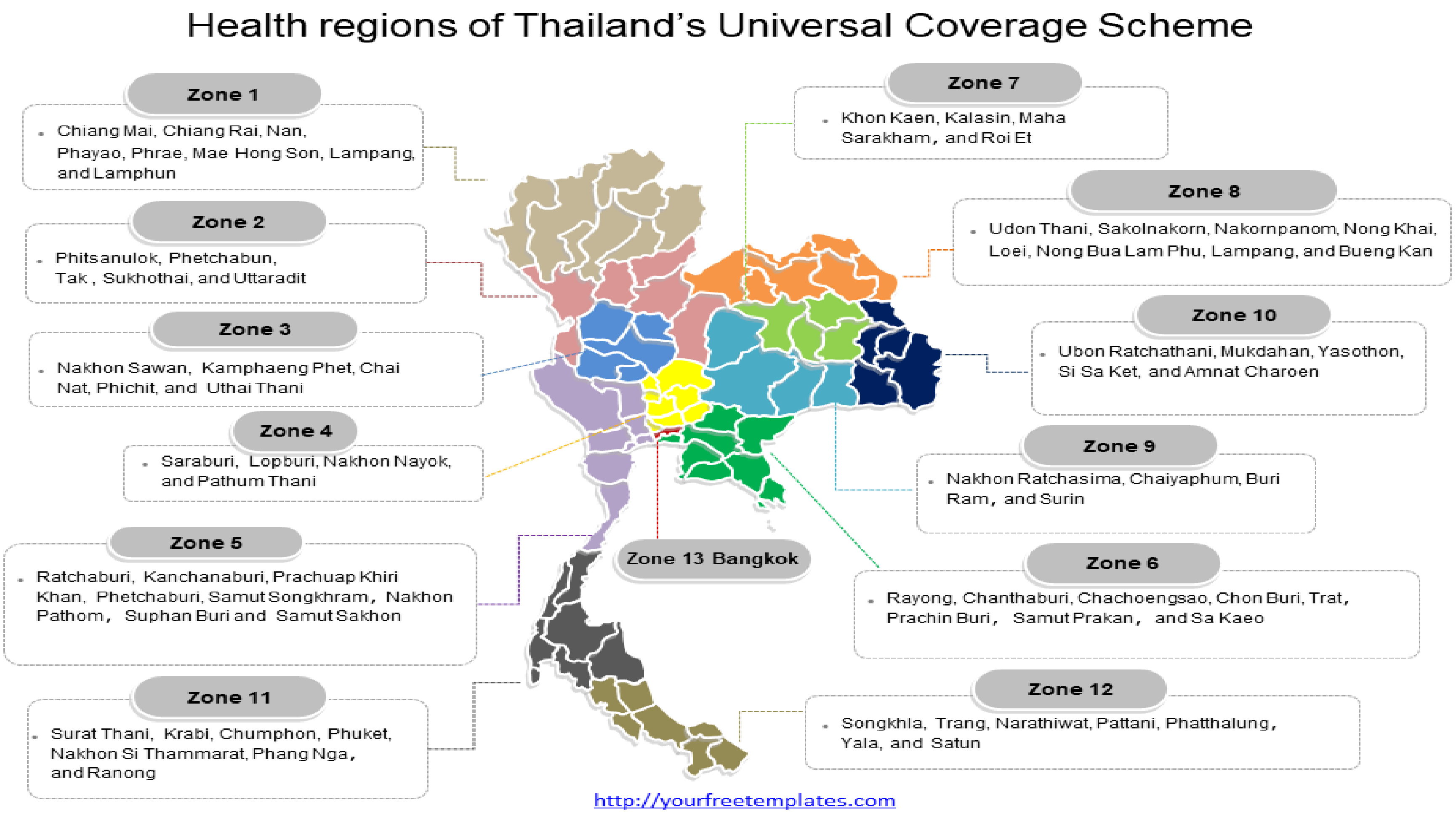

Supplementary figure 2 Flowchart of the data management process

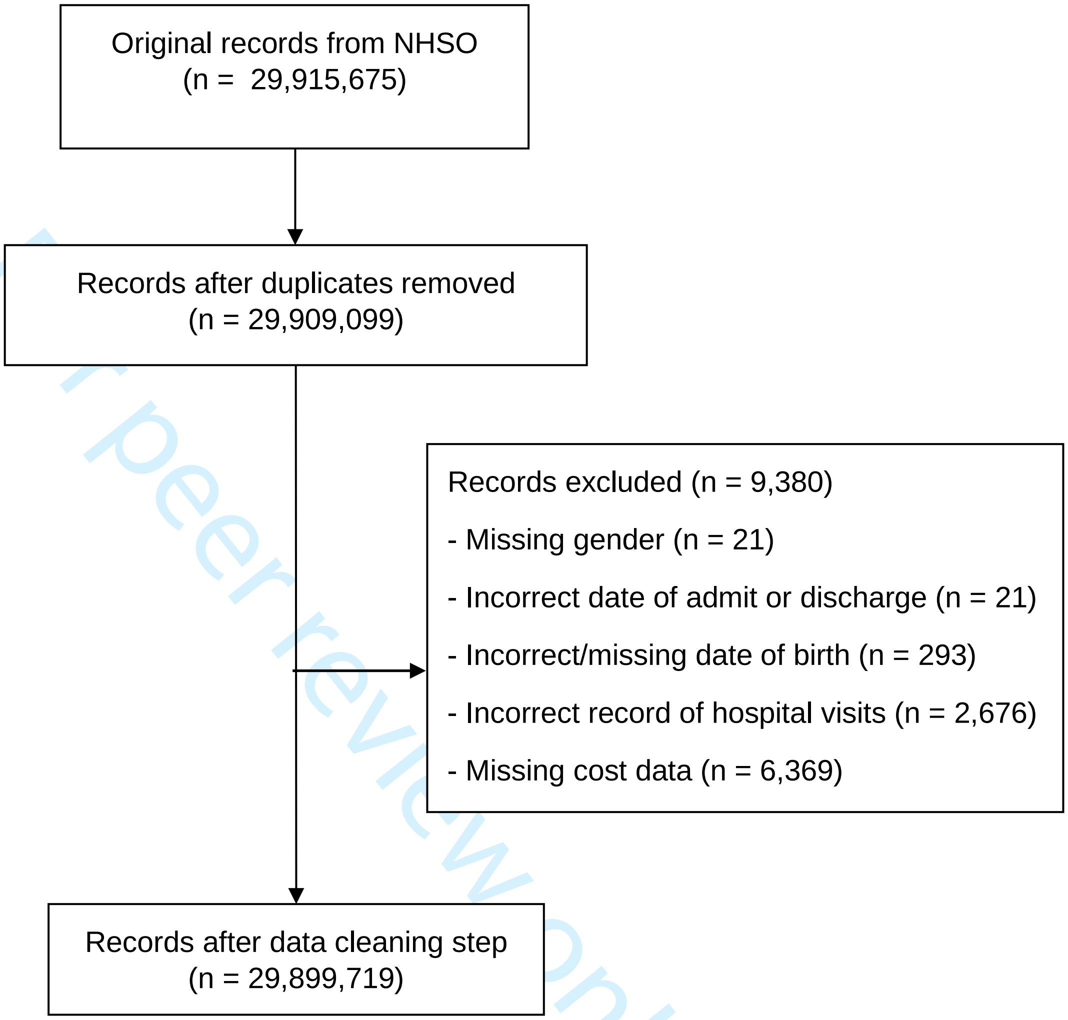

Supplementary table 1 Marginal effect of Predictors associated with high-cost users in UCS

	2013	2014	2015	2016	2017
	Prob (95%CI)	Prob (95%CI)	Prob (95%CI)	Prob (95%CI)	Prob (95%CI)
Age	0.00 (0.00, 0.00)	0.00 (0.00, 0.00)	0.00 (0.00, 0.00)	0.00 (0.00, 0.00)	0.00 (0.00, 0.00)
Gender (ref: female)	-0.01 (-0.01, -0.01)	-0.01 (-0.01, -0.01)	-0.01 (-0.01, -0.01)	-0.01 (-0.01, -0.01)	-0.01 (-0.01, -0.00)
Regional hospitals	0.05 (0.05, 0.05)	0.05 (0.05, 0.05)	0.05 (0.05, 0.05)	0.05 (0.05, 0.05)	0.05 (0.05, 0.05)
Hospital type (ref: other clinics)					
General hospitals, community hospitals, and other MOPH agencies	0.00 (0.00, 0.00)	0.01 (0.00, 0.01)	0.00 (0.00, 0.00)	0.00 (0.00, 0.00)	0.00 (0.00, 0.00)
Non MOPH agencies such as university hospitals	0.11 (0.10, 0.11)	0.13 (0.13, 0.14)	0.12 (0.12, 0.12)	0.11 (0.11, 0.12)	0.11 (0.11, 0.11)
Private hospitals	0.03 (0.03, 0.03)	0.07 (0.06, 0.07)	0.06 (0.06, 0.06)	0.05 (0.05, 0.06)	0.05 (0.05, 0.06)
Hospital zone: (ref: hospitals outside Bangkok)					
Bangkok	0.06 (0.06, 0.06)	0.09 (0.09, 0.09)	0.07 (0.07, 0.08)	0.07 (0.07, 0.08)	0.07 (0.06, 0.07)
Primary diagnosis					
Neoplasm	0.08 (0.08, 0.08)	0.08 (0.08, 0.08)	0.07 (0.07, 0.07)	0.07 (0.07, 0.08)	0.08 (0.07, 0.08)
Diseases of circulatory system	0.07 (0.07, 0.07)	0.07 (0.07, 0.08)	0.08 (0.08, 0.08)	0.08 (0.08, 0.08)	0.08 (0.08, 0.08)
Diseases of respiratory system	0.02 (0.02, 0.02)	0.01 (0.01, 0.01)	0.01 (0.01, 0.01)	0.01 (0.01, 0.01)	0.01 (0.01, 0.01)
Diseases of the digestive system	-0.00 (-0.00, -0.00)	-0.00 (-0.00, -0.00)	-0.00 (-0.00, -0.00)	-0.01 (-0.01, -0.01)	-0.00 (-0.00, -0.00)
Injury, or poisoning and certain other consequences of external causes	0.04 (0.04, 0.04)	0.05 (0.05, 0.05)	0.04 (0.04, 0.04)	0.04 (0.04, 0.04)	0.04 (0.04, 0.04)
Death	0.1 (0.10, 0.11)	0.08 (0.08, 0.09)	0.09 (0.08, 0.09)	0.07 (0.07, 0.08)	0.07 (0.06, 0.07)
Number of primary diagnoses	0.01 (0.01, 0.01)	0.01 (0.01, 0.01)	0.01 (0.01, 0.01)	0.01 (0.01, 0.01)	0.01 (0.01, 0.01)
Number of visits in a year	0.02 (0.02, 0.02)	0.02 (0.02, 0.02)	0.02 (0.02, 0.02)	0.02 (0.02, 0.02)	0.02 (0.02, 0.02)

Notes: Prob = Probability; 95% CI = 95% confidence interval

For peer review only

Supplementary table 2 Relative risk ratio of factors predicting being high-cost users and persistent high-cost users

Factors	Factors predicting being HCUs<br>RRR (95% CI)	Factors predicting being persistent HCUs<br>RRR (95% CI)
Age	1.04 (1.04, 1.04)	1.08 (1.08, 1.09)
Age*Age	0.99 (0.99, 0.99)	0.99 (0.99, 0.99)
Gender (ref: female)	0.69 (0.68, 0.70)	0.80 (0.77, 0.83)
Hospital type (ref: other clinics)		
Regional hospitals	2.83 (2.8, 2.86)	3.19 (3.12, 3.26)
General hospitals, community hospitals, and other MOPH agencies	1.09 (1.07, 1.10)	0.86 (0.83, 0.88)
Non MOPH agencies such as university hospitals	5.56 (5.46, 5.66)	10.2 (9.93, 10.5)
Private hospitals	2.29 (2.23, 2.35)	3.18 (3.04, 3.32)
Hospital zone: (ref: hospitals located outside Bangkok)		
Bangkok	4.40 (4.24, 4.57)	8.15 (7.63, 8.71)
Primary diagnosis		
Neoplasm	7.91 (7.54, 8.31)	43.5 (40.3, 47.0)
Diseases of circulatory system	7.24 (6.96, 7.53)	12.4 (11.5, 13.4)
Diseases of respiratory system	1.30 (1.27, 1.33)	2.21 (2.08, 2.35)
Diseases of the digestive system	0.73 (0.7, 0.75)	0.95 (0.89, 1.02)
Injury, or poisoning and certain other consequences of external causes	1.99 (1.93, 2.04)	2.78 (2.6, 2.97)
Comorbidity (Charlson comorbidity index)	1.12 (1.12, 1.13)	1.21 (1.20, 1.22)
Death	3.03 (2.93, 3.14)	3.63 (3.43, 3.85)
Number of primary diagnoses	1.71 (1.70, 1.71)	1.75 (1.74, 1.77)
Number of visits in a year	1.06 (1.06, 1.06)	1.16 (1.16, 1.17)
Neoplasm*Death	0.56 (0.54, 0.59)	0.60 (0.56, 0.64)
Diseases of circulatory system*Death	0.51 (0.49, 0.52)	0.55 (0.53, 0.59)
Diseases of respiratory system*Death	1.39 (1.34, 1.44)	1.79 (1.70, 1.88)
Diseases of the digestive system*Death	1.12 (1.08, 1.16)	1.22 (1.16, 1.29)
Injury, or poisoning and certain other consequences of external causes*Death	0.68 (0.65, 0.71)	0.77 (0.72, 0.82)
Neoplasm*Age	0.98 (0.98, 0.98)	0.96 (0.96, 0.97)
Diseases of circulatory system*Age	0.98 (0.98, 0.98)	0.98 (0.97, 0.98)
Diseases of respiratory system*Age	0.99 (0.99, 0.99)	0.99 (0.99, 0.99)
Diseases of the digestive system*Age	1.00 (1.00, 1.00)	0.99 (0.99, 0.99)
Injury, or poisoning and certain other consequences of external causes*Age	0.99 (0.99, 0.99)	0.99 (0.99, 0.99)

For peer review only

Factors	Factors predicting being HCUs RRR (95% CI)	Factors predicting being persistent HCUs RRR (95% CI)
Neoplasm*Gender	0.84 (0.82, 0.87)	0.69 (0.66, 0.72)
Diseases of circulatory system*Gender	1.01 (0.99, 1.03)	0.83 (0.80, 0.87)
Diseases of respiratory system*Gender	0.95 (0.93, 0.97)	0.82 (0.79, 0.86)
Diseases of the digestive system*Gender	1.06 (1.04, 1.09)	0.97 (0.93, 1.00)
Injury, or poisoning and certain other consequences of external causes*Gender	1.19 (1.16, 1.22)	1.23 (1.18, 1.28)
Hospital zone: Bangkok* Regional hospitals	0.55 (0.53, 0.57)	0.52 (0.49, 0.55)
Hospital zone: Bangkok* General hospitals, community hospitals, and other MOPH agencies	1.31 (1.27, 1.35)	1.56 (1.48, 1.65)
Hospital zone: Bangkok* Non MOPH agencies	0.28 (0.27, 0.29)	0.19 (0.18, 0.20)
Hospital zone: Bangkok* Private hospitals	0.42 (0.40, 0.44)	0.30 (0.28, 0.32)
Constant	0.00 (0.00, 0.00)	0.00 (0.00, 0.00)

Notes: 95% CI = 95% confidence interval; HCUs = high cost users; RRR = relative risk ratio.

For peer review only

STROBE 2007 (v4) Statement—Checklist of items that should be included in reports of *cross-sectional studies*

Section/Topic	Item #	Recommendation	Reported on page #
**Title and abstract**	1	(*a*) Indicate the study's design with a commonly used term in the title or the abstract	2
		(*b*) Provide in the abstract an informative and balanced summary of what was done and what was found	2
**Introduction**			
Background/rationale	2	Explain the scientific background and rationale for the investigation being reported	4-5
Objectives	3	State specific objectives, including any prespecified hypotheses	5
**Methods**			
Study design	4	Present key elements of study design early in the paper	5
Setting	5	Describe the setting, locations, and relevant dates, including periods of recruitment, exposure, follow-up, and data collection	5
Participants	6	(*a*) Give the eligibility criteria, and the sources and methods of selection of participants	5-7
Variables	7	Clearly define all outcomes, exposures, predictors, potential confounders, and effect modifiers. Give diagnostic criteria, if applicable	5-7
Data sources/ measurement	8*	For each variable of interest, give sources of data and details of methods of assessment (measurement). Describe comparability of assessment methods if there is more than one group	5-6
Bias	9	Describe any efforts to address potential sources of bias	5-7
Study size	10	Explain how the study size was arrived at	5-6
Quantitative variables	11	Explain how quantitative variables were handled in the analyses. If applicable, describe which groupings were chosen and why	5-6
Statistical methods	12	(*a*) Describe all statistical methods, including those used to control for confounding	6-7
		(*b*) Describe any methods used to examine subgroups and interactions	6-7
		(*c*) Explain how missing data were addressed	5
		(*d*) If applicable, describe analytical methods taking account of sampling strategy	N/A
		(*e*) Describe any sensitivity analyses	N/A
**Results**			

For peer review only

Participants	13*	(a) Report numbers of individuals at each stage of study—eg numbers potentially eligible, examined for eligibility, confirmed eligible, included in the study, completing follow-up, and analysed	7
		(b) Give reasons for non-participation at each stage	N/A
		(c) Consider use of a flow diagram	N/A
Descriptive data	14*	(a) Give characteristics of study participants (eg demographic, clinical, social) and information on exposures and potential confounders	7-10
		(b) Indicate number of participants with missing data for each variable of interest	7
Outcome data	15*	Report numbers of outcome events or summary measures	7-11
Main results	16	(*a*) Give unadjusted estimates and, if applicable, confounder-adjusted estimates and their precision (eg, 95% confidence interval). Make clear which confounders were adjusted for and why they were included	7-16
		(*b*) Report category boundaries when continuous variables were categorized	7-16
		(*c*) If relevant, consider translating estimates of relative risk into absolute risk for a meaningful time period	N/A
Other analyses	17	Report other analyses done—eg analyses of subgroups and interactions, and sensitivity analyses	N/A
**Discussion**			
Key results	18	Summarise key results with reference to study objectives	16-18
Limitations	19	Discuss limitations of the study, taking into account sources of potential bias or imprecision. Discuss both direction and magnitude of any potential bias	17-18
Interpretation	20	Give a cautious overall interpretation of results considering objectives, limitations, multiplicity of analyses, results from similar studies, and other relevant evidence	16-18
Generalisability	21	Discuss the generalisability (external validity) of the study results	16-18
**Other information**			
Funding	22	Give the source of funding and the role of the funders for the present study and, if applicable, for the original study on which the present article is based	19

*Give information separately for cases and controls in case-control studies and, if applicable, for exposed and unexposed groups in cohort and cross-sectional studies.

Note: An Explanation and Elaboration article discusses each checklist item and gives methodological background and published examples of transparent reporting. The STROBE checklist is best used in conjunction with this article (freely available on the Web sites of PLoS Medicine at http://www.plosmedicine.org/, Annals of Internal Medicine at http://www.annals.org/, and Epidemiology at http://www.epidem.com/). Information on the STROBE Initiative is available at www.strobe-statement.org.

For peer review only
